# The Rise and Fall of an Evolutionary Innovation: Contrasting Strategies of Venom Evolution in Ancient and Young Animals

**DOI:** 10.1371/journal.pgen.1005596

**Published:** 2015-10-22

**Authors:** Kartik Sunagar, Yehu Moran

**Affiliations:** Department of Ecology, Evolution and Behavior, The Alexander Silberman Institute for Life Sciences, Hebrew University of Jerusalem, Jerusalem, Israel; Harvard University, UNITED STATES

## Abstract

Animal venoms are theorized to evolve under the significant influence of positive Darwinian selection in a chemical arms race scenario, where the evolution of venom resistance in prey and the invention of potent venom in the secreting animal exert reciprocal selection pressures. Venom research to date has mainly focused on evolutionarily younger lineages, such as snakes and cone snails, while mostly neglecting ancient clades (e.g., cnidarians, coleoids, spiders and centipedes). By examining genome, venom-gland transcriptome and sequences from the public repositories, we report the molecular evolutionary regimes of several centipede and spider toxin families, which surprisingly accumulated low-levels of sequence variations, despite their long evolutionary histories. Molecular evolutionary assessment of over 3500 nucleotide sequences from 85 toxin families spanning the breadth of the animal kingdom has unraveled a contrasting evolutionary strategy employed by ancient and evolutionarily young clades. We show that the venoms of ancient lineages remarkably evolve under the heavy constraints of negative selection, while toxin families in lineages that originated relatively recently rapidly diversify under the influence of positive selection. We propose that animal venoms mostly employ a ‘two-speed’ mode of evolution, where the major influence of diversifying selection accompanies the earlier stages of ecological specialization (e.g., diet and range expansion) in the evolutionary history of the species–the period of expansion, resulting in the rapid diversification of the venom arsenal, followed by longer periods of purifying selection that preserve the potent toxin pharmacopeia–the period of purification and fixation. However, species in the period of purification may re-enter the period of expansion upon experiencing a major shift in ecology or environment. Thus, we highlight for the first time the significant roles of purifying and episodic selections in shaping animal venoms.

## Author Summary

While the influence of positive selection in diversifying animal venoms is widely recognized, the role of purifying selection that conserves the amino acid sequence of venom components such as peptide toxins has never been considered. In addition to unraveling the unique strategies of evolution of toxin gene families in centipedes and spiders, which are amongst the first terrestrial venomous lineages, we highlight the significant role of purifying selection in shaping the composition of animal venoms. Analysis of numerous toxin families, spanning the breadth of the animal kingdom, has revealed a striking contrast between the evolution of venom in ancient and evolutionarily young animal groups. Our findings enable the postulation of a new theory of venom evolution. The proposed ‘two-speed’ mode of evolution of venom captures the fascinating evolutionary history and the dynamics of this complex biochemical cocktail.

## Introduction

**Fig 1:**
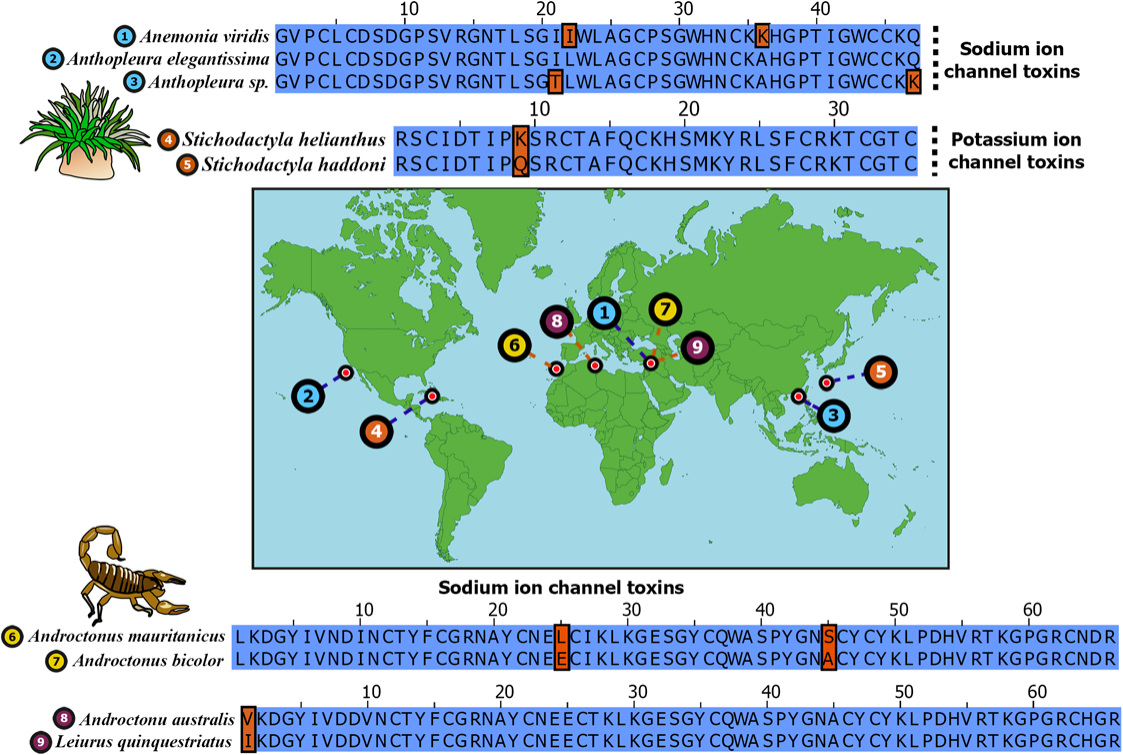
Remarkable sequence conservation in distantly related toxins. Sequence alignments of widely separated sea anemone and scorpion neurotoxins are depicted. Sampled locations of these toxins are indicated on the map. Identical positions in sequence alignments are shown in blue, while differing amino acids are shown in brown. Uniprot IDs of sequences are: 1) B1NWR0; 2) P01532; 3) P0C5F4; 4) P29187; 5) E2S062; 6) Q7YXD3; 7) D5HR48; 8) P01484 and 9) D5HR56.

Venom is an intriguing evolutionary innovation that is utilized by various animals for predation and/or defense. This complex biochemical cocktail is characterized by a myriad of organic and inorganic molecules, such as proteins, peptides, polyamines and salts that disrupt the normal physiology of the envenomed animal. Evolution of venom has been intensively investigated in more recently diverged lineages (for simplicity, we refer to them as ‘evolutionarily younger’ lineages), such as advanced snakes and cone snails, which originated ~54 [1] and ~33–50 [2, 3] million years ago (MA), respectively. Several venom-encoding genes in these animals have undergone extensive duplications [4, 5] and evolve rapidly under the influence of positive selection [6–10]. In contrast, the evolution of venom in most of the ancient lineages, such as cnidarians (corals, sea anemones, hydroids and jellyfish), coleoids (octopus, squids and cuttlefish), spiders and centipedes, remains understudied, if not completely overlooked. Perhaps the only exhaustively investigated ancient venomous clade are the scorpions, which originated in the Silurian about 430 MA [11, 12]. Moreover, certain potent toxins in species separated by considerable geographic and genetic distance can exhibit remarkable sequence conservation (Fig 1). Yet, research to date has solely focused on how positive selection has expanded the venom arsenal, while completely ignoring the role of negative (purifying) selection.

Phylum Cnidaria consists of animals such as sea anemones, jellyfish, corals and hyrdroids that originated in the Ediacaran Period, approximately 600 MA [13–15]. They are characterized by unique stinging organelles called nematocysts with which they inject venom. Cnidaria represents the oldest venomous lineage known and includes some of the most notorious animals, such as the sea wasp (*Chironex fleckeri*), a species of box jellyfish. Coleoids, which first appeared in the Early Devonian 380–390 MA [16], represent yet another neglected lineage of ancient venomous animals. Although the venomous nature of coleoids was established as early as 1888 [17], their venoms have received scant attention from toxinological research [17–20].

Centipedes are amongst the oldest living terrestrial venomous animals, with the fossil record extending back to ~420 MA [21]. All ~3,300 species of centipedes (class Chilopoda) described to date belong to five extant orders: Craterostigmomorpha, Geophilomorpha, Lithobiomorpha, Scolopendromorpha and Scutigeromorpha. They inject venom into victims via modified first pair of trunk limbs (forcipules) and use venom for predation and defense. Venoms of certain centipedes can cause excruciating pain, paresthesia, edema, necrosis [22, 23] and can be fatal to mammals as large as dogs [24]. Yet, only a handful of centipede toxins have been pharmacologically characterized to date. Similarly, despite their remarkable ability to target a diversity of ion channels, only toxins from certain medically significant species of spiders have been investigated to date [25]. Thus, the evolutionary history and phyletic distribution of venom from these aforementioned ancient lineages, which represent the first venomous animal groups, remain understudied [18, 26–28]. It should be noted that the divergence times of these lineages can be safely assumed to be equivalent to the time of origin of venom in those respective lineages, as all of the examined lineages are (i) venomous, (ii) do not share between them a common venomous ancestor, and/or (iii) for most of them the fossil data clearly indicates the presence of a venom delivery apparatus [29–36].

By examining a large number of nucleotide sequences from a diversity of species, we report for the first time the molecular evolutionary histories of a number of venom protein families in centipedes, spiders and Toxicofera (clade of venomous snakes and lizards) lizards. In contrast to the rapid evolution of venom in evolutionarily younger lineages, we report an unusually high conservation of venom in centipedes and spiders, despite their long evolutionary histories. Moreover, molecular evolutionary assessments of toxin-encoding genes distributed across the tree of life, has unraveled a surprisingly strong influence of negative selection on the venoms of ancient animals. Our findings reveal contrasting trajectories of venom evolution in ancient and evolutionarily young clades, and emphasize the significant roles of purifying and episodic selections in shaping animal venoms. Further, these results enabled the postulation of a new model of venom evolution that captures their evolutionary dynamics, and the rise and fall in evolutionary rates of animal venoms.

## Results

### Slow evolving centipede and spider venoms

Despite the fact that several centipede and spider toxins are capable of exhibiting a diverse array of pharmacological effects, their venoms remain poorly studied. To date, very few studies have examined the evolutionary mechanisms responsible for the diversification of toxins in centipedes [27, 28] and spiders [37–40]. Hence, we assessed the molecular evolutionary regimes of 17 and 10 gene families encoding toxins in the major lineages of centipedes and spiders, respectively. We computed the ratio of non-synonymous (dN) to synonymous (dS) substitutions, called omega (ω), where ω greater than, less than or equal to one is characteristic of positive, negative and neutral selection, respectively. A large proportion of centipede venoms are characterized by β-pore-forming toxins (β-PFT) that are similar to aerolysins and epsilon toxins from bacteria [28]. They are theorized to be responsible for myotoxic and edematogenic activities of centipede venoms [23, 28, 41]. β-PFT has undergone substantial gene duplication and diversification in centipedes [28]. In contrast to venom-encoding genes in evolutionarily younger lineages that continue experiencing positive selection when they diversify via recurrent duplication events, we find that β-PFTs are evolutionarily extremely constrained under negative selection, as indicated by ω smaller one (Table 1). Centipede venoms are also chiefly constituted by cysteine-rich secretory proteins, antigen 5, and pathogenesis-related 1 (CAP) family members and toxins with low-density lipoprotein receptor Class A (LDLA) repeats [28]. While certain CAP proteins in the venoms of centipedes are characterized by trypsin inhibitory activities [42], the precise role of LDLAs remain unknown. We found that both these toxin classes have experienced a strong influence of purifying selection (Table 1).

**Table 1:** Molecular evolution of centipede PFT, CAP and LDLA venom proteins.

	FUBAR^a^	MEME Sites^b^	PAML^c^ (M8)
**β-pore-forming toxins**	ω>**1** ^d^: 1	21	0
	ω<**1** ^e^: 216		ω: 0.26
**CAP**	ω>**1** ^d^: 1	11	0
	ω<**1** ^e^: 127		ω: 0.24
**LDLA**	ω>**1** ^d^: 1	23	2
	ω<**1** ^e^: 81		(1+1)
			ω:0.41

**a:** Fast Unconstrained Bayesian AppRoximation

**b:** Sites detected as experiencing episodic diversifying selection (0.05 significance) by the Mixed Effects Model Evolution (MEME)

**c:** Positively selected sites detected by the Bayes Empirical Bayes approach implemented in M8. Sites detected at 0.99 and 0.95 significance are indicated in the parenthesis

**d:** number of sites under pervasive diversifying selection at the posterior probability ≥0.9 (FUBAR)

**e:** Number of sites under pervasive purifying selection at the posterior probability ≥0.9 (FUBAR)

**ω:** mean dN/dS

Scoloptoxin (SLPTX) is a family of cysteine-rich peptides found in the venoms of several centipedes, where different members exhibit a diversity of pharmacological activities [28]. SLPTX1 appears to be similar to insect peritrophic matrix proteins and has been theorized to be one of the most basally recruited toxins in centipedes [28]. Despite its long evolutionary history, this toxin exhibited lower-levels of sequence variations due to the influence of negative selection (Table 2). SLPTX10 and SLPTX15 families were reported to have undergone a functional radiation, where members exhibit neurotoxicity by targeting various voltage-gated ion channels: calcium (Ca_v_), potassium (K_v_) and sodium (Na_v_) ion channels [28]. Similarly, certain SLPTX11 family members are known for their anticoagulant and K_v_ channel inhibitory activities [28, 43]. We found that even these putatively potent toxins in centipedes were extremely well conserved under the influence of purifying selection (Table 2).

**Table 2:** Molecular evolution of scoloptoxins.

	FUBAR^a^	MEME Sites^b^	PAML^c^ (M8)
**SLPTX01**	ω>**1** ^d^: 1	1	1
	ω<**1** ^e^: 39		(0+1)
			ω:0.55
**SLPTX04**	ω>**1** ^d^: 0	2	0
	ω<**1** ^e^: 5		ω:0.40
**SLPTX05**	ω>**1** ^d^: 0	1	0
	ω<**1** ^e^: 34		ω:0.36
**SLPTX10**	ω>**1** ^d^: 1	9	0
	ω<**1** ^e^: 42		ω:0.33
**SLPTX11**	ω>**1** ^d^: 0	8	0
	ω<**1** ^e^: 57		ω:0.42
**SLPTX12**	ω>**1** ^d^: 0	1	0
	ω<**1** ^e^: 15		ω:0.21
**SLPTX13**	ω>**1** ^d^: 1	1	0
	ω<**1** ^e^: 12		ω:0.71
**SLPTX15**	ω>**1** ^d^: 0	4	0
	ω<**1** ^e^: 16		ω:0.36
**SLPTX16**	ω>**1** ^d^: 1	6	0
	ω<**1** ^e^: 52		ω:0.35
**SLPTX17**	ω>**1** ^d^: 0	4	0
	ω<**1** ^e^: 14		ω:0.63

**a:** Fast Unconstrained Bayesian AppRoximation

**b:** Sites detected as experiencing episodic diversifying selection (0.05 significance) by the Mixed Effects Model Evolution (MEME)

**c:** Positively selected sites detected by the Bayes Empirical Bayes approach implemented in M8. Sites detected at 0.99 and 0.95 significance are indicated in the parenthesis

**d:** number of sites under pervasive diversifying selection at the posterior probability ≥0.9 (FUBAR)

**e:** Number of sites under pervasive purifying selection at the posterior probability ≥0.9 (FUBAR)

**ω:** mean dN/dS

**Fig 2:**
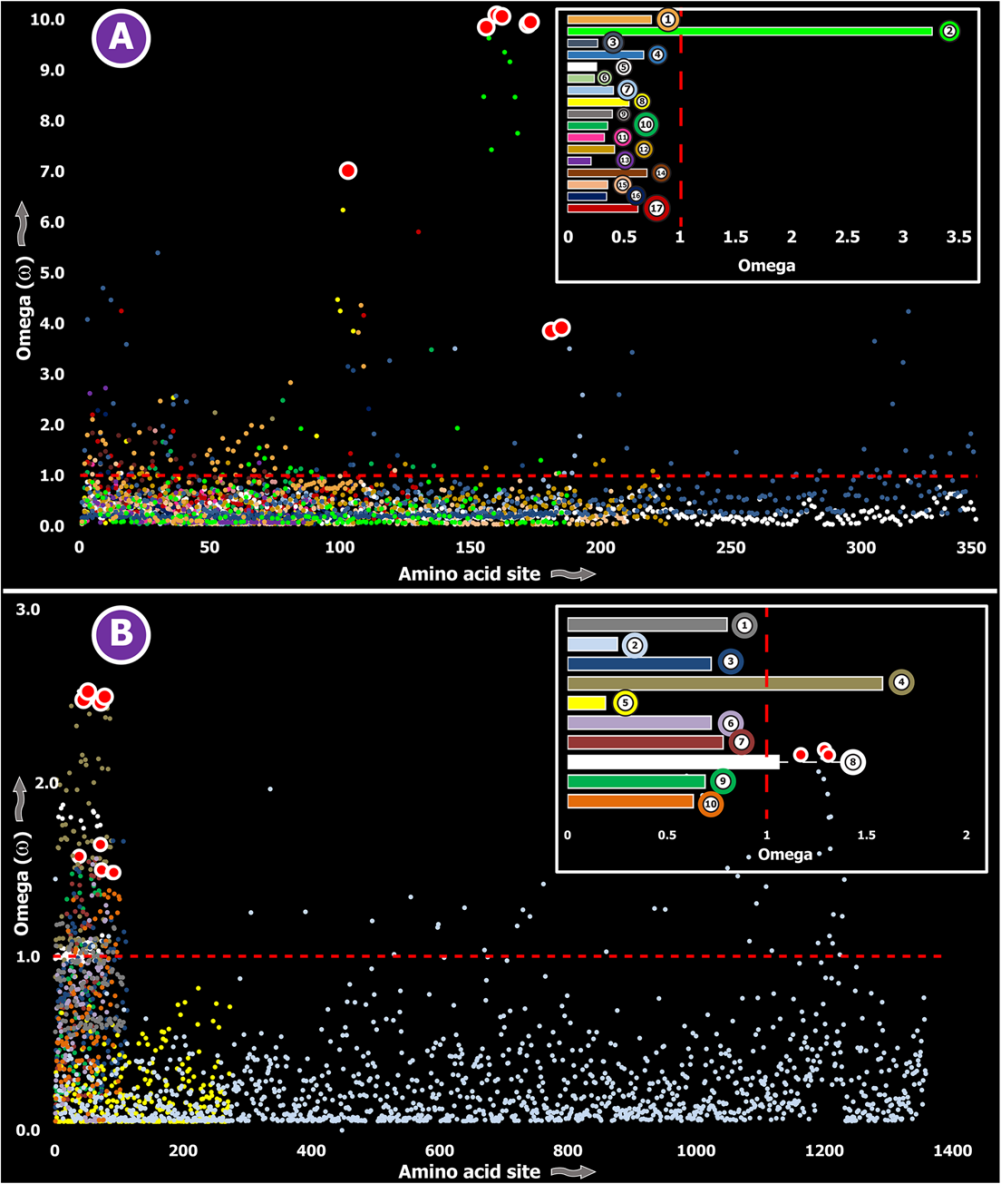
Molecular evolution of venom in centipedes (A) and spiders (B). A plot of site-specific ω against amino acid positions for various centipede and spider venom-encoding genes is presented in panel A and B, respectively. Significantly detected positively selected sites (model 8; Bayes Empirical Bayes approach) are presented as large red circles. The red horizontal line represents the line of neutrality: points above and below this line indicate positive and negative selection, respectively. A corresponding bar plot is provided, which shows the computed ω value for the respective toxin class. Bar plot color code: **Panel A** 1) Novel family (NF) 8; 2) NF 6; 3) NF 4; 4) NF 1; 5) β-PFT; 6) CAP; 7) LDLA; 8) SLPTX 1; 9) SLPTX 4; 10) SLPTX 5; 11) SLPTX 10; 12) SLPTX 11; 13) SLPTX 12; 14) SLPTX 13; 15) SLPTX 15; 16) SLPTX 16; 17) SLPTX 17; **Panel B** 1) lycotoxins; 2) latrotoxins; 3) magi-1 family; 4) Kunitz toxins; 5) Sphingomyelinase D; 6) Huwentoxin-1 family; 7) κ/ω-hexatoxins; 8) κ-hexatoxins; 9) ω-hexatoxins; 10) Superfamily E ICKs.

While SLPTX family 13 appears to have convergently adopted an inhibitory cysteine knot (ICK) scaffold, which is characteristic of various potent toxins from scorpions and spiders, SLPTX16 has adopted a Von Willebrand factor type C (VWC)-like domain. These peptides were highly conserved despite their putative role in prey envenoming and long evolutionary histories. Certain taxonomically restricted toxin families, called ‘novel families’ were recently reported in centipede venoms [28]. Only one (‘novel family 6’) amongst the four of these novel families examined was found to have evolved rapidly, while the rest were negatively selected (Table 3). Overall, centipede venom-encoding genes were found to have evolved under the heavy constraints of purifying selection (Fig 2A).

**Table 3:** Molecular evolution of centipede novel putative toxin families.

	FUBAR^a^	MEME Sites^b^	PAML^c^ (M8)
**Novel family 01**	ω>**1** ^d^: 0	3	0
	ω<**1** ^e^: 23		ω:0.68
**Novel family 04**	ω>**1** ^d^: 0	0	0
	ω<**1** ^e^: 3		ω:0.27
**Novel family 06**	ω>**1** ^d^: 0	0	5
	ω<**1** ^e^: 51		(1+4)
			ω:3.21
**Novel family 08**	ω>**1** ^d^: 0	0	0
	ω<**1** ^e^: 14		ω:0.75

**a:** Fast Unconstrained Bayesian AppRoximation

**b:** Sites detected as experiencing episodic diversifying selection (0.05 significance) by the Mixed Effects Model Evolution (MEME)

**c:** Positively selected sites detected by the Bayes Empirical Bayes approach implemented in M8. Sites detected at 0.99 and 0.95 significance are indicated in the parenthesis

**d:** number of sites under pervasive diversifying selection at the posterior probability ≥0.9 (FUBAR)

**e:** Number of sites under pervasive purifying selection at the posterior probability ≥0.9 (FUBAR)

**ω:** mean dN/dS

Spiders are known to have originated 416–359 MYA in the Devonian [44]. All spiders, with the exception of a few species, employ venom for predation. However, toxinological research to date has solely focused on characterizing venom from the medically significant species of spiders. Yet, venom from only 0.4% of the currently cataloged spider species have been characterized to date [25]. We determined the rate of evolution of several venom protein superfamilies in a diversity of spider lineages, such as the lethal latrotoxins secreted by widow spiders [Theridiidae: 223–180 MYA [45]]; Kunitz-type serine protease inhibitors and huwentoxins from tarantulas [Theraphosidae: 250–200 MYA [46]]; the magitoxin family from tarantulas and certain funnel-web spiders [Hexathelidae: 250–200 MYA [46]]; sphingomyelinase-D (SMase D) in the medically significant venoms of recluse spiders [Sicariidae: 145+ MYA [46]]; lycotoxin family [47] from wolf spiders [Lycosidae: ~120+ MYA [46]]; and super family E ICKs [48] secreted by tarantulas and brushed trapdoor spiders [Barychelidae: 250–200 MYA [46]]. These venom proteins are secreted in large amounts by the respective spider lineage and are known for a diversity of biochemical activities, such as insecticidal presynaptic neurotoxicity and the ability to stimulate neurotransmitter secretions [latrotoxins: [49, 50]], dermonecrotic properties [SMase D: [51]], Na_v_ channel targeting capability–with some members additionally capable of targeting Ca_v_ channels [huwentoxin-1 family: [52, 53]], serine protease inhibition and the ability to block K_v_ channels [Kunitz toxins: [54]], insect Na_v_ channel targeting [magi-1 family [55]], insect Ca_v_ channel targeting [ω-hexatoxins: [56]], insect Calcium activated potassium channel (KCa) targeting [κ-hexatoxins: [57]], and the Na_v_ modulation and Ca_v_ blocking capabilities [Super Family E ICKs: [48]]. The computed ω values suggested a greater influence of purifying selection on nine out of ten toxin families examined, highlighting the slower evolution of spider venoms (S1 Table; Fig 2B).

### Contrasting evolutionary regimes of venom in evolutionarily ancient and young clades

**Fig 3:**
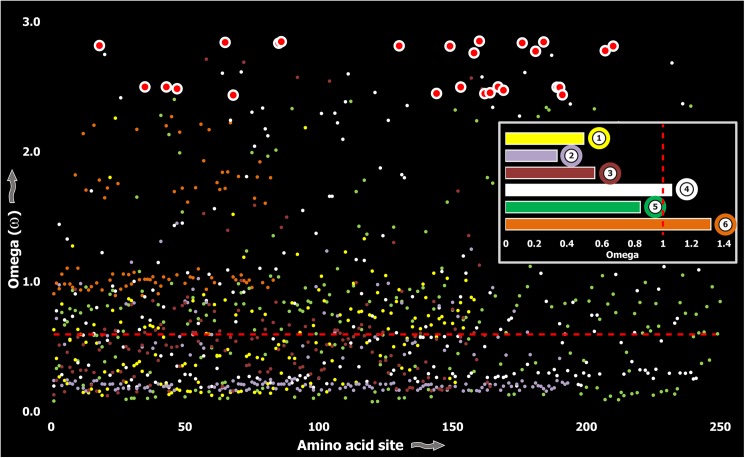
Molecular evolution of venom in Toxicofera lizards. A plot of site-specific ω against amino acid positions for various Toxicofera lizard toxin types is presented. The red horizontal line represents the line of neutrality: points above and below this line indicate positive and negative selection, respectively. Bar plot color codes: 1) Phospholipase A2; 2) Nerve Growth Factors; 3) Natriuretic peptides and 4) CRiSPs; 5) Kallikreins; and 6) crotamines.

Computed ω values for the vast majority of venom-encoding genes in all ancient lineages examined in this study and in previous studies [18, 26, 38, 58], highlighted the significant role of negative selection, which was in stark contrast to those of evolutionarily younger lineages, such as the advanced snakes and cone snails [S1 Table; [6, 7, 59–62]]. We also evaluated the molecular evolution of venom families from Toxicofera lizards that originated ~166 MYA [63], and thus represent an intermediate state between ancient and recently originated lineages. Although, relative to advanced snakes, these lizards do not rely on venom for predation or defense to the same degree [6], the evolutionary rates of some of their largely secreted venom proteins exhibited rapid evolution as demonstrated by their high number of positively selected sites (Fig 3; S1 Table).

**Fig 4:**
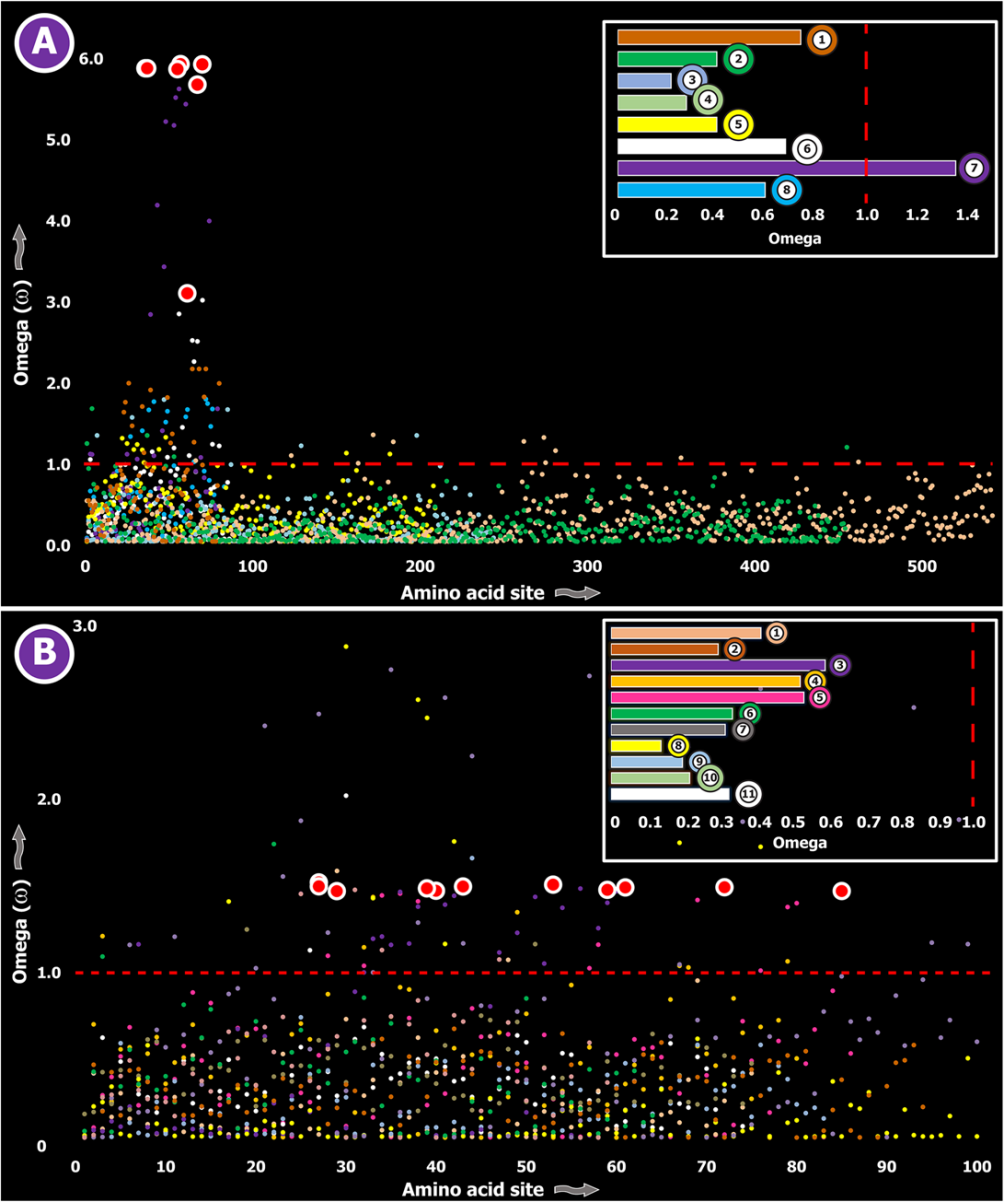
Molecular evolution of venom in cnidarians (A) and scorpions (B). A plot of site-specific ω against amino acid positions for various cnidarian and scorpion venom-encoding genes is presented in panel A and B, respectively. Significantly detected positively selected sites (model 8; Bayes Empirical Bayes approach) are presented as large red circles. The red horizontal line represents the line of neutrality: points above and below this line indicate positive and negative selection, respectively. A corresponding bar plot is provided, which shows the computed ω value for the respective toxin class. Bar-plot color code: **Panel A** 1) SCRiPs; 2) JFTs; 3) Hydralysins; 4) Aerolysin-related toxins in sea anemone; 5) Actinoporins; 6) KTx Type 1; 7) KTx Type 3; 8) NaTx; **Panel B** 1) Short KTx; 2) Long KTx; 3) Chloride; 4) β-NaTx; 5) α-NaTx; 6) ICK; 7) DDH; 8) Glycine-rich toxins; 9) Bradykinin Potentiating Peptides; 10) Anionic; 11) Antimicrobial peptide toxins.

**Fig 5:**
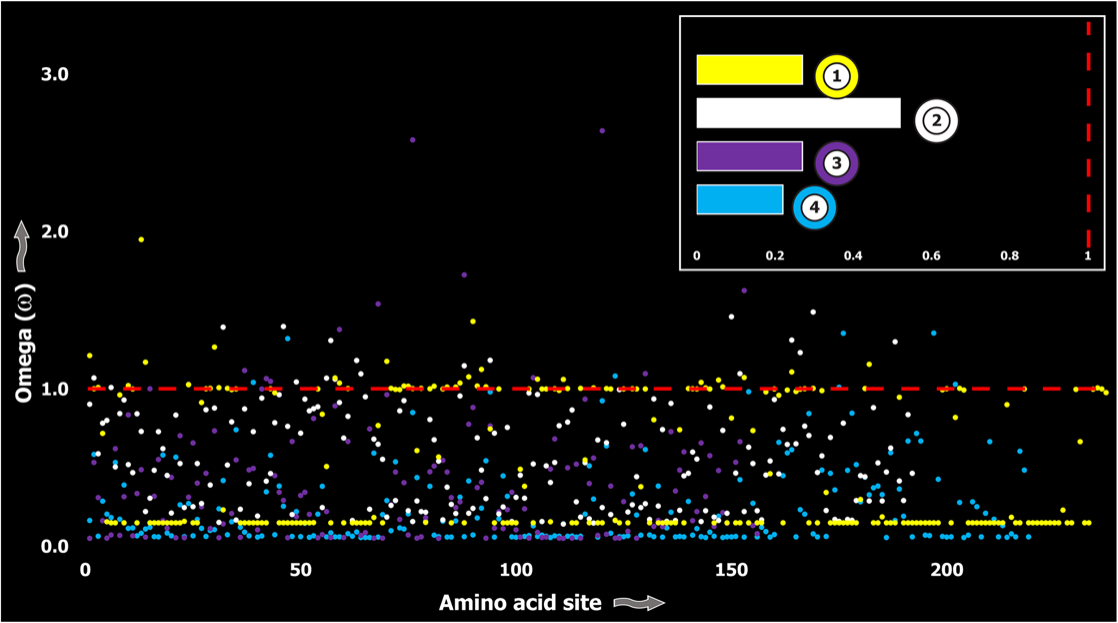
Molecular evolution of venom in coleoids. A plot of site-specific ω against amino acid positions for various coleoid toxin types is presented. The red horizontal line represents the line of neutrality: points above and below this line indicate positive and negative selection, respectively. Bar plot color codes: 1) Serine Protease; 2) PLA_2_; 3) Pacifestin and 4) CAP.

Three (Kallikreins, CRiSPs and crotamines) among the six gene families examined exhibited an evidence for rapid evolution (ω>1 and/or more than 10 positively selected sites), while the remaining were found to be extremely well conserved (S1 Table). Further, we plotted site-wise ω against their respective amino acid position for each of the genes examined. Results indicated that a majority of sites in most venom proteins of ancient lineages evolved under the strong influence of negative selection (Figs 2–5).

**Fig 6:**
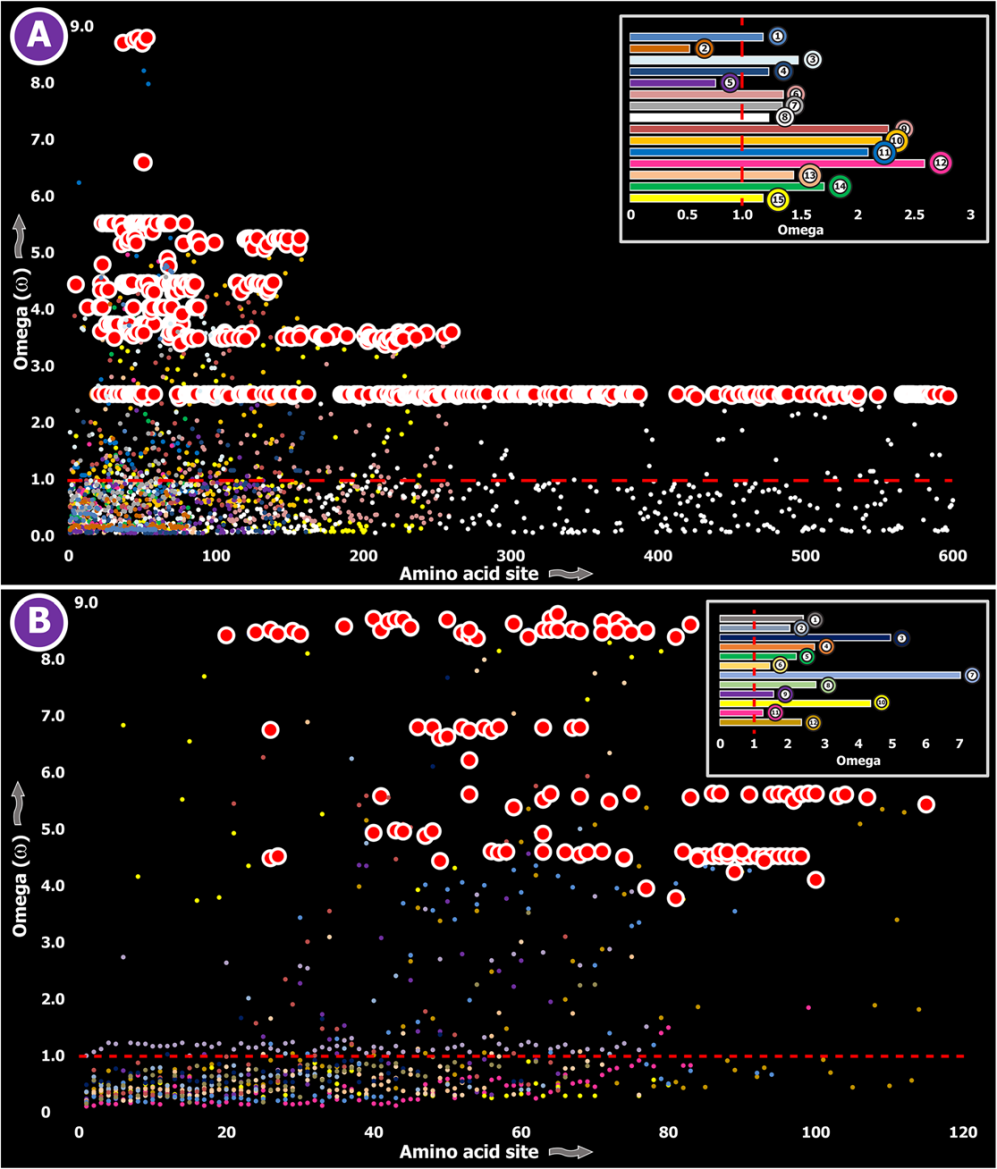
Molecular evolution of venom in advanced snakes (A) and cone snails (B). A plot of site-specific ω against amino acid positions for various advanced snake and cone snail venom-encoding genes is presented. Significantly detected positively selected sites (model 8; Bayes Empirical Bayes approach) are presented as large red circles. The red horizontal line represents the line of neutrality: points above and below this line indicate positive and negative selection, respectively. A corresponding bar plot is provided, which shows the computed ω value for the respective toxin class. Bar-plot color code: **Panel A** 1) β-defensins; 2) Cytotoxins; 3) PII-Disintegrins; 4) Group I PLA_2_s; 5) Group II PLA_2_s; 6) Kallikreins; 7) *Psammophis* SVMPs; 8) Advanced snake SVMPs; 9) Serine Proteases; 10) Lectins; 11) κ-3FTxs; 12) Type III α-neurotoxins; 13) Type II α-neurotoxins; Type I α-neurotoxins; and 14) CRISPs. **Panel B** *Conus marmoreus*—> 1) Superfamily M; 2) Superfamily I2; 3) Superfamily T; 4) Superfamily O2; *C*. *geographus*—> 5) Superfamily O1; 6) Superfamily O2; 7) Superfamily O1; 8) Superfamily M; 9) Superfamily A; 10) Conkunitzin; 11) Conantokin; 12) Con-ikot-ikot.

**Fig 7:**
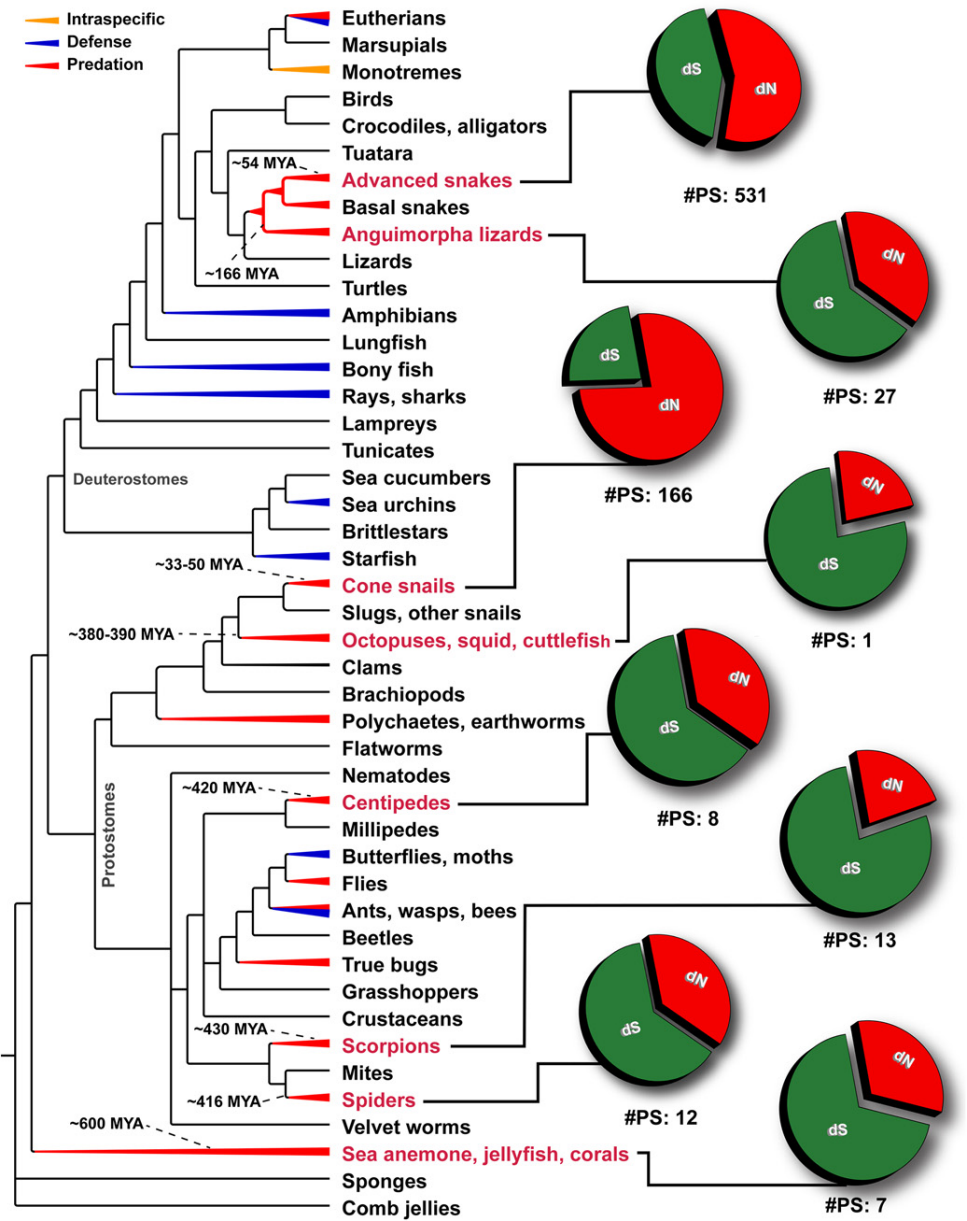
Schematic tree of life depicts the evolution of venom in animals, where blue, red and orange colored lines represent lineages that utilize venom for defense, predation or intraspecific needs, respectively. Divergence times of lineages examined in this study (labels indicated in red) have been indicated. Pie charts depict the proportion of synonymous (dS), non-synonymous (dN) mutations and the number of significantly detected positively selected sites (#PS) in the venom-encoding genes of the respective lineage. Depicted phylogenetic relationships are based on Ref. [13].

In contrast, a large proportion of sites in toxins of evolutionarily young lineages rapidly mutated under the significant influence of positive Darwinian selection (Fig 6). Thus, a stark difference was found in the evolutionary regimes of ancient and evolutionarily young lineages (Fig 7).

Using the mixed effects model of evolution (MEME) several sites that experienced short periods of diversifying selection were also identified in all the examined venomous clades, which indicated that certain sites in these toxin proteins undergo episodic adaptation (Tables 1–3; S1 Table). Considering the long evolutionary histories of these toxin types, we tested for nucleotide substitution saturation (see methods). These tests did not detect saturation in any of the examined datasets (S2 Table).

### The influence of the length of a toxin on its omega

We performed regression analyses to evaluate the possibility of the length of the toxin determining its rate of evolution by plotting ω values for various toxin types against their respective lengths (S1 Fig). The coefficient of determination (*r* ^2^) for toxin types in each of the examined lineages suggested an absence of correlation between the length of the toxin and its ω value, indicating that venom proteins have undergone rapid evolution or extreme sequence conservation irrespective of their size. While most conotoxins are of relatively shorter lengths, snake venom components such as three-finger toxins (3FTxs) and Snake Venom Metalloproteinases (SVMPs) are characterized by lengths of 80 and 600 amino acids, respectively. Despite such stark size differences, these toxins evolved rapidly. Similarly, several venom components in ancient lineages were characterized by a range of lengths. For example, most sea anemone and scorpion neurotoxins were of relatively shorter lengths (40–60 amino acids), while several pore-forming toxins were 450–550 amino acids long. Yet, these toxins were found to have evolved extremely slowly under the influence of purifying selection. Our results thus indicated that the stark differences in ω values for venom proteins of ancient and evolutionarily younger lineages did not result from the differences in size.

## Discussion

### The significance of purifying selection in the evolution venom

Animal venoms are assumed to rapidly diversify under the unabated influence of positive Darwinian selection. They have been theorized to undergo a chemical arms race with prey animals, where the evolution of venom resistance in prey and the invention of efficient toxins in the predatory venomous animal exert reciprocal selection pressure [64], as postulated in the Red Queen hypothesis of Van Valen [65]. While the influence of positive selection is widely recognized, the role of purifying selection in shaping animal venoms has rarely been considered. Investigation of a large number of toxin-encoding gene families in this study has revealed a significant influence of negative selection on venom. Whilst positive selection increases the diversity of venom proteins, purifying selection probably aids in preserving the potency of the venom by filtering out mutations that negatively affect toxin efficiency. However, rare mutations that increase the potency of the venom arsenal (e.g., evolution of novel biochemical activity or increased binding efficiency) are likely to be propagated and preserved in the population. In the absence of a conservatory evolutionary force, neutral or positive selection could modify key residues and result in the reduction of potency or, for worse, the complete loss of bioactivity, which could severely decrease the fitness of the animal. Thus, purifying selection pressure appears to be vital for sustaining the potency and, consequently, shaping the animal venom arsenal.

### Certain toxins are more constrained than others

It has been recently demonstrated that PFTs in Cnidaria, which bind to cell membranes and punch holes, evolve under the heavy constraints of negative selection [26, 66]. The lack of variation in this group of toxins, which includes several unrelated toxin types (e.g., aerolysin-related toxins in sea anemones, independently recruited hydralysins in hydroids, actinoporins and jellyfish toxins), was theorized to be a result of their complex multi-subunit packaging [67] and their ability to attack highly conserved molecular targets, such as cell membranes [26]. Toxins that undergo oligomerization in other classes of animals have also been noted to evolve relatively slowly as a result of structural constraints like the need to conserve sites involved in subunit interaction. While most 3FTxs in snake venoms diversify rapidly, κ-3FTxs, which undergo dimerization, were found to accumulate relatively fewer variations [59]. Similarly, toxins that may function in a ‘non-specific’ manner may also experience negative selection. Here, non-specificity of action is defined as the ability to target regions in a structural/biochemical property dependent (e.g., surface electrostatic charge) and target motif independent manner. For example, cytotoxic 3FTxs and β-defensin toxins—two very potent snake venom proteins, induce cytotoxicity by non-specifically binding to negatively charged cell membranes using hydrophobicity [68] and positively charged molecular surface [69], respectively. As a result, unlike most snake venom components, these proteins remain evolutionarily constrained [59, 62]. Similarly, scorpion lipolytic toxins were also theorized to be evolutionarily constrained because of their non-specific mechanism of action [58]. We found that β-PFTs in centipede venoms, which are similar to the aerolysin-like toxins, evolve under the significant influence of negative selection (Table 1). The lack of variation in this group of toxins may suggest that they either undergo oligomerization like their aerolysin homologues in other lineages or the possibility that they may employ a non-specific mechanism of action. A plot of site-specific ω against their respective amino acid positions reveals the extreme conservation of such toxin types that employ unique strategies for causing toxicity in envenomed animals (S2 Fig). As it allows the targeting of a wide variety of animals, the strategy of exerting toxic action non-specifically or by targeting highly conserved molecular sites, appears to be advantageous and follows a contrastingly different evolutionary regime in comparison to toxins that specialize in attacking highly plastic molecular receptors.

### The rise and fall of venom evolution

A comparison of evolutionary regimes of ancient and evolutionarily younger lineages suggests a fascinating strategy of venom evolution. When venomous animals venture into novel ecological niches, they encounter new types of prey and predatory animals. Consequently, in order to adapt and conquer niches, they would need to fine-tune venom proteins to efficiently target these new animals. Several sites detected as episodically adaptive—i.e., sites that experience short bursts of adaptive selection, in these ancient clades may be reflective of such shifts in ecology. We propose that these earlier periods in the evolutionary history of a venomous species are accompanied by the significant influence of diversifying selection on the venom arsenal, which would expand the range of target sites and/or result in the origination of novel biochemical activities. This is particularly advantageous, since novel toxins generated may facilitate the efficient and rapid incapacitation of newly encountered prey and predatory animals. The period of expansion is followed by longer periods of purification, where the significant influence of negative selection preserves the potency of the toxin. Whenever there is a major shift in ecology or environment, the aforementioned stages of evolution repeat. Thus, we propose that venom-encoding genes mostly employ a ‘two-speed’ mode of evolution, where episodic diversifying selection accompanies the earlier stages of ecological specialization (e.g., diet and range expansion), resulting in the rapid diversification of the venom arsenal, followed by a longer period of purification and fixation that ensure the sustainability of venom potency. The low sequence variation in venom-encoding genes of ancient clades could be reflective of such long periods of purification and fine-tuning. In contrast, advanced snakes and cone snails, being evolutionarily very young, could still be undergoing the period of expansion and, consequently, exhibit a pronounced signature of positive Darwinian selection.

However, it should be noted that the ‘two-speed’ model of evolution is likely applicable to venoms that serve predominantly predatory roles. Due to limited toxin sequence information from venoms that are employed for non-predatory functions (e.g., intraspecific competition in platypus, exclusively defensive roles in fishes, etc.), it remains to be seen whether they too follow our proposed evolutionary model.

To conclude, in addition to unraveling the evolutionary regimes of toxin families in centipedes and spiders, which are amongst the first terrestrial venomous lineages, our findings highlight the pivotal roles of purifying and episodic selections in shaping animal venoms. Our findings enabled the postulation of a new theory of venom evolution in the animal kingdom that emphasizes the dynamic nature of these complex biochemical cocktails.

## Methods

### Genome searches

Toxin homologues were identified in the recently published genome of the coastal European centipede *Strigamia maritima* [70] by querying amino acid sequences of each toxin type against all six reading frames using the tblastn tool [71].

### Evolutionary analyses

Translated nucleotide sequences were aligned using MUSCLE 3.8 [72]. The best-fit model of nucleotide substitution for individual toxin datasets was determined according to the Akaike’s information criterion using jModeltest 2.1 [73] and model-averaged parameter estimates were used for the reconstruction of trees. Phylogenetic trees were built using PhyML 3.0 [74], where node support was evaluated with 1,000 bootstrapping replicates. Maximum-likelihood (ML) models [75] implemented in Codeml of the PAML package [76] were utilized to identify the influence of natural selection on toxin families[6]. As no *a priori* expectation exists, we compared likelihood values for a pair of models with different assumed ω distributions: M7 (β) versus M8 (β and ω) [77]. Only when the alternate model (M8) shows a better fit than the null model (M7) in the likelihood ratio test (LRT), are its results considered significant. LRT is estimated as twice the difference in ML values between the nested models, and is compared with the *χ* ^2^ distribution with the appropriate degree of freedom—the difference in the number of parameters of the two models. Further, we used the Bayes empirical Bayes (BEB) approach [78] in M8 to detect amino acids under positive selection by calculating the posterior probability (PP) that a particular site belongs to a given selection class (neutral, conserved, or highly variable). Sites with PP ≥ 95% of belonging to the ‘‘ω > 1 class” are inferred to be positively selected. HyPhy’s [79] FUBAR approach [80] was used to detect sites evolving under pervasive diversifying and purifying selection pressures. MEME [81] was also employed to identify episodically diversifying sites. Sequence alignments used for selection assessments have been made available as a zipped file (S1 File; see S3 Table for accession list). Nucleotide substitution saturation was tested using DAMBE 5.5.9 [82] using the recommended protocol [83].

## Supporting Information

S1 Fig Regression analyses of various toxin types in each of the examined venomous lineage. Omega values are plotted against the size/length of the respective toxin type. The coefficient of determination (*r* ^2^) for each clade has also presented. *r* ^2^ values closer to 0 suggest an absence of correlation between the size of the toxin and its omega (rate of evolution) value.
(PDF)

S2 Fig Certain toxins are more constrained than others. A plot of site-specific w against amino acid positions for various toxin types is presented. Significantly detected positively selected sites (model 8; Bayes Empirical Bayes approach) are presented as large red circles. The red horizontal line represents the line of neutrality: points above and below this line indicate positive and negative selection, respectively.
(PDF)

S1 Table Evolution of venom in ancient and evolutionarily young animal lineages. The table provides the results of the PAML and MEM tests for the various toxin families.
(PDF)

S2 Table Nucleotide substitution saturation tests. The Iss and Iss.c sym values for each toxin family are provided in the table.
(PDF)

S3 Table List of the Genbank accession numbers of the sequences analyzed in this study. (PDF)

S1 File ZIP file containing all the alignments used in this study. The alignments are provided as zipped Fasta files.
(ZIP)
