# Supplementary material for: The Rise and Fall of an Evolutionary Innovation: Contrasting Strategies of Venom Evolution in Ancient and Young Animals
Source: PLoS Genet. 2015 Oct 22;11(10):e1005596. doi: 10.1371/journal.pgen.1005596 (PMC4619613; doi:10.1371/journal.pgen.1005596)
Supplement: S1 Fig — Omega values are plotted against the size/length of the respective toxin type. The coefficient of determination (r 2) for each clade has also presented. r 2 values closer to 0 suggest an absence of correlation between the size of the toxin and its omega (rate of evolution) value. (PDF) [file pgen.1005596.s001.pdf]

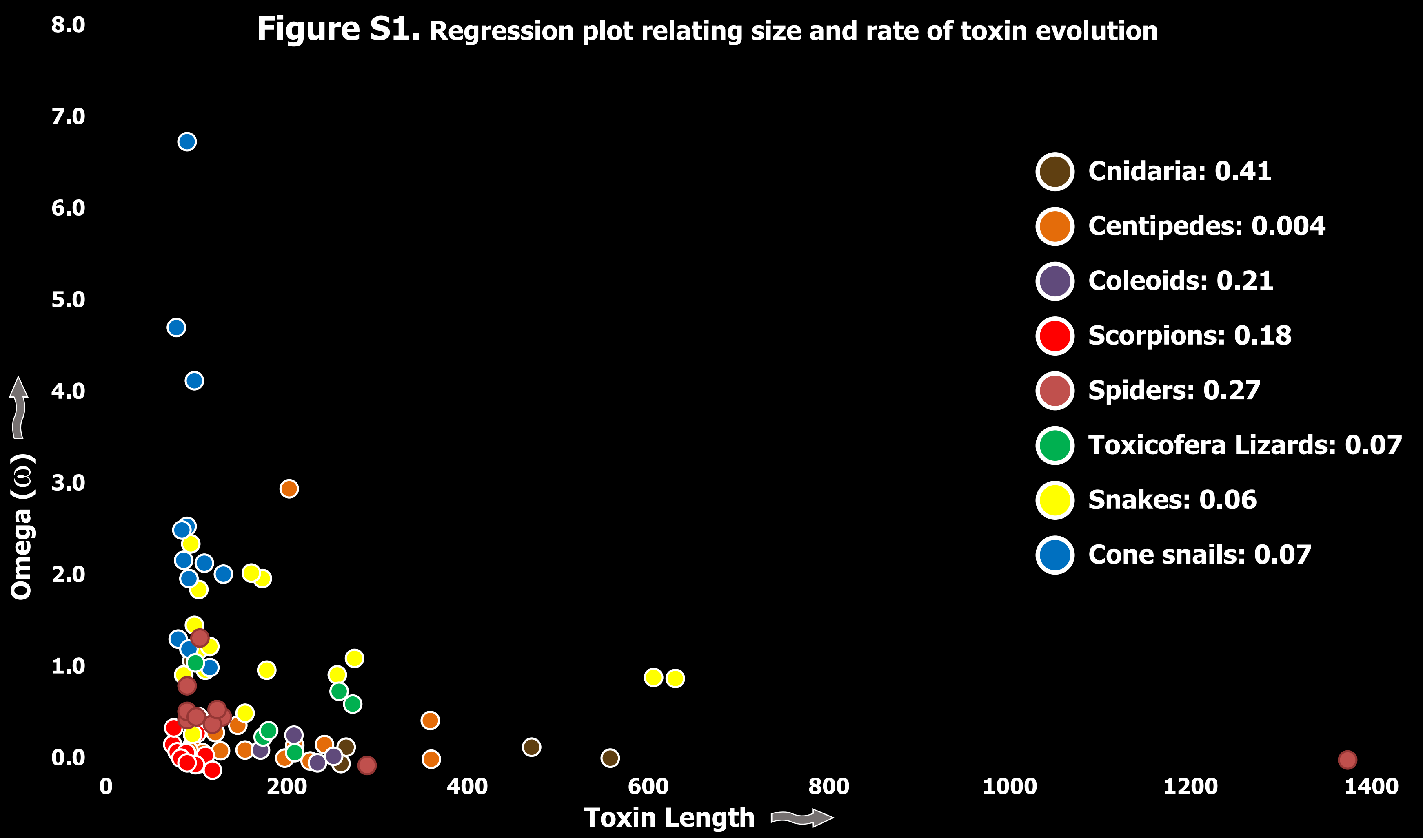

Figure S1: Regression analyses of various toxin types in each of the examined venomous lineage is presented here. Omega values are plotted against the size/length of the respective toxin type. The coefficient of determination (r2) for each clade has also presented. r2 values closer to 0 suggest an absence of correlation between the size of the toxin and its omega (rate of evolution) value.
