# Supplementary material for: The Rise and Fall of an Evolutionary Innovation: Contrasting Strategies of Venom Evolution in Ancient and Young Animals
Source: PLoS Genet. 2015 Oct 22;11(10):e1005596. doi: 10.1371/journal.pgen.1005596 (PMC4619613; doi:10.1371/journal.pgen.1005596)
Supplement: S2 Fig — A plot of site-specific w against amino acid positions for various toxin types is presented. Significantly detected positively selected sites (model 8; Bayes Empirical Bayes approach) are presented as large red circles. The red horizontal line represents the line of neutrality: points above and below this line indicate positive and negative selection, respectively. (PDF) [file pgen.1005596.s002.pdf]

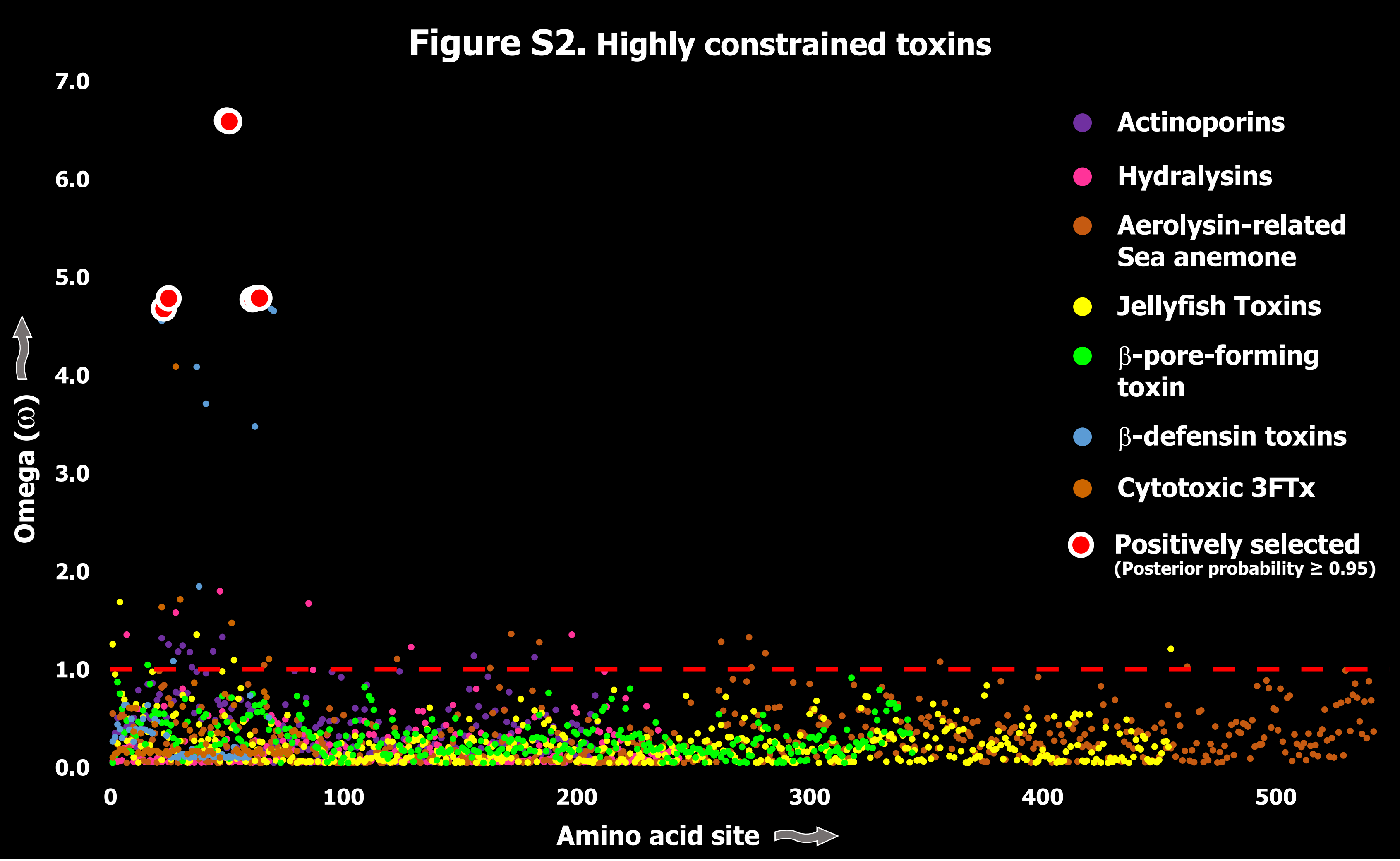

Figure S2: Certain toxins are more constrained than others. A plot of site-specific ω against amino acid positions for various toxin types is presented. Significantly detected positively selected sites (model 8; Bayes Empirical Bayes approach) are presented as large red circles. The red horizontal line represents the line of neutrality: points above and below this line indicate positive and negative selection, respectively.
