# Supplementary material for: The Rise and Fall of an Evolutionary Innovation: Contrasting Strategies of Venom Evolution in Ancient and Young Animals
Source: PLoS Genet. 2015 Oct 22;11(10):e1005596. doi: 10.1371/journal.pgen.1005596 (PMC4619613; doi:10.1371/journal.pgen.1005596)
Supplement: S1 Table — The table provides the results of the PAML and MEM tests for the various toxin families. (PDF) [file pgen.1005596.s003.pdf]

Table S1. Evolution of venom in ancient and evolutionarily young animal lineages

	PAML	MEME	Reference
**Cnidaria**			
**Type I and Type II sodium channel toxins**	**PS:** 0<br>**ω:** 0.58	8	(Jouiaei, et al. 2015)
**Type I potassium channel toxins**	**PS:** 1<br>0.66	5	
**Type III potassium channel toxins**	**PS:** 6<br>**ω:** 1.33	5	
**Actinoporins**	**PS:** 0<br>**ω:** 0.39	25	
**Sea Anemone aerolyin-related toxins**	**PS:** 0<br>**ω:** 0.27	41	
**Hydralysins**	**PS:** 0<br>**ω:** 0.21	9	
**Jellyfish toxins**	**PS:** 0<br>**ω:** 0.39	17	
**Small cysteine-rich proteins (SCRiPs) in corals**	**PS:** 0<br>**ω:** 0.72	7	
**Coleoids**			
**Cysteine-rich secretory proteins, antigen 5, and pathogenesis-related 1 (CAP)**	**PS:** 0<br>**ω:** 0.22	1	(Ruder, et al. 2013)
**Phospholipase A2 (PLA2)**	**PS:** 0<br>**ω:** 0.52	1	
**Pacifastin**	**PS:** 0<br>**ω:** 0.36	3	
**Serine proteases**	**PS:** 1<br>**ω:** 0.29	26	
**Scorpions**			
**α-sodium channel toxins**	**PS:** 5<br>**ω:** 0.54	19	(Sunagar, et al. 2013c)
**β-sodium channel toxins**	**PS:** 4<br>**ω:** 0.53	18	
**Long potassium channel toxins**	**PS:** 1<br>**ω:** 0.30	10	
**Short potassium channel toxins**	**PS:** 0<br>**ω:** 0.42	4	
**Chloride channel toxins**	**PS:** 2<br>**ω:** 0.6	5	
**Inhibitor cystine knot (ICK)**	**PS:** 0	0	

	**ω:** 0.34		
**Disulphide-directed beta-hairpin (DDH)**	**PS:** 0	1	
	**ω:** 0.32		
**Antimicrobial peptides (AMP)**	**PS:** 1	2	
	**ω:** 0.33		
**Linear peptides**	**PS:** 0	3	
	**ω:** 0.27		
**Bradykinin**	**PS:** 0	2	
	**ω:** 0.20		
**Anionic peptides**	**PS:** 0	0	
	**ω:** 0.22		
**Glycine-rich peptides**	**PS:** 0	1	
	**ω:** 0.14		
**Spiders**			
**Family E ICK**	**PS:** 3	6	
	**ω:** 0.64		
**Funnel-web spider ω toxins**	**PS:** 0	7	
	**ω:** 0.69		
**Funnel-web spider κ toxins**	**PS:** 0	0	(Pineda, et al. 2014)
	**ω:** 1.06		
**Funnel-web spider ω/κ hexatoxins**	**PS:** 1	8	
	**ω:** 0.78		
**Tarantula Huwentoxin-1 Family**	**PS:** 1	19	
	**ω:** 0.72		
***Loxosceles* Sphingomyelinase D**	**PS:** 0	22	
	**ω:** 0.19		
**Kunitz-type Serine Protease Inhibitors**	**PS:** 4	2	
	**ω:** 1.58		
**Magi-1 Family toxins**	**PS:** 0	17	
	**ω:** 0.72		
**α-Latrotoxins**	**PS:** 3	47	
	**ω:** 0.25		
**U1-lycotoxin family**	**PS:** 0	2	
	**ω:** 0.80		
**Toxicofera Lizards**			
**Kallikreins**	**PS:** 14	30	
	**ω:** 0.86		
**CRiSPs**	**PS:** 13	11	(Sunagar, et al. 2012)
	**ω:** 1.0		
**Crotamine**	**PS:** 0	1	
	**ω:** 1.31		

**Phospholipase A2**	**PS:** 0	2	
	**ω:** 0.50		
**Natriuretic peptides**	**PS:** 0	3	
	**ω:** 0.57		
**Nerve Growth Factor**	**PS:** 0	1	(Sunagar, et al. 2013a)
	**ω:** 0.33		

	PAML	MEME	Reference
**Advanced Snakes**			
**Cysteine-rich Secretory Proteins (CRISPs)**	**PS:** 35	35	(Sunagar, et al. 2012)
	**ω:** 1.18		
**Group I Phospholipase A_2**	**PS:** 49	55	
	**ω:** 1.23		
**Group II Phospholipase A_2**	**PS:** 14	39	
	**ω:** 0.76		
**Type I α-neurotoxins**	**PS:** 19	13	(Sunagar, et al. 2013b)
	**ω:** 1.72		
**Type II α-neurotoxins**	**PS:** 21	30	
	**ω:** 1.45		
**Type III α-neurotoxins**	**PS:** 30	24	
	**ω:** 2.61		
**Cytotoxins**	**PS:** 2	0	
	**ω:** 0.53		
**κ-neurotoxins**	**PS:** 5	2	
	**ω:** 2.11		
**Snake venom metalloproteinases (SVMPs)**	**PS:** 160	168	File S1
	**ω:** 1.14		
**SVMPs in *Psammophis mossambicus***	**PS:** 2	1	(Brust, et al. 2013)
	**ω:** 1.23		
**SVMPs in *Echis coloratus***	**PS:** 34	28	
	**ω:** 1.15		
**PII Disintegrins**	**PS:** 14	9	File S1 and (Juarez, et al. 2008)
	**ω:** 1.49		
**Crotamines**	**PS:** 11	4	(Sunagar, et al. 2014)
	**ω:** 1.18		
**Kallikreins**	**PS:** 36	45	
	**ω:** 1.36		
**Lectins**	**PS:** 48	51	
	**ω:** 1.29		
**Serine Proteases**	**PS:** 51	69	

	**ω:** 1.18		
**Cone snails (Dutertre, et al. 2014)**			
**Con-ikot-ikot in *Conus geographus***	**PS:** 23	7	(Dutertre, et al. 2014)
	**ω:** 2.38		
**Conantokin in *C. geographus***	**PS:** 17	31	
	**ω:** 1.26		
**Conkunitzin in *C. geographus***	**PS:** 26	0	
	**ω:** 4.39		
**Superfamily A in *C. geographus***	**PS:** 6	5	
	**ω:** 1.57		
**Superfamily M in *C. geographus***	**PS:** 14	6	
	**ω:** 2.80		
**Superfamily O1 in *C. geographus***	**PS:** 25	4	
	**ω:** 7.0		
**Superfamily O2 in *C. geographus***	**PS:** 0	0	
	**ω:** 1.46		
**Superfamily O1 in *C. marmoreus***	**PS:** 9	6	
	**ω:** 2.23		
**Superfamily O2 in *C. marmoreus***	**PS:** 13	4	
	**ω:** 2.76		
**Superfamily T in *C. marmoreus***	**PS:** 7	1	
	**ω:** 4.97		
**Superfamily I2 in *C. marmoreus***	**PS:** 16	6	
	**ω:** 2.40		
**Superfamily M in *C. marmoreus***	**PS:** 10		
	**ω:** 2.43	4	

Note: For details regarding datasets and analyses, please see the cited papers where they were reported. Results of analyses conducted in this study are colored in green.

Legend:
PS: Positively selected sites detected by the Bayes Empirical Bayes approach implemented in model 8 of PAML.
MEME: episodically diversifying sites identified by the mixed effects model of evolution
ω: mean dN/dS

Table S1 References

Brust A, Sunagar K, Undheim EA, Vetter I, Yang DC, Casewell NR, Jackson TN, Koludarov I, Alewood PF, Hodgson WC, et al. 2013. Differential evolution and neofunctionalization of snake venom metalloprotease domains. Mol Cell Proteomics 12:651-663.

Dutertre S, Jin AH, Vetter I, Hamilton B, Sunagar K, Lavergne V, Dutertre V, Fry BG, Antunes A, Venter DJ, et al. 2014. Evolution of separate predation- and defence-evoked venoms in carnivorous cone snails. Nat Commun 5:3521.

Jouiaei M, Sunagar K, Federman Gross A, Scheib H, Alewood PF, Moran Y, Fry BG. 2015. Evolution of an ancient venom: recognition of a novel family of cnidarian toxins and the common evolutionary origin of sodium and potassium neurotoxins in sea anemone. Mol Biol Evol 32:1598-1610.

Juarez P, Comas I, Gonzalez-Candelas F, Calvete JJ. 2008. Evolution of snake venom disintegrins by positive Darwinian selection. Mol Biol Evol 25:2391-2407.

Pineda SS, Sollod BL, Wilson D, Darling A, Sunagar K, Undheim EA, Kely L, Antunes A, Fry BG, King GF. 2014. Diversification of a single ancestral gene into a successful toxin superfamily in highly venomous Australian funnel-web spiders. BMC Genomics 15:177.

Ruder T, Sunagar K, Undheim EA, Ali SA, Wai TC, Low DH, Jackson TN, King GF, Antunes A, Fry BG. 2013. Molecular phylogeny and evolution of the proteins encoded by coleoid (cuttlefish, octopus, and squid) posterior venom glands. J Mol Evol 76:192-204.

Sunagar K, Fry BG, Jackson TN, Casewell NR, Undheim EA, Vidal N, Ali SA, King GF, Vasudevan K, Vasconcelos V, et al. 2013a. Molecular evolution of vertebrate neurotrophins: co-option of the highly conserved nerve growth factor gene into the advanced snake venom arsenalf. PLoS One 8:e81827.

Sunagar K, Jackson TN, Undheim EA, Ali SA, Antunes A, Fry BG. 2013b. Three-fingered RAVERs: Rapid Accumulation of Variations in Exposed Residues of snake venom toxins. Toxins (Basel) 5:2172-2208.

Sunagar K, Johnson WE, O'Brien SJ, Vasconcelos V, Antunes A. 2012. Evolution of CRISPs associated with toxicoferan-reptilian venom and mammalian reproduction. Mol Biol Evol 29:1807-1822.

Sunagar K, Undheim EA, Chan AH, Koludarov I, Munoz-Gomez SA, Antunes A, Fry BG. 2013c. Evolution stings: the origin and diversification of scorpion toxin peptide scaffolds. Toxins (Basel) 5:2456-2487.

Sunagar K, Undheim EA, Scheib H, Gren EC, Cochran C, Person CE, Koludarov I, Kelln W, Hayes WK, King GF, et al. 2014. Intraspecific venom variation in the medically significant Southern Pacific Rattlesnake (Crotalus oreganus helleri): biodiscovery, clinical and evolutionary implications. J Proteomics 99:68-83.
