# Supplementary material for: The Rise and Fall of an Evolutionary Innovation: Contrasting Strategies of Venom Evolution in Ancient and Young Animals
Source: PLoS Genet. 2015 Oct 22;11(10):e1005596. doi: 10.1371/journal.pgen.1005596 (PMC4619613; doi:10.1371/journal.pgen.1005596)
Supplement: S2 Table — The Iss and Iss.c sym values for each toxin family are provided in the table. (PDF) [file pgen.1005596.s004.pdf]

Table S2. Nucleotide substitution saturation tests

	Iss	Iss.c sym
**Centipedes**		
β-pore-forming toxins	0.639	0.835
CAP	0.352	1.050
LDLA	0.555	0.778
SLPTX01	0.5854	0.6704
SLPTX04	0.5872	0.6176
SLPTX05	0.6187	0.7141
SLPTX10	0.476	0.786
SLPTX11	0.1304	23.8151
SLPTX12	0.2131	0.7047
SLPTX13	0.4776	0.7150
SLPTX15	0.6047	0.6080
SLPTX16	0.558	0.809
SLPTX17	0.5032	0.7068
Novel family 01	0.2770	0.7391
Novel family 04	0.0219	0.7376
Novel family 06	0.3491	0.7497
Novel family 08	0.3889	0.7488
**Spiders**		
Family E ICK	0.767	0.863
Funnel-web spider ω toxins	0.258	0.806
Funnel-web spider κ toxins	0.0377	0.7478
Funnel-web spider ω/κ hexatoxins	0.330	0.825
Tarantula Huwentoxin-1 Family	0.609	0.941
Loxosceles Sphingomyelinase D	0.442	0.810
Kunitz-type Serine Protease Inhibitors	0.095	0.776
Magi-1 Family toxins	0.122	0.779
α-Latrotoxins	0.175	0.781
U1-lycotoxin family	0.069	0.777
**Cnidaria**		
Actinoporins	0.592	0.778
Hydralysins	0.2762	0.6124
Aerolysin-related (sea anemone)	0.6042	0.7015
Jellyfish toxins	0.6631	0.7575
KTx Type I	0.4868	0.6383
KTx Type III	0.4186	0.6521
NaTx Type I and Type II	0.453	0.793
SCRiPs	0.6126	0.4176
**Scorpions**		

α-Na_V-CSα/β	0.435	0.779
β-Na_V-CSα/β	0.516	0.872
Long-K_V-CSα/β	0.579	0.804
Short-K_V-CSα/β	0.571	0.986
Cl_V-CSα/β	0.3731	0.4560
ICK	0.4698	0.7211
DDH	0.2686	0.7583
AMP	0.535	0.797
Linear	0.576	0.895
Bradykinin Potentiating Peptides	0.4877	0.6394
Anionic	0.3341	0.4729
Glycine-rich	0.4978	0.7482

Legend
ISS: index of substitution saturation
ISS.c sym: critical value assuming a symmetrical topology
ISS < ISS.c: Absence of significance saturation.
