# Supplementary material for: The Rise and Fall of an Evolutionary Innovation: Contrasting Strategies of Venom Evolution in Ancient and Young Animals
Source: PLoS Genet. 2015 Oct 22;11(10):e1005596. doi: 10.1371/journal.pgen.1005596 (PMC4619613; doi:10.1371/journal.pgen.1005596)
Supplement: S3 Table — (PDF) [file pgen.1005596.s005.pdf]

Table S3: Sequences Analyzed

Centipedes

β-PFT		
1	KC144049.1	*Scolopendra subspinipes dehaani*
2	KC144990.1	*Scolopendra subspinipes dehaani*
3	KC145028.1	*Scolopendra subspinipes dehaani*
4	GASH01000017	*Scolopendra morsitans*
5	GASH01000021	*Scolopendra morsitans*
6	GASL01000003	*Cormocephalus westwoodi*
7	GASI01000025	*Ethmostigmus rubripes*
8	GASH01000019	*Scolopendra morsitans*
9	GASH01000020	*Scolopendra morsitans*
10	GASI01000018	*Ethmostigmus rubripes*
11	GASI01000019	*Ethmostigmus rubripes*
12	GASI01000020	*Ethmostigmus rubripes*
13	GASI01000022	*Ethmostigmus rubripes*
14	GASI01000021	*Ethmostigmus rubripes*
15	GASI01000023	*Ethmostigmus rubripes*
16	GASI01000024	*Ethmostigmus rubripes*
17	KC144624.1	*Scolopendra subspinipes dehaani*
18	KC144623.1	*Scolopendra subspinipes dehaani*
19	KC144831.1	*Scolopendra subspinipes dehaani*
20	GASK01000007	*Scolopendra alternans*
21	GASH01000034	*Scolopendra morsitans*
22	KC144026.1	*Scolopendra subspinipes dehaani*
23	GASH01000018	*Scolopendra morsitans*
24	GASL01000011	*Cormocephalus westwoodi*
25	GASH01000035	*Scolopendra morsitans*
26	GASL01000012	*Cormocephalus westwoodi*
27	GASI01000012	*Ethmostigmus rubripes*
28	GASI01000016	*Ethmostigmus rubripes*
29	GASI01000014	*Ethmostigmus rubripes*
30	GASI01000015	*Ethmostigmus rubripes*
31	GASI01000013	*Ethmostigmus rubripes*
32	GASI01000017	*Ethmostigmus rubripes*
33	GASK01000006	*Scolopendra alternans*
34	GASR01000015	*Thereuopoda longicornis*

Table S3: Sequences Analyzed

35	GASR01000059	*Thereuopoda longicornis*
36	GASR01000070	*Thereuopoda longicornis*
37	GASR01000077	*Thereuopoda longicornis*
38	SMAR004243RA	*Strigamia maritima*
39	SMAR004242RA	*Strigamia maritima*
40	SMAR012417RA	*Strigamia maritima*
41	JZ574184.1	*Scolopendra viridis*

**CAP**		
1	KC144027.1	*Scolopendra subspinipes dehaani*
2	KC144855.1	*Scolopendra subspinipes dehaani*
3	KC144397.1	*Scolopendra subspinipes dehaani*
4	KC144210.1	*Scolopendra subspinipes dehaani*
5	KC144762.1	*Scolopendra subspinipes dehaani*
6	KC144967.1	*Scolopendra subspinipes dehaani*
7	KC144970.1	*Scolopendra subspinipes dehaani*
8	KC144362.1	*Scolopendra subspinipes dehaani*
9	KC144334.1	*Scolopendra subspinipes dehaani*
10	KC145115.1	*Scolopendra subspinipes dehaani*
11	KC145116.1	*Scolopendra subspinipes dehaani*
12	KC145031.1	*Scolopendra subspinipes dehaani*
13	KC144888.1	*Scolopendra subspinipes dehaani*
14	KC145067.1	*Scolopendra subspinipes dehaani*
15	KC145069.1	*Scolopendra subspinipes dehaani*
16	KC145077.1	*Scolopendra subspinipes dehaani*
17	KC145076.1	*Scolopendra subspinipes dehaani*
18	KC144555.1	*Scolopendra subspinipes dehaani*
19	KC144414.1	*Scolopendra subspinipes dehaani*
20	KC144549.1	*Scolopendra subspinipes dehaani*
21	KC144061.1	*Scolopendra subspinipes dehaani*
22	KC144605.1	*Scolopendra subspinipes dehaani*
23	KC144960.1	*Scolopendra subspinipes dehaani*
24	GASL01000013	*Cormocephalus westwoodi*
25	GASI01000039	*Ethmostigmus rubripes*
26	GASI01000040	*Ethmostigmus rubripes*
27	GASH01000046	*Scolopendra morsitans*
28	GASH01000047	*Scolopendra morsitans*

Table S3: Sequences Analyzed

29	GASH01000048	*Scolopendra morsitans*
30	GASH01000042	*Scolopendra morsitans*
31	GASH01000043	*Scolopendra morsitans*
32	GASH01000044	*Scolopendra morsitans*
33	GASK01000008	*Scolopendra alternans*
34	GASK01000009	*Scolopendra alternans*
35	GASK01000010	*Scolopendra alternans*
36	GASH01000049	*Scolopendra morsitans*
37	GASH01000045	*Scolopendra morsitans*
38	GASH01000050	*Scolopendra morsitans*
39	GASH01000036	*Scolopendra morsitans*
40	GASH01000037	*Scolopendra morsitans*
41	GASH01000038	*Scolopendra morsitans*
42	GASH01000039	*Scolopendra morsitans*
43	GASH01000040	*Scolopendra morsitans*
44	GASH01000041	*Scolopendra morsitans*
45	GASL01000014	*Cormocephalus westwoodi*
46	GASL01000015	*Cormocephalus westwoodi*
47	GASL01000016	*Cormocephalus westwoodi*
48	GASI01000038	*Ethmostigmus rubripes*
49	GASI01000035	*Ethmostigmus rubripes*
50	GASI01000036	*Ethmostigmus rubripes*
51	GASI01000037	*Ethmostigmus rubripes*
52	GASR01000079	*Thereuopoda longicornis*
53	GASR01000081	*Thereuopoda longicornis*
54	GASR01000082	*Thereuopoda longicornis*
55	GASR01000080	*Thereuopoda longicornis*
56	GASH01000051	*Scolopendra morsitans*
57	GASH01000053	*Scolopendra morsitans*
58	GASH01000052	*Scolopendra morsitans*
59	SMAR001064	*Strigamia maritima*
60	SMAR001135	*Strigamia maritima*
61	SMAR012541	*Strigamia maritima*
62	SMAR002427	*Strigamia maritima*
63	SMAR001362	*Strigamia maritima*
64	SMAR010832	*Strigamia maritima*
65	SMAR001361	*Strigamia maritima*

Table S3: Sequences Analyzed

66	SMAR014499	*Strigamia maritima*
67	SMAR002664	*Strigamia maritima*

**LDLA**		
1	GASI01000061.1	*Ethmostigmus rubripes*
2	KC144921.1	*Scolopendra subspinipes dehaani*
3	KC144150.1	*Scolopendra subspinipes dehaani*
4	KC144292.1	*Scolopendra subspinipes dehaani*
5	KC145078.1	*Scolopendra subspinipes dehaani*
6	KC144091.1	*Scolopendra subspinipes dehaani*
7	GASL01000024.1	*Cormocephalus westwoodi*
8	GASK01000016.1	*Scolopendra alternans*
9	GASI01000074.1	*Ethmostigmus rubripes*
11	GASI01000060.1	*Ethmostigmus rubripes*
12	GASR01000087.1	*Thereuopoda longicornis*
13	GASI01000068.1	*Ethmostigmus rubripes*
14	GASI01000071.1	*Ethmostigmus rubripes*
15	GASH01000067.1	*Scolopendra morsitans*
17	GASH01000070.1	*Scolopendra morsitans*
18	GASK01000017.1	*Scolopendra alternans*
19	GASH01000068.1	*Scolopendra morsitans*
20	GASI01000072.1	*Ethmostigmus rubripes*
21	GASI01000073.1	*Ethmostigmus rubripes*
22	GASI01000069.1	*Ethmostigmus rubripes*
23	GASI01000070.1	*Ethmostigmus rubripes*
24	GASR01000088.1	*Thereuopoda longicornis*
25	GASI01000075.1	*Ethmostigmus rubripes*
26	GASI01000076.1	*Ethmostigmus rubripes*
27	GASI01000057.1	*Ethmostigmus rubripes*
28	GASI01000058.1	*Ethmostigmus rubripes*
29	GASI01000059.1	*Ethmostigmus rubripes*
30	GASH01000069.1	*Scolopendra morsitans*
31	KC144567.1	*Scolopendra subspinipes dehaani*
32	GASH01000072.1	*Scolopendra morsitans*
33	GASH01000073.1	*Scolopendra morsitans*
34	GASH01000074.1	*Scolopendra morsitans*
35	KC144374.1	*Scolopendra subspinipes dehaani*

Table S3: Sequences Analyzed

36	KC144795.1	*Scolopendra subspinipes dehaani*
37	GASL01000025.1	*Cormocephalus westwoodi*
38	GASL01000026.1	*Cormocephalus westwoodi*
39	GASL01000027.1	*Cormocephalus westwoodi*
40	GASL01000028.1	*Cormocephalus westwoodi*
41	GASK01000019.1	*Scolopendra alternans*
42	KC144318.1	*Scolopendra subspinipes dehaani*
43	KC144050.1	*Scolopendra subspinipes dehaani*
44	GASI01000062.1	*Ethmostigmus rubripes*
45	GASI01000063.1	*Ethmostigmus rubripes*
46	GASI01000066.1	*Ethmostigmus rubripes*
47	GASI01000064.1	*Ethmostigmus rubripes*
48	GASI01000065.1	*Ethmostigmus rubripes*
49	KC144936.1	*Scolopendra subspinipes dehaani*
50	GASH01000071.1	*Scolopendra morsitans*
53	SMAR004525	*Strigamia maritima*
54	JZ574180.1	*Scolopendra viridis*

**Novel Family 1**		
1	GASI01000001.1	*Ethmostigmus rubripes*
2	GASI01000003.1	*Ethmostigmus rubripes*
3	GASI01000002.1	*Ethmostigmus rubripes*
4	GASH01000001.1	*Scolopendra morsitans*
5	GASI01000004.1	*Ethmostigmus rubripes*
6	GASK01000001.1	*Scolopendra alternans*
7	GASH01000002.1	*Scolopendra morsitans*

**Novel Family 4**		
1	GASR01000001	*Thereuopoda longicornis*
2	GASR01000003	*Thereuopoda longicornis*
3	GASR01000002	*Thereuopoda longicornis*
4	GASR01000004	*Thereuopoda longicornis*
5	GASR01000007	*Thereuopoda longicornis*
6	GASR01000006	*Thereuopoda longicornis*
7	GASR01000005	*Thereuopoda longicornis*

Table S3: Sequences Analyzed

Novel Family 6		
1	GASI01000008	*Ethmostigmus rubripes*
2	GASI01000009	*Ethmostigmus rubripes*
3	GASI01000010	*Ethmostigmus rubripes*
4	GASJ01000009	*Ethmostigmus rubripes epidermal*
5	GASK01000002	*Scolopendra alternans*
6	GASK01000003	*Scolopendra alternans*
7	GASL01000002	*Cormocephalus westwoodi*

Novel Family 8		
1	GASR01000008	*Thereuopoda longicornis*
2	GASR01000011	*Thereuopoda longicornis*
3	GASR01000012	*Thereuopoda longicornis*
4	GASR01000009	*Thereuopoda longicornis*
5	GASR01000010	*Thereuopoda longicornis*
6	SMAR005835	*Strigamia maritima*
7	SMAR005836	*Strigamia maritima*

Novel Family 11		
1	GASH01000013	*Scolopendra morsitans*
2	GASH01000014	*Scolopendra morsitans*
3	GASH01000015	*Scolopendra morsitans*
4	GASH01000016	*Scolopendra morsitans*
5	GASK01000004	*Scolopendra alternans*

SCUTX02		
1	Thereuopoda longicornis	*GASR01000101*
2	Thereuopoda longicornis	*GASR01000102*
3	Thereuopoda longicornis	*GASR01000103*
4	Thereuopoda longicornis	*GASR01000104*
5	Thereuopoda longicornis	*GASR01000105*
6	Thereuopoda longicornis	*GASR01000106*

SLPTX1		
1	GASR01000113	*Thereuopoda longicornis*

Table S3: Sequences Analyzed

2	GASR01000112	*Thereuopoda longicornis*
3	GASR01000111	*Thereuopoda longicornis*
4	GASR01000114	*Thereuopoda longicornis*
5	GASI01000095	*Ethmostigmus rubripes*
6	GASI01000096	*Ethmostigmus rubripes*
7	GASI01000092	*Ethmostigmus rubripes*
8	GASI01000093	*Ethmostigmus rubripes*
9	GASI01000094	*Ethmostigmus rubripes*
10	GASL01000036	*Cormocephalus westwoodi*
11	GASJ01000006	*Ethmostigmus rubripes epidermis*
12	GASJ01000008	*Ethmostigmus rubripes epidermis*
13	GASJ01000007	*Ethmostigmus rubripes epidermis*
14	JZ574149.1	*Scolopendra viridis*

**SLPTX2**		
1	GASI01000163	*Ethmostigmus rubripes*
2	GASI01000164	*Ethmostigmus rubripes*
3	GASI01000165	*Ethmostigmus rubripes*
4	GASI01000166	*Ethmostigmus rubripes*
5	GASI01000168	*Ethmostigmus rubripes*
6	GASI01000167	*Ethmostigmus rubripes*

**SLPTX4**		
1	GASI01000185	*Ethmostigmus rubripes*
2	GASI01000186	*Ethmostigmus rubripes*
3	GASH01000177	*Scolopendra morsitans*
4	GASH01000178	*Scolopendra morsitans*
5	KC144415.1	*Scolopendra subspinipes dehaani*
6	KC144052.1	*Scolopendra subspinipes dehaani*
7	KC144303.1	*Scolopendra subspinipes dehaani*
8	GASI01000187	*Ethmostigmus rubripes*
9	GASI01000188	*Ethmostigmus rubripes*
10	GASI01000189	*Ethmostigmus rubripes*
11	KF130724	*Ethmostigmus rubripes*
12	KF130728	*Ethmostigmus rubripes*
13	KF130727	*Ethmostigmus rubripes*

Table S3: Sequences Analyzed

14	KF130729	*Ethmostigmus rubripes*
15	KF130726	*Ethmostigmus rubripes*
16	KF130730	*Ethmostigmus rubripes*
17	KF130733	*Ethmostigmus rubripes*
18	KF130734	*Ethmostigmus rubripes*
20	KF130731	*Ethmostigmus rubripes*

**SLPTX5**		
1	GASK01000050	*Scolopendra alternans*
2	GASI01000194	*Ethmostigmus rubripes*
3	GASI01000193	*Ethmostigmus rubripes*
4	GASH01000179	*Scolopendra morsitans*
5	GASI01000190	*Ethmostigmus rubripes*
6	GASI01000191	*Ethmostigmus rubripes*
7	GASI01000192	*Ethmostigmus rubripes*
8	GASK01000051	*Scolopendra alternans*
9	GASL01000049	*Cormocephalus westwoodi*

**SLPTX6**		
1	GASH01000180	*Scolopendra morsitans*
2	GASH01000181	*Scolopendra morsitans*
3	GASH01000182	*Scolopendra morsitans*
4	GASH01000183	*Scolopendra morsitans*
5	GASH01000184	*Scolopendra morsitans*
6	GASH01000185	*Scolopendra morsitans*

**SLPTX10**		
1	GASI01000097	*Ethmostigmus rubripes*
2	GASI01000098	*Ethmostigmus rubripes*
3	GASI01000099	*Ethmostigmus rubripes*
4	GASI01000100	*Ethmostigmus rubripes*
5	GASI01000101	*Ethmostigmus rubripes*
6	GASI01000116	*Ethmostigmus rubripes*
7	GASI01000118	*Ethmostigmus rubripes*
8	GASI01000102	*Ethmostigmus rubripes*
9	GASI01000103	*Ethmostigmus rubripes*

Table S3: Sequences Analyzed

10	GASI01000104	*Ethmostigmus rubripes*
11	GASI01000105	*Ethmostigmus rubripes*
12	GASI01000117	*Ethmostigmus rubripes*
13	GASI01000106	*Ethmostigmus rubripes*
14	GASI01000107	*Ethmostigmus rubripes*
15	GASI01000108	*Ethmostigmus rubripes*
16	GASI01000109	*Ethmostigmus rubripes*
17	GASI01000110	*Ethmostigmus rubripes*
18	GASI01000111	*Ethmostigmus rubripes*
19	GASI01000112	*Ethmostigmus rubripes*
20	GASI01000113	*Ethmostigmus rubripes*
21	GASI01000114	*Ethmostigmus rubripes*
22	GASI01000115	*Ethmostigmus rubripes*
23	GASH01000111	*Scolopendra morsitans*
24	GASH01000114	*Scolopendra morsitans*
25	GASH01000115	*Scolopendra morsitans*
26	GASH01000112	*Scolopendra morsitans*
27	GASH01000116	*Scolopendra morsitans*
28	GASH01000113	*Scolopendra morsitans*
29	GASH01000117	*Scolopendra morsitans*
30	GASH01000119	*Scolopendra morsitans*
31	GASH01000118	*Scolopendra morsitans*
32	GASK01000031	*Scolopendra alternans*
33	GASK01000033	*Scolopendra alternans*
34	GASK01000030	*Scolopendra alternans*
35	GASK01000032	*Scolopendra alternans*
36	GASK01000034	*Scolopendra alternans*
37	GASK01000035	*Scolopendra alternans*
38	GASH01000120	*Scolopendra morsitans*
39	GASL01000037	*Cormocephalus westwoodi*
40	GASL01000038	*Cormocephalus westwoodi*
41	GASL01000041	*Cormocephalus westwoodi*
42	GASL01000039	*Cormocephalus westwoodi*
43	GASL01000040	*Cormocephalus westwoodi*
44	JZ574164.1	*Scolopendra viridis*
45	JZ574166.1	*Scolopendra viridis*
46	JZ574142.1	*Scolopendra viridis*

Table S3: Sequences Analyzed

SLPTX11		
1	GASH01000124	*Scolopendra morsitans*
2	GASH01000121	*Scolopendra morsitans*
3	GASH01000123	*Scolopendra morsitans*
4	KC144430.1	*Scolopendra subspinipes dehaani*
5	KC144839.1	*Scolopendra subspinipes dehaani*
6	GASH01000128	*Scolopendra morsitans*
7	GASH01000133	*Scolopendra morsitans*
8	GASK01000037	*Scolopendra alternans*
9	GASH01000129	*Scolopendra morsitans*
10	GASH01000130	*Scolopendra morsitans*
11	GASH01000131	*Scolopendra morsitans*
12	GASH01000132	*Scolopendra morsitans*
13	GASH01000134	*Scolopendra morsitans*
14	GASK01000038	*Scolopendra alternans*
15	GASH01000135	*Scolopendra morsitans*
16	GASH01000125	*Scolopendra morsitans*
17	KC144200.1	*Scolopendra subspinipes dehaani*
18	KC144173.1	*Scolopendra subspinipes dehaani*
19	KC144172.1	*Scolopendra subspinipes dehaani*
20	KC144023.1	*Scolopendra subspinipes dehaani*
21	KC144075.1	*Scolopendra subspinipes dehaani*
22	KC144647.1	*Scolopendra subspinipes dehaani*
23	KC144505.1	*Scolopendra subspinipes dehaani*
24	KC144104.1	*Scolopendra subspinipes dehaani*
25	KC144090.1	*Scolopendra subspinipes dehaani*
26	KC144802.1	*Scolopendra subspinipes dehaani*
27	KC144483.1	*Scolopendra subspinipes dehaani*
28	KC145102.1	*Scolopendra subspinipes dehaani*
29	KC144335.1	*Scolopendra subspinipes dehaani*
30	KC144251.1	*Scolopendra subspinipes dehaani*
31	KC144193.1	*Scolopendra subspinipes dehaani*
32	KC144442.1	*Scolopendra subspinipes dehaani*
33	KC144919.1	*Scolopendra subspinipes dehaani*
34	KC144828.1	*Scolopendra subspinipes dehaani*
35	GASH01000127	*Scolopendra morsitans*

Table S3: Sequences Analyzed

36	GASH01000126	*Scolopendra morsitans*
37	GASH01000122	*Scolopendra morsitans*
38	GASK01000036	*Scolopendra alternans*

**SLPTX12**		
1	GASH01000136	*Scolopendra morsitans*
2	GASH01000137	*Scolopendra morsitans*
3	GASH01000138	*Scolopendra morsitans*
4	GASH01000139	*Scolopendra morsitans*
5	GASH01000140	*Scolopendra morsitans*
6	GASH01000141	*Scolopendra morsitans*
7	GASK01000039	*Scolopendra alternans*
8	GASI01000119	*Ethmostigmus rubripes*
9	GASI01000120	*Ethmostigmus rubripes*
10	GASI01000121	*Ethmostigmus rubripes*

**SLPTX13**		
1	GASI01000122	*Ethmostigmus rubripes*
2	GASI01000123	*Ethmostigmus rubripes*
3	GASI01000124	*Ethmostigmus rubripes*
4	GASH01000142	*Scolopendra morsitans*
5	GASH01000143	*Scolopendra morsitans*
6	GASH01000144	*Scolopendra morsitans*
7	GASK01000040	*Scolopendra alternans*
8	GASL01000042	*Cormocephalus westwoodi*
9	GASL01000043	*Cormocephalus westwoodi*

**SLPTX14**		
1	GASH01000145	*Scolopendra morsitans*
2	GASH01000146	*Scolopendra morsitans*
3	GASH01000147	*Scolopendra morsitans*
4	GASI01000125	*Ethmostigmus rubripes*
5	GASK01000041	*Scolopendra alternans*

**SLPTX15**

Table S3: Sequences Analyzed

1	GASH01000148	*Scolopendra morsitans*
2	GASH01000149	*Scolopendra morsitans*
3	GASH01000150	*Scolopendra morsitans*
4	GASH01000151	*Scolopendra morsitans*
5	KC144976.1	*Scolopendra subspinipes dehaani*
6	KC144272.1	*Scolopendra subspinipes dehaani*
7	KC144973.1	*Scolopendra subspinipes dehaani*
8	GASH01000152	*Scolopendra morsitans*
9	GASH01000153	*Scolopendra morsitans*
10	GASH01000154	*Scolopendra morsitans*
11	GASK01000042	*Scolopendra alternans*
12	KC144115.1	*Scolopendra subspinipes dehaani*
13	KC144793.1	*Scolopendra subspinipes dehaani*
14	KC144364.1	*Scolopendra subspinipes dehaani*
15	KC144588.1	*Scolopendra subspinipes dehaani*
16	KC144400.1	*Scolopendra subspinipes dehaani*
17	KC144464.1	*Scolopendra subspinipes dehaani*
18	GASH01000155	*Scolopendra morsitans*
19	KC144101.1	*Scolopendra subspinipes dehaani*
20	GASI01000126	*Ethmostigmus rubripes*
21	KC145039.1	*Scolopendra subspinipes dehaani*
22	KC144953.1	*Scolopendra subspinipes dehaani*
23	KC144826.1	*Scolopendra subspinipes dehaani*
24	KC145003.1	*Scolopendra subspinipes dehaani*
25	KC145006.1	*Scolopendra subspinipes dehaani*
26	GASK01000043	*Scolopendra alternans*
27	GASK01000044	*Scolopendra alternans*
28	KC144556.1	*Scolopendra subspinipes dehaani*
29	GASH01000156	*Scolopendra morsitans*

**SLPTX16**		
1	KC144377.1	*Scolopendra subspinipes dehaani*
2	GASI01000135	*Ethmostigmus rubripes*
3	GASI01000136	*Ethmostigmus rubripes*
4	GASI01000137	*Ethmostigmus rubripes*
5	GASI01000138	*Ethmostigmus rubripes*
6	GASI01000139	*Ethmostigmus rubripes*

Table S3: Sequences Analyzed

7	GASI01000140	*Ethmostigmus rubripes*
8	GASI01000141	*Ethmostigmus rubripes*
9	GASI01000142	*Ethmostigmus rubripes*
10	GASI01000143	*Ethmostigmus rubripes*
11	GASI01000134	*Ethmostigmus rubripes*
12	GASL01000045	*Cormocephalus westwoodi*
13	GASH01000160	*Scolopendra morsitans*
14	GASH01000161	*Scolopendra morsitans*
15	GASH01000162	*Scolopendra morsitans*
16	GASI01000144	*Ethmostigmus rubripes*
17	GASI01000145	*Ethmostigmus rubripes*
18	GASI01000146	*Ethmostigmus rubripes*
19	GASI01000147	*Ethmostigmus rubripes*
20	GASI01000151	*Ethmostigmus rubripes*
21	GASI01000152	*Ethmostigmus rubripes*
22	GASI01000154	*Ethmostigmus rubripes*
23	GASI01000148	*Ethmostigmus rubripes*
24	GASI01000149	*Ethmostigmus rubripes*
25	GASI01000150	*Ethmostigmus rubripes*
26	GASI01000153	*Ethmostigmus rubripes*
27	GASI01000155	*Ethmostigmus rubripes*
28	GASH01000163	*Scolopendra morsitans*
29	GASH01000164	*Scolopendra morsitans*
30	GASH01000165	*Scolopendra morsitans*
31	GASI01000127	*Ethmostigmus rubripes*
32	GASI01000128	*Ethmostigmus rubripes*
33	GASH01000166	*Scolopendra morsitans*
34	GASH01000167	*Scolopendra morsitans*
35	GASK01000045	*Scolopendra alternans*
36	GASI01000129	*Ethmostigmus rubripes*
37	GASI01000130	*Ethmostigmus rubripes*
38	GASI01000131	*Ethmostigmus rubripes*
39	GASI01000132	*Ethmostigmus rubripes*
40	GASI01000133	*Ethmostigmus rubripes*
41	GASH01000168	*Scolopendra morsitans*
42	SMAR012379	*Strigamia maritima*
43	SMAR009096	*Strigamia maritima*

44	SMAR009097	*Strigamia maritima*

**SLPTX17**		
1	GASI01000156	*Ethmostigmus rubripes*
2	GASI01000157	*Ethmostigmus rubripes*
3	GASI01000158	*Ethmostigmus rubripes*
4	GASI01000159	*Ethmostigmus rubripes*
5	GASI01000160	*Ethmostigmus rubripes*
6	GASJ01000003	*Ethmostigmus rubripes epidermis*
7	GASJ01000004	*Ethmostigmus rubripes epidermis*
8	GASI01000161	*Ethmostigmus rubripes*
9	GASI01000162	*Ethmostigmus rubripes*
10	JZ574151.1	*Scolopendra viridis*

**SLPTX24**		
1	GASI01000177	*Ethmostigmus rubripes*
2	GASI01000178	*Ethmostigmus rubripes*
3	GASI01000179	*Ethmostigmus rubripes*
4	GASI01000180	*Ethmostigmus rubripes*
5	GASI01000181	*Ethmostigmus rubripes*

Spiders

**Phospholipase D**		
1	AY699703	*Loxosceles arizonica*
2	AY699704	*Loxosceles arizonica*
3	KM884812	*Loxosceles arizonica*
4	AY559846	*Loxosceles reclusa*
5	AY559847	*Loxosceles reclusa*
6	AY862486	*Loxosceles reclusa*
7	FJ171486	*Loxosceles spinulosa*
8	FJ171454	*Loxosceles amazonica*
9	FJ171455	*Loxosceles amazonica*
10	FJ171378	*Loxosceles apachea*
11	AY559844	*Loxosceles boneti*
12	AY559845	*Loxosceles boneti*

Table S3: Sequences Analyzed

13	FJ171475	*Loxosceles spinulosa*
14	FJ171407	*Loxosceles deserta*
15	FJ171408	*Loxosceles deserta*
16	FJ171409	*Loxosceles deserta*
17	FJ171410	*Loxosceles deserta*
18	FJ171411	*Loxosceles deserta*
19	FJ171412	*Loxosceles deserta*
20	FJ171413	*Loxosceles deserta*
21	FJ171414	*Loxosceles deserta*
22	FJ171415	*Loxosceles deserta*
23	AY974250	*Loxosceles gaucho*
24	JX866729	*Loxosceles gaucho*
25	FJ171369	*Loxosceles hirsuta*
26	FJ171370	*Loxosceles hirsuta*
27	FJ171371	*Loxosceles hirsuta*
28	FJ171372	*Loxosceles hirsuta*
29	FJ171373	*Loxosceles hirsuta*
30	AY340702	*Loxosceles intermedia*
31	AY304471.2	*Loxosceles intermedia*
32	AY304472.2	*Loxosceles intermedia*
33	DQ218155	*Loxosceles intermedia*
34	DQ266399	*Loxosceles intermedia*
35	DQ267927	*Loxosceles intermedia*
36	DQ388596	*Loxosceles intermedia*
37	DQ388597	*Loxosceles intermedia*
38	DQ431848	*Loxosceles intermedia*
39	DQ431849	*Loxosceles intermedia*
40	EF474482	*Loxosceles intermedia*
41	EF535250	*Loxosceles intermedia*
42	EF535251	*Loxosceles intermedia*
43	EF535252	*Loxosceles intermedia*
44	EF535253	*Loxosceles intermedia*
45	EF535255	*Loxosceles intermedia*
46	EF535256	*Loxosceles intermedia*
47	KC237286	*Loxosceles intermedia*
48	AY093601	*Loxosceles laeta*
49	FJ171435	*Loxosceles rufescens*

Table S3: Sequences Analyzed

50	AY929305	*Loxosceles similis*
51	FJ171361	*Loxosceles spadicea*
52	FJ171362	*Loxosceles spadicea*
53	FJ171363	*Loxosceles spadicea*
54	FJ171365	*Loxosceles spadicea*
55	FJ171366	*Loxosceles spadicea*
56	FJ171367	*Loxosceles spadicea*
57	FJ171374	*Loxosceles spadicea*
58	FJ171375	*Loxosceles spadicea*
59	FJ171484	*Loxosceles spinulosa*
60	FJ171485	*Loxosceles spinulosa*
61	FJ171527	*Loxosceles spinulosa*
62	FJ171529	*Loxosceles spinulosa*
63	FJ171526	*Sicarius albospinosus*
64	FJ171490	*Sicarius damarensis*
65	FJ171506	*Sicarius damarensis*
66	FJ171507	*Sicarius damarensis*
67	FJ171511	*Sicarius damarensis*
68	FJ171512	*Sicarius damarensis*
69	FJ171514	*Sicarius damarensis*
70	FJ171515	*Sicarius damarensis*
71	FJ171516	*Sicarius damarensis*
72	FJ171517	*Sicarius damarensis*
73	FJ171522	*Sicarius damarensis*
74	FJ171524	*Sicarius damarensis*
75	FJ171525	*Sicarius damarensis*
76	FJ171487	*Sicarius patagonicus*
77	FJ171503	*Sicarius patagonicus*
78	FJ171504	*Sicarius patagonicus*
79	FJ171505	*Sicarius patagonicus*
80	FJ171468	*Sicarius peruensis*
81	FJ171469	*Sicarius peruensis*
82	FJ171470	*Sicarius peruensis*
83	FJ171471	*Sicarius peruensis*
84	FJ171472	*Sicarius peruensis*
85	FJ171473	*Sicarius peruensis*
86	FJ171488	*Sicarius peruensis*

Table S3: Sequences Analyzed

87	FJ171491	*Sicarius peruensis*
88	FJ171492	*Sicarius peruensis*
89	FJ171493	*Sicarius peruensis*
90	FJ171494	*Sicarius peruensis*
91	FJ171495	*Sicarius peruensis*
92	FJ171496	*Sicarius peruensis*
93	FJ171497	*Sicarius peruensis*
94	FJ171498	*Sicarius peruensis*
95	FJ171499	*Sicarius peruensis*
96	FJ171500	*Sicarius peruensis*
97	FJ171501	*Sicarius peruensis*
98	FJ171474	*Sicarius terrosus*
99	FJ171489	*Sicarius terrosus*
100	KM884813	*Sicarius terrosus*

**Huwentoxin-family 1**		
1	AF157504.1	*Ornithoctonus huwena*
2	AY263711.1	*Ornithoctonus huwena*
3	EU195252.1	*Ornithoctonus huwena*
4	EU195253.1	*Ornithoctonus huwena*
5	EU195254.1	*Ornithoctonus huwena*
6	EU195255.1	*Ornithoctonus huwena*
7	EU195256.1	*Ornithoctonus huwena*
8	EU195257.1	*Ornithoctonus huwena*
9	EU195258.1	*Ornithoctonus huwena*
10	EU195259.1	*Ornithoctonus huwena*
11	EU195260.1	*Ornithoctonus huwena*
12	EU195261.1	*Ornithoctonus huwena*
13	EU195262.1	*Ornithoctonus huwena*
14	EU195263.1	*Ornithoctonus huwena*
15	GU293008.1	*Ornithoctonus hainana*
16	GU293009.1	*Ornithoctonus hainana*
17	GU293010.1	*Ornithoctonus hainana*
18	GU293060.1	*Ornithoctonus hainana*
19	GU293061.1	*Ornithoctonus hainana*
20	GU293062.1	*Ornithoctonus hainana*
21	GU293063.1	*Ornithoctonus hainana*

Table S3: Sequences Analyzed

22	GU292856.1	*Ornithoctonus hainana*
23	GU292857.1	*Ornithoctonus hainana*
24	GU292858.1	*Ornithoctonus hainana*
25	GU292859.1	*Ornithoctonus hainana*
26	GU292860.1	*Ornithoctonus hainana*
27	GU292861.1	*Ornithoctonus hainana*
28	GU292862.1	*Ornithoctonus hainana*
29	GU292863.1	*Ornithoctonus hainana*
30	GU292864.1	*Ornithoctonus hainana*
31	GU292865.1	*Ornithoctonus hainana*
32	GU292866.1	*Ornithoctonus hainana*
33	GU292867.1	*Ornithoctonus hainana*
34	GU292868.1	*Ornithoctonus hainana*
35	GU292869.1	*Ornithoctonus hainana*
36	GU292870.1	*Ornithoctonus hainana*
37	GU292871.1	*Ornithoctonus hainana*
38	GU292872.1	*Ornithoctonus hainana*
39	GU292873.1	*Ornithoctonus hainana*
40	GU292874.1	*Ornithoctonus hainana*
41	GU292875.1	*Ornithoctonus hainana*
42	GU292876.1	*Ornithoctonus hainana*
43	GU292877.1	*Ornithoctonus hainana*
44	GU292878.1	*Ornithoctonus hainana*
45	GU292879.1	*Ornithoctonus hainana*
46	GU292880.1	*Ornithoctonus hainana*
47	AJ854060.1	*Chilobrachys jingzhao*
48	EU233831.1	*Chilobrachys jingzhao*
49	EU233832.1	*Chilobrachys jingzhao*
50	EU233833.1	*Chilobrachys jingzhao*
51	EU233834.1	*Chilobrachys jingzhao*
52	EU233835.1	*Chilobrachys jingzhao*
53	EU233848.1	*Chilobrachys jingzhao*
54	EU233849.1	*Chilobrachys jingzhao*
55	EU233850.1	*Chilobrachys jingzhao*
56	EU233851.1	*Chilobrachys jingzhao*
57	EU233852.1	*Chilobrachys jingzhao*
58	EU233853.1	*Chilobrachys jingzhao*

Table S3: Sequences Analyzed

59	EU233855.1	*Chilobrachys jingzhao*
60	EU233856.1	*Chilobrachys jingzhao*
61	EU233857.1	*Chilobrachys jingzhao*
62	EU233858.1	*Chilobrachys jingzhao*
63	EU233859.1	*Chilobrachys jingzhao*
64	EU233860.1	*Chilobrachys jingzhao*
65	EU233861.1	*Chilobrachys jingzhao*
66	EU233863.1	*Chilobrachys jingzhao*
67	EU233864.1	*Chilobrachys jingzhao*
68	EU233865.1	*Chilobrachys jingzhao*
69	EU233866.1	*Chilobrachys jingzhao*
70	EU233867.1	*Chilobrachys jingzhao*
71	EU233868.1	*Chilobrachys jingzhao*
72	EU233869.1	*Chilobrachys jingzhao*
73	EU233870.1	*Chilobrachys jingzhao*
74	EU233871.1	*Chilobrachys jingzhao*
75	EU233872.1	*Chilobrachys jingzhao*
76	EU233874.1	*Chilobrachys jingzhao*
77	EU233875.1	*Chilobrachys jingzhao*
78	EU233876.1	*Chilobrachys jingzhao*
79	EU233877.1	*Chilobrachys jingzhao*
80	EU233878.1	*Chilobrachys jingzhao*
81	EU233879.1	*Chilobrachys jingzhao*
82	EU233880.1	*Chilobrachys jingzhao*
83	EU233881.1	*Chilobrachys jingzhao*
84	EU233882.1	*Chilobrachys jingzhao*
85	EU233883.1	*Chilobrachys jingzhao*
86	EU233884.1	*Chilobrachys jingzhao*
87	EU233885.1	*Chilobrachys jingzhao*
88	EU233886.1	*Chilobrachys jingzhao*
89	EU233887.1	*Chilobrachys jingzhao*
90	EU233888.1	*Chilobrachys jingzhao*
91	EU233889.1	*Chilobrachys jingzhao*
92	EU233890.1	*Chilobrachys jingzhao*
93	EU233891.1	*Chilobrachys jingzhao*
94	EU233892.1	*Chilobrachys jingzhao*
95	EU233893.1	*Chilobrachys jingzhao*

Table S3: Sequences Analyzed

96	EU233894.1	*Chilobrachys jingzhao*
97	EU233895.1	*Chilobrachys jingzhao*
98	EU233896.1	*Chilobrachys jingzhao*
99	EU233897.1	*Chilobrachys jingzhao*
100	EU233898.1	*Chilobrachys jingzhao*
101	EU233899.1	*Chilobrachys jingzhao*
102	EU233900.1	*Chilobrachys jingzhao*
103	EU233901.1	*Chilobrachys jingzhao*
104	EU233902.1	*Chilobrachys jingzhao*
105	EU233903.1	*Chilobrachys jingzhao*
106	EU233910.1	*Chilobrachys jingzhao*
107	AY263707.1	*Ornithoctonus huwena*
108	AY263708.1	*Ornithoctonus huwena*
109	EU195264.1	*Ornithoctonus huwena*
110	EU195265.1	*Ornithoctonus huwena*
111	EU195266.1	*Ornithoctonus huwena*
112	EU195267.1	*Ornithoctonus huwena*
113	EU195268.1	*Ornithoctonus huwena*
114	EU195269.1	*Ornithoctonus huwena*
115	EU195270.1	*Ornithoctonus huwena*
116	EU195271.1	*Ornithoctonus huwena*
117	EU195291.1	*Ornithoctonus huwena*
118	EU195292.1	*Ornithoctonus huwena*
119	EU195293.1	*Ornithoctonus huwena*
120	HM015001.1	*Ornithoctonus huwena*
121	AY316118.1	*Grammostola rosea*
122	AB612243.1	*Grammostola rosea*
123	GU293014.1	*Ornithoctonus hainana*
124	GU293025.1	*Ornithoctonus hainana*
125	GU293026.1	*Ornithoctonus hainana*
126	GU293027.1	*Ornithoctonus hainana*
127	GU293028.1	*Ornithoctonus hainana*
128	GU293040.1	*Ornithoctonus hainana*
129	GU293041.1	*Ornithoctonus hainana*
130	GU293042.1	*Ornithoctonus hainana*
131	GU293043.1	*Ornithoctonus hainana*
132	GU293044.1	*Ornithoctonus hainana*

Table S3: Sequences Analyzed

133	GU293045.1	*Ornithoctonus hainana*
134	GU293046.1	*Ornithoctonus hainana*
135	GU293050.1	*Ornithoctonus hainana*
136	GU293051.1	*Ornithoctonus hainana*
137	GU293052.1	*Ornithoctonus hainana*
138	GU293053.1	*Ornithoctonus hainana*
139	GU293054.1	*Ornithoctonus hainana*
140	GU293055.1	*Ornithoctonus hainana*
141	GU293091.1	*Ornithoctonus hainana*
142	GU293092.1	*Ornithoctonus hainana*
143	GU293093.1	*Ornithoctonus hainana*
144	GU293094.1	*Ornithoctonus hainana*
145	GU293095.1	*Ornithoctonus hainana*
146	GU293096.1	*Ornithoctonus hainana*
147	GU293097.1	*Ornithoctonus hainana*
148	GU293098.1	*Ornithoctonus hainana*
149	GU293099.1	*Ornithoctonus hainana*
150	GU293100.1	*Ornithoctonus hainana*
151	GU293101.1	*Ornithoctonus hainana*
152	GU293102.1	*Ornithoctonus hainana*
153	GU293103.1	*Ornithoctonus hainana*
154	GU293104.1	*Ornithoctonus hainana*
155	GU293105.1	*Ornithoctonus hainana*
156	GU293106.1	*Ornithoctonus hainana*
157	GU293107.1	*Ornithoctonus hainana*
158	GU293108.1	*Ornithoctonus hainana*
159	GU293109.1	*Ornithoctonus hainana*
160	GU293110.1	*Ornithoctonus hainana*
161	GU293111.1	*Ornithoctonus hainana*
162	GU293112.1	*Ornithoctonus hainana*
163	GU293113.1	*Ornithoctonus hainana*
164	GU293114.1	*Ornithoctonus hainana*
165	GU292853.1	*Ornithoctonus hainana*
166	GU292854.1	*Ornithoctonus hainana*
167	GU292855.1	*Ornithoctonus hainana*
168	GU292909.1	*Ornithoctonus hainana*
169	GU292910.1	*Ornithoctonus hainana*

Table S3: Sequences Analyzed

170	GU292911.1	*Ornithoctonus hainana*
171	GU292912.1	*Ornithoctonus hainana*
172	GU292913.1	*Ornithoctonus hainana*
173	GU292914.1	*Ornithoctonus hainana*
174	GU292915.1	*Ornithoctonus hainana*
175	GU292916.1	*Ornithoctonus hainana*
176	GU292917.1	*Ornithoctonus hainana*
177	GU292918.1	*Ornithoctonus hainana*
178	GU292919.1	*Ornithoctonus hainana*
179	GU292920.1	*Ornithoctonus hainana*
180	GU292921.1	*Ornithoctonus hainana*
181	GU292922.1	*Ornithoctonus hainana*
182	GU292923.1	*Ornithoctonus hainana*
183	GU292924.1	*Ornithoctonus hainana*
184	GU292925.1	*Ornithoctonus hainana*
185	GU292926.1	*Ornithoctonus hainana*
186	GU292927.1	*Ornithoctonus hainana*
187	GU292977.1	*Ornithoctonus hainana*
188	GU292978.1	*Ornithoctonus hainana*
189	GU292979.1	*Ornithoctonus hainana*
190	GU292980.1	*Ornithoctonus hainana*
191	GU292981.1	*Ornithoctonus hainana*
192	GU292982.1	*Ornithoctonus hainana*
193	GU292983.1	*Ornithoctonus hainana*
194	GU292984.1	*Ornithoctonus hainana*
195	GU292985.1	*Ornithoctonus hainana*
196	GU292986.1	*Ornithoctonus hainana*
197	GU292987.1	*Ornithoctonus hainana*
198	GU292988.1	*Ornithoctonus hainana*
199	GU292989.1	*Ornithoctonus hainana*
200	GU292990.1	*Ornithoctonus hainana*
201	GU292991.1	*Ornithoctonus hainana*
202	GU292992.1	*Ornithoctonus hainana*
203	GU292998.1	*Ornithoctonus hainana*
204	GU293000.1	*Ornithoctonus hainana*

**Kunitz Protease inhibitor**

Table S3: Sequences Analyzed

1	EU195290	*Ornithoctonus huwena*
2	EU635744	*Ornithoctonus huwena*
3	EU635745	*Ornithoctonus huwena*
4	EU635746	*Ornithoctonus huwena*
5	EU635747	*Ornithoctonus huwena*
6	EU635748	*Ornithoctonus huwena*
7	KF160294	*Haplopelma schmidti*
8	KF160295	*Haplopelma schmidti*
9	KF160296	*Haplopelma schmidti*
10	KF160297	*Haplopelma schmidti*
11	KF160298	*Haplopelma schmidti*
12	KF160299	*Haplopelma schmidti*
13	KF160300	*Haplopelma schmidti*
15	KF160302	*Haplopelma schmidti*
16	KF160303	*Haplopelma schmidti*
17	KF160304	*Haplopelma schmidti*
18	KF160305	*Haplopelma schmidti*
19	KF160306	*Haplopelma schmidti*
20	KF160307	*Haplopelma schmidti*
21	KF160308	*Haplopelma schmidti*
22	KF160309	*Haplopelma schmidti*
23	KF160310	*Haplopelma schmidti*
24	KF160311	*Haplopelma schmidti*
25	KF160312	*Haplopelma schmidti*
26	KF160313	*Haplopelma schmidti*
27	KF160314	*Haplopelma schmidti*
29	KF160316	*Haplopelma schmidti*
30	KF160317	*Haplopelma schmidti*
31	KF160318	*Haplopelma schmidti*
32	KF160319	*Haplopelma schmidti*
33	KF160320	*Haplopelma schmidti*
34	KF160321	*Haplopelma schmidti*
36	KF160323	*Haplopelma schmidti*
37	KF160324	*Haplopelma schmidti*
38	KF160325	*Haplopelma schmidti*
39	KF160326	*Haplopelma schmidti*
40	KF160327	*Haplopelma schmidti*

Table S3: Sequences Analyzed

41	KF160328	*Haplopelma schmidti*
42	KF160329	*Haplopelma schmidti*
43	KF160330	*Haplopelma schmidti*
44	GU293030	*Ornithoctonus hainana*
45	GU293031	*Ornithoctonus hainana*
46	GU293032	*Ornithoctonus hainana*
47	GU293033	*Ornithoctonus hainana*
48	GU293034	*Ornithoctonus hainana*
49	GU293035	*Ornithoctonus hainana*
50	GU293036	*Ornithoctonus hainana*
51	GU293037	*Ornithoctonus hainana*
52	GU293038	*Ornithoctonus hainana*
53	GU293039	*Ornithoctonus hainana*
54	GU293128	*Ornithoctonus hainana*
55	GU293129	*Ornithoctonus hainana*
56	GU293130	*Ornithoctonus hainana*
57	GU293131	*Ornithoctonus hainana*
58	GU293132	*Ornithoctonus hainana*
59	GU293133	*Ornithoctonus hainana*
60	GU293134	*Ornithoctonus hainana*
61	GU293135	*Ornithoctonus hainana*
62	GU293136	*Ornithoctonus hainana*
63	GU292999	*Ornithoctonus hainana*

**Magi-1 family**		
1	EU196048.1	*Brachypelma smithi*
2	EU233907.1	*Chilobrachys jingzhao*
3	EU233908.1	*Chilobrachys jingzhao*
4	EU195239.1	*Ornithoctonus huwena*
5	EU195240.1	*Ornithoctonus huwena*
6	EU195241.1	*Ornithoctonus huwena*
7	EU195242.1	*Ornithoctonus huwena*
8	EU195243.1	*Ornithoctonus huwena*
9	EU195244.1	*Ornithoctonus huwena*
10	EU195245.1	*Ornithoctonus huwena*
11	EU195246.1	*Ornithoctonus huwena*
12	EU195247.1	*Ornithoctonus huwena*

Table S3: Sequences Analyzed

13	EU195248.1	*Ornithoctonus huwena*
14	EU195249.1	*Ornithoctonus huwena*
15	EU195250.1	*Ornithoctonus huwena*
16	EU195251.1	*Ornithoctonus huwena*
17	GU293056.1	*Ornithoctonus hainana*
18	GU293057.1	*Ornithoctonus hainana*
19	GU293058.1	*Ornithoctonus hainana*
20	GU293059.1	*Ornithoctonus hainana*
21	GU293120.1	*Ornithoctonus hainana*
22	GU293121.1	*Ornithoctonus hainana*
23	GU293122.1	*Ornithoctonus hainana*
24	GU293123.1	*Ornithoctonus hainana*
25	GU293124.1	*Ornithoctonus hainana*
26	GU293125.1	*Ornithoctonus hainana*
27	GU293126.1	*Ornithoctonus hainana*
28	GU292930.1	*Ornithoctonus hainana*
29	GU292931.1	*Ornithoctonus hainana*
30	GU292932.1	*Ornithoctonus hainana*
31	GU292933.1	*Ornithoctonus hainana*
32	GU292934.1	*Ornithoctonus hainana*
33	GU292935.1	*Ornithoctonus hainana*
34	GU292936.1	*Ornithoctonus hainana*
35	GU292937.1	*Ornithoctonus hainana*
36	GU292938.1	*Ornithoctonus hainana*
37	GU292939.1	*Ornithoctonus hainana*
38	GU292940.1	*Ornithoctonus hainana*
39	GU292941.1	*Ornithoctonus hainana*
40	GU292942.1	*Ornithoctonus hainana*
41	GU292943.1	*Ornithoctonus hainana*
42	GU292944.1	*Ornithoctonus hainana*
43	GU292945.1	*Ornithoctonus hainana*
44	GU292946.1	*Ornithoctonus hainana*
45	GU292947.1	*Ornithoctonus hainana*
46	GU292948.1	*Ornithoctonus hainana*
47	GU292949.1	*Ornithoctonus hainana*
48	GU292950.1	*Ornithoctonus hainana*
49	GU292951.1	*Ornithoctonus hainana*

Table S3: Sequences Analyzed

50	GU292952.1	*Ornithoctonus hainana*
51	GU292953.1	*Ornithoctonus hainana*
52	GU292954.1	*Ornithoctonus hainana*
53	GU292955.1	*Ornithoctonus hainana*
54	GU292956.1	*Ornithoctonus hainana*
55	GU292957.1	*Ornithoctonus hainana*
56	GU292958.1	*Ornithoctonus hainana*
57	GU292959.1	*Ornithoctonus hainana*
58	GU292960.1	*Ornithoctonus hainana*
59	GU292961.1	*Ornithoctonus hainana*
60	GU292962.1	*Ornithoctonus hainana*
61	GU292963.1	*Ornithoctonus hainana*
62	GU292964.1	*Ornithoctonus hainana*
63	GU292965.1	*Ornithoctonus hainana*
64	GU292966.1	*Ornithoctonus hainana*
65	GU292967.1	*Ornithoctonus hainana*
66	GU292968.1	*Ornithoctonus hainana*
67	GU292969.1	*Ornithoctonus hainana*
68	GU292970.1	*Ornithoctonus hainana*
69	GU292971.1	*Ornithoctonus hainana*
70	GU292972.1	*Ornithoctonus hainana*
71	GU292973.1	*Ornithoctonus hainana*
72	GU292974.1	*Ornithoctonus hainana*
73	GU292975.1	*Ornithoctonus hainana*
74	GU292976.1	*Ornithoctonus hainana*
75	GU170865.1	*Citharischius crawshayi*
76	EF219061.1	*Lasiodora sp*
77	AB121200.1	*Macrothele gigas*
78	AB121202.1	*Macrothele gigas*

**Alpha-latrotoxin**		
1	Z14086.1	*Latrodectus tredecimguttatus*
2	X55009.1	*Latrodectus tredecimguttatus*
3	X92679.1	*Latrodectus tredecimguttatus*
4	AF069521.1	*Latrodectus tredecimguttatus*
5	AF134162.1	*Latrodectus tredecimguttatus*
6	KC414059.1	*Latrodectus mactans*

Table S3: Sequences Analyzed

7	KC414066.1	*Latrodectus tredecimguttatus*
8	KC414067.1	*Latrodectus tredecimguttatus*
9	KC414034.1	*Latrodectus tredecimguttatus*
10	KC414048.1	*Latrodectus geometricus*
11	KC414049.1	*Latrodectus geometricus*
12	KC414050.1	*Latrodectus geometricus*
13	KC414051.1	*Latrodectus geometricus*
14	KC414052.1	*Latrodectus geometricus*
15	KC414036.1	*Latrodectus geometricus*
19	KC414042.1	*Latrodectus antheratus*
20	KC414044.1	*Latrodectus corallinus*
21	KC414045.1	*Latrodectus corallinus*
22	KC414046.1	*Latrodectus diaguita*
23	KC414047.1	*Latrodectus diaguita*
24	KC414060.1	*Latrodectus mirabilis*
25	KC414061.1	*Latrodectus mirabilis*
26	KC414062.1	*Latrodectus pallidus*
27	KC414038.1	*Latrodectus pallidus*
28	KC414063.1	*Latrodectus renivulvatus*
29	KC414064.1	*Latrodectus rhodesiensis*
30	KC414065.1	*Latrodectus rhodesiensis*
31	KC414041.1	*Latrodectus sp.*
32	KC414040.1	*Latrodectus sp.*
33	KC414035.1	*Latrodectus sp.*
34	KC414068.1	*Latrodectus variegatus*
35	KC414070.1	*Latrodectus variolus*
36	KC414043.1	*Latrodectus bishopi*
37	FR851877.1	*Latrodectus hasseltii*
38	KC414054.1	*Latrodectus hasseltii*
39	KC414055.1	*Latrodectus hasseltii*
40	KC414033.1	*Latrodectus hasseltii*
41	KC414071.1	*Steatoda capensis*
42	KC414072.1	*Steatoda grossa*
43	KC414039.1	*Steatoda grossa*
44	KC414057.1	*Latrodectus katipo*
45	KC414058.1	*Latrodectus katipo*
46	KC414056.1	*Latrodectus hesperus*

Table S3: Sequences Analyzed

47	KC414032.1	*Latrodectus hesperus*
48	KC414037.1	Latrodectus hesperus
49	KM382064.1	Latrodectus hesperus

**U1-lycotoxin family**		
1	EU925924.1	*Lycosa singoriensis*
2	EU925925.1	*Lycosa singoriensis*
3	EU925926.1	*Lycosa singoriensis*
4	EU925927.1	*Lycosa singoriensis*
5	EU925928.1	*Lycosa singoriensis*
6	EU925929.1	*Lycosa singoriensis*
7	EU925930.1	*Lycosa singoriensis*
8	EU925931.1	*Lycosa singoriensis*
9	EU925932.1	*Lycosa singoriensis*
10	EU925933.1	*Lycosa singoriensis*
11	EU925934.1	*Lycosa singoriensis*
12	EU925935.1	*Lycosa singoriensis*
13	EU925936.1	*Lycosa singoriensis*
14	EU925937.1	*Lycosa singoriensis*
15	EU925938.1	*Lycosa singoriensis*
16	EU925939.1	*Lycosa singoriensis*
17	EU925940.1	*Lycosa singoriensis*
18	EU925941.1	*Lycosa singoriensis*
19	EU925942.1	*Lycosa singoriensis*
20	EU925943.1	*Lycosa singoriensis*
21	EU925944.1	*Lycosa singoriensis*
22	EU925945.1	*Lycosa singoriensis*
23	EU925946.1	*Lycosa singoriensis*
24	EU925947.1	*Lycosa singoriensis*
25	EU925948.1	*Lycosa singoriensis*
26	EU925949.1	*Lycosa singoriensis*
27	EU925950.1	*Lycosa singoriensis*
28	EU925951.1	*Lycosa singoriensis*
29	EU925952.1	*Lycosa singoriensis*
30	EU925953.1	*Lycosa singoriensis*
31	EU925954.1	*Lycosa singoriensis*
32	EU925955.1	*Lycosa singoriensis*

Table S3: Sequences Analyzed

33	EU925956.1	*Lycosa singoriensis*
34	EU925957.1	*Lycosa singoriensis*
35	EU925958.1	*Lycosa singoriensis*
36	EU925959.1	*Lycosa singoriensis*
37	EU925960.1	*Lycosa singoriensis*
38	EU925961.1	*Lycosa singoriensis*
39	EU925962.1	*Lycosa singoriensis*
40	EU925963.1	*Lycosa singoriensis*
41	EU925964.1	*Lycosa singoriensis*
42	EU925965.1	*Lycosa singoriensis*
43	EU925966.1	*Lycosa singoriensis*
44	EU925967.1	*Lycosa singoriensis*
45	EU925968.1	*Lycosa singoriensis*
46	EU925969.1	*Lycosa singoriensis*
47	EU925970.1	*Lycosa singoriensis*
48	EU925971.1	*Lycosa singoriensis*
49	EU925972.1	*Lycosa singoriensis*
50	EU925973.1	*Lycosa singoriensis*
51	EU925974.1	*Lycosa singoriensis*
52	EU925975.1	*Lycosa singoriensis*
53	EU925976.1	*Lycosa singoriensis*
54	EU925977.1	*Lycosa singoriensis*
55	EU925978.1	*Lycosa singoriensis*
56	EU925979.1	*Lycosa singoriensis*
57	FM863928.1	*Lycosa singoriensis*
58	FM863929.1	*Lycosa singoriensis*
59	FM863931.1	*Lycosa singoriensis*
60	FM863932.1	*Lycosa singoriensis*
61	FM863934.1	*Lycosa singoriensis*
62	FM863935.1	*Lycosa singoriensis*
63	FM863937.1	*Lycosa singoriensis*
64	FM863938.1	*Lycosa singoriensis*
65	FM863939.1	*Lycosa singoriensis*
66	FM863940.1	*Lycosa singoriensis*
67	FM863941.1	*Lycosa singoriensis*
68	FM863942.1	*Lycosa singoriensis*
69	FM863943.1	*Lycosa singoriensis*

Table S3: Sequences Analyzed

70	FM863944.1	*Lycosa singoriensis*
71	FM863945.1	*Lycosa singoriensis*
72	FM863946.1	*Lycosa singoriensis*
73	FM863947.1	*Lycosa singoriensis*
74	FM863948.1	*Lycosa singoriensis*
75	FM863949.1	*Lycosa singoriensis*
76	FM863950.1	*Lycosa singoriensis*
77	FM863951.1	*Lycosa singoriensis*
78	FM863952.1	*Lycosa singoriensis*
79	FM863953.1	*Lycosa singoriensis*
80	FM863954.1	*Lycosa singoriensis*
81	FM863955.1	*Lycosa singoriensis*
82	FM863956.1	*Lycosa singoriensis*
83	FM863957.1	*Lycosa singoriensis*
84	FM863958.1	*Lycosa singoriensis*
85	FM863959.1	*Lycosa singoriensis*
86	FM863960.1	*Lycosa singoriensis*
87	FM863961.1	*Lycosa singoriensis*
88	FM863962.1	*Lycosa singoriensis*
89	FM863963.1	*Lycosa singoriensis*
90	FM863964.1	*Lycosa singoriensis*
91	FM863965.1	*Lycosa singoriensis*
92	FM863966.1	*Lycosa singoriensis*
93	FM863967.1	*Lycosa singoriensis*
94	FM863968.1	*Lycosa singoriensis*
95	FM863969.1	*Lycosa singoriensis*
96	FM863970.1	*Lycosa singoriensis*
97	FM863971.1	*Lycosa singoriensis*
98	FM863972.1	*Lycosa singoriensis*
99	FM863973.1	*Lycosa singoriensis*
100	FM863974.1	*Lycosa singoriensis*
101	FM863975.1	*Lycosa singoriensis*
102	FM863976.1	*Lycosa singoriensis*
103	FM863977.1	*Lycosa singoriensis*
104	FM863978.1	*Lycosa singoriensis*
105	FM863979.1	*Lycosa singoriensis*
106	FM863980.1	*Lycosa singoriensis*

Table S3: Sequences Analyzed

107	FM863981.1	*Lycosa singoriensis*
108	FM863982.1	*Lycosa singoriensis*
109	FM863983.1	*Lycosa singoriensis*

**ω-toxins**		
1	EF523494.1	*Atrax robustus*
2	EF523495.1	*Atrax robustus*
3	EF523496.1	*Atrax robustus*
4	EF523497.1	*Atrax robustus*
5	EF523498.1	*Atrax robustus*
6	EF523499.1	*Atrax robustus*
7	HG001283.1	*Hadronyche infensa*
8	HG001284.1	*Hadronyche infensa*
9	HG001286.1	*Hadronyche infensa*
10	HG001304.1	*Hadronyche infensa*
11	HG001305.1	*Hadronyche infensa*
12	HG001306.1	*Hadronyche infensa*
13	HG001307.1	*Hadronyche infensa*
14	HG001285.1	*Hadronyche venenata*
15	HG001287.1	*Hadronyche venenata*
16	HG001288.1	*Hadronyche venenata*
17	HG001289.1	*Hadronyche venenata*
18	HG001290.1	*Hadronyche venenata*
19	HG001291.1	*Hadronyche venenata*
20	HG001292.1	*Hadronyche venenata*
21	HG001313.1	*Hadronyche modesta*
22	HG001314.1	*Hadronyche modesta*
23	HG001315.1	*Hadronyche modesta*
24	HG001316.1	*Hadronyche modesta*
25	HG001317.1	*Hadronyche modesta*
26	HG001318.1	*Hadronyche modesta*
27	HG001319.1	*Hadronyche modesta*

**κ-toxins**		
1	HG001310.1	*Hadronyche versuta*
2	HG001311.1	*Hadronyche versuta*

Table S3: Sequences Analyzed

3	Hv1c_2	*Hadronyche versuta*
4	Hv1c_3	*Hadronyche versuta*
5	Hv1c_4	*Hadronyche versuta*
6	Hv1e	*Hadronyche versuta*
7	Hv1d	*Hadronyche versuta*
8	Hvn1a_2	*Hadronyche venenata*
9	Hmo1a	*Hadronyche modesta*

**ICKs**		
1	GAQE00000004	*Trittame loki*
2	GAQE00000005	*Trittame loki*
3	GAQE00000006	*Trittame loki*
4	GAQE00000007	*Trittame loki*
5	GAQE00000008	*Trittame loki*
6	GAQE00000009	*Trittame loki*
7	GAQE00000010	*Trittame loki*
8	GAQE00000011	*Trittame loki*
9	GAQE00000012	*Trittame loki*
10	GAQE00000013	*Trittame loki*
11	GAQE00000014	*Trittame loki*
12	GAQE00000015	*Trittame loki*
13	GAQE00000016	*Trittame loki*
14	GAQE00000017	*Trittame loki*
15	GAQE00000018	*Trittame loki*
16	GAQE00000019	*Trittame loki*
17	GAQE00000020	*Trittame loki*
18	GAQE00000021	*Trittame loki*
19	GAQE00000022	*Trittame loki*
20	GAQE00000023	*Trittame loki*
21	GAQE00000024	*Trittame loki*
22	GAQE00000025	*Trittame loki*
23	GAQE00000026	*Trittame loki*
24	GAQE00000027	*Trittame loki*
25	GAQE00000028	*Trittame loki*
26	GAQE00000029	*Trittame loki*
27	GAQE00000030	*Trittame loki*
28	GAQE00000031	*Trittame loki*

Table S3: Sequences Analyzed

29	GAQE00000032	*Trittame loki*
30	GAQE00000033	*Trittame loki*
31	GAQE00000034	*Trittame loki*
32	GAQE00000035	*Trittame loki*
33	GAQE00000036	*Trittame loki*
34	GAQE00000037	*Trittame loki*
35	GAQE00000038	*Trittame loki*
36	GAQE00000039	*Trittame loki*
37	GAQE00000040	*Trittame loki*
38	GAQE00000041	*Trittame loki*
39	GAQE00000042	*Trittame loki*
40	GAQE00000043	*Trittame loki*
41	GAQE00000044	*Trittame loki*
42	GAQE00000045	*Trittame loki*
43	EU233886.1	*Chilobrachys jingzhao*
44	EU233887.1	*Chilobrachys jingzhao*
45	EU233884.1	*Chilobrachys jingzhao*
46	EU233902.1	*Chilobrachys jingzhao*
47	AB200997.1	*Grammostola rosea*

Cnidaria

**Actinoporins**		
1	AB175824.1	*Actineria villosa*
2	AB512460.1	*Hydra magnipapillata*
3	AB512462.1	*Hydra magnipapillata*
4	AB512463.1	*Hydra magnipapillata*
5	U41661.1	*Actinia equina*
6	U51900.1	*Actinia equina*
7	AF057028.1	*Actinia equina*
8	FM958450.1	*Actinia fragacea*
9	AB479475.1	*Anthopleura asiatica*
10	AY855350.1	*Heteractis crispa*
11	AF170706.1	*Heteractis magnifica*
12	XM_004206398.1	*Hydra magnipapillata*
13	XM_002166243.2	*Hydra magnipapillata*
14	XM_002153869.2	*Hydra magnipapillata*

Table S3: Sequences Analyzed

15	AY861662.1	*Oulactis orientalis*
16	AY856481.1	*Oulactis orientalis*
17	AB063314.1	*Phyllodiscus semoni*
18	AB512461.1	*Phyllodiscus semoni*
19	AY247033.1	*Sagartia rosea*
20	JQ353486.1	*Stichodactyla gigantea*
21	AJ009931.2	*Stichodactyla helianthus*
22	AJ005038.1	*Stichodactyla helianthus*
23	GQ848199.1	*Urticina crassicornis*
24	GASU01034529.1	*Acropora cervicornis*
25	GASU01040226.1	*Acropora cervicornis*
26	JR995219.1	*Acropora millepora*
27	JV077412.1	*Aiptasia pallida*
28	JV105615.1	*Aiptasia pallida*
29	JV093095.1	*Aiptasia pallida*
30	GAOL01019782.1	*Hydra magnipapillata*
31	GAOL01019715.1	*Hydra magnipapillata*
32	GAOL01014507.1	*Hydra magnipapillata*
33	GBFD01000386.1	*Hydra oligactis*
34	GBFD01006301.1	*Hydra oligactis*
35	GBFD01006728.1	*Hydra oligactis*
36	FX502289.1	*Porites australiensis*
37	FX459751.1	*Porites australiensis*
38	FX442360.1	*Porites australiensis*
39	FX440461.1	*Porites australiensis*
40	GARY01005673.1	*Stylophora pistillata*

**Hydralysins**		
1	AY178276.1	*Chlorohydra viridissima*
2	AY655142.1	*Hydra viridis*
3	AY967765.1	*Hydra viridis*
4	AY967764.1	*Hydra vulgaris*
5	XM_002159227.1	*Hydra magnipapillata*
6	XM_002158818.2	*Hydra magnipapillata*
7	GBFD01009683.1	*Hydra oligactis*
8	GAWH01023705.1	*Hydractinia symbiolongicarpus*
9	DT617282.1	*Hydra magnipapillata*

Table S3: Sequences Analyzed

10	DY447211.1	*Hydra vulgaris*
11	DT620560.1	*Hydra vulgaris*
12	DY447352.1	*Hydra vulgaris*
13	DY447829.1	*Hydra vulgaris*
14	DY447101.1	*Hydra vulgaris*
15	DY447107.1	*Hydra vulgaris*
16	DY450368.1	*Hydra vulgaris*
17	DY447499.1	*Hydra vulgaris*

**Sea anemone aerolysins**		
1	XM_001621963	*Nematostella vectensis*
2	XM_001624302	*Nematostella vectensis*
3	XM_001626030	*Nematostella vectensis*
4	XM_001629432	*Nematostella vectensis*
5	XM_001633913	*Nematostella vectensis*
6	XM_001634484	*Nematostella vectensis*
7	XM_001639868	*Nematostella vectensis*
8	XM_001639869	*Nematostella vectensis*
9	XM_001642043	*Nematostella vectensis*
10	FX474743	*Porites australiensis*
11	FX460673	*Porites australiensis*
12	GARY01026036	*Stylophora pistillata*
13	GARY01008641	*Stylophora pistillata*

**KTX Type III**		
1	FK725211.1	*Anemonia viridis*
2	FK735214.1	*Anemonia viridis*
3	FK723172.1	*Anemonia viridis*
4	FK742129.1	*Anemonia viridis*
5	FK752236.1	*Anemonia viridis*
6	FK745823.1	*Anemonia viridis*
7	FK740326.1	*Anemonia viridis*
8	FK719896.1	*Anemonia viridis*
9	FG392547.1	*Anthopleura elegantissima*
10	AB180686.1	*Antheopsis maculata*
11	HE577145.1	*Bunodosoma granulifera*

Table S3: Sequences Analyzed

12	HE577147.1	*Bunodosoma granulifera*
13	HE577146.1	*Bunodosoma granulifera*
14	HE577148.1	*Bunodosoma granulifera*

**KTX Type I**		
1	AB259113.1	*Anemonia erythraea*
2	AB595207.1	*Cryptodendrum adhaesivum*
3	AF020047.1	*Heteractis magnifica*
4	AB595208.1	*Heterodactyla hemprichi*
5	AB595204.1	*Stichodactyla gigantea*
6	AB595205.1	*Stichodactyla haddoni*
7	AB595206.1	*Stichodactyla mertensii*
8	AB595209.1	*Thalassianthus aster*
9	FK756695.1	*Anemonia viridis*
10	FK738108.1	*Anemonia viridis*
11	FK747792.1	*Anemonia viridis*
12	FK749797.1	*Anemonia viridis*
13	FC826524.1	*Metridium senile*
14	FC850067.1	*Metridium senile*
15	FC850068.1	*Metridium senile*

**NaTx**		
1	AB512763	*Thalassianthus aster*
2	AB512762	*Heterodactyla hemprichi*
3	AB362570	*Stichodactyla haddoni*
4	AB110015	*Stichodactyla gigantea*
5	AB512761	*Cryptodendrum adhaesivum*
6	EU124458	*Nematostella vectensis*
7	EU124460	*Nematostella vectensis*
8	EU124454	*Nematostella vectensis*
9	EU422969	*Nematostella vectensis*
10	EU124461	*Nematostella vectensis*
11	EU124462	*Nematostella vectensis*
12	AF130344	*Actinia equina*
13	EU124478	*Actinia equina*
14	EU124479	*Actinia equina*

Table S3: Sequences Analyzed

15	EU124480	*Actinia equina*
16	EU124481	*Actinia equina*
17	EU124482	*Actinia equina*
18	EU124483	*Actinia equina*
19	FJ418889	*Aiptasia diaphana*
20	EU124447	*Anemonia viridis*
21	EU124448	*Anemonia viridis*
22	EU124449	*Anemonia viridis*
23	EU124450	*Anemonia viridis*
24	EU124451	*Anemonia viridis*
25	EU124452	*Anemonia viridis*
26	EU124470	*Anemonia viridis*
27	EU124471	*Anemonia viridis*
28	EU124472	*Anemonia viridis*
29	EU124473	*Anemonia viridis*
30	EU124474	*Anemonia viridis*
31	EU124475	*Anemonia viridis*
32	EU124476	*Anemonia viridis*
33	EU124477	*Anemonia viridis*
34	EU919736	*Anemonia viridis*
35	AB180687	*Antheopsis maculata*
36	EU124453	*Nematostella vectensis*
37	EU124455	*Nematostella vectensis*
38	EU124456	*Nematostella vectensis*
39	EU124457	*Nematostella vectensis*
40	EU124459	*Nematostella vectensis*
41	EU124463	*Nematostella vectensis*
42	EU124464	*Nematostella vectensis*
43	EU124465	*Nematostella vectensis*
44	EU124466	*Nematostella vectensis*
45	EU124467	*Nematostella vectensis*
46	EU124468	*Nematostella vectensis*
47	EU124469	*Nematostella vectensis*
48	EU422968	*Nematostella vectensis*
49	EU422970	*Nematostella vectensis*
50	EU422971	*Nematostella vectensis*
51	EU422972	*Nematostella vectensis*

Table S3: Sequences Analyzed

52	AB110012	*Stichodactyla gigantea*
53	GU080213	*Urticina crassicornis*
54	S69403	*Calliactis parasitica*
55	FK740862	*Anemonia viridis*
56	FK756631	*Anemonia viridis*
57	FK755129	*Anemonia viridis*
58	FK730949	*Anemonia viridis*
59	FG392551	*Anthopleura elegantissima*
60	FK754096	*Anemonia viridis*
61	FK728784	*Anemonia viridis*
62	FK749453	*Anemonia viridis*
63	FC830246	*Metridium senile*
64	FC830245	*Metridium senile*

**JFTs**		
1	EF636902.1	*Chironex fleckeri*
2	AB045319.1	*Chiropsalmus quadrigatus*
3	EF636903.1	*Chironex fleckeri*
4	FJ705827.1	*Malo kingi*
5	FJ705828.1	*Malo kingi*
6	AB015878.1	*Carybdea rastonii*
7	JN695597.1	*Chironex fleckeri*
8	JN695598.1	*Chironex fleckeri*
9	AB036714.1	*Carybdea alata*
10	AB038156.1	*Carybdea alata*
11	KF583451.1	*Chironex fleckeri*
12	JQ767939.1	*Aurelia aurita*
13	GAWH01043751.1	*Hydractinia symbiolongicarpus*
14	JV127292.1	*Aiptasia pallida*
15	DN136432.2	*Hydra vulgaris*

**SCRiPs**		
1	BK006534.1	*Acropora millepora*
2	BK006535.1	*Acropora millepora*
3	BK006536.1	*Acropora millepora*
4	FJ842102.1	*Montastraea faveolata*

Table S3: Sequences Analyzed

5	FJ842103.1	*Montastraea faveolata*
7	FJ842105.1	*Montastraea faveolata*
8	FJ842106.1	*Montastraea faveolata*
9	FJ842107.1	*Montastraea faveolata*
10	BK006537.1	*Montipora capitata*
11	BK006538.1	*Montipora capitata*
12	FJ842109.1	*Montastraea faveolata*
13	GASU01030089.1	*Acropora cervicornis*
14	EZ034312.1	*Acropora millepora*
15	JR979467.1	*Acropora millepora*
16	FX437264.1	*Porites australiensis*
17	GARY01020093.1	*Stylophora pistillata*
18	DY579894.1	*Acropora millepora*
19	GO003724.1	*Acropora millepora*
20	GW196571.1	*Acropora palmata*
21	GW196827.1	*Acropora palmata*
24	DR987968.1	*Montastraea faveolata*
25	DR987884.2	*Montastraea faveolata*
26	GW249328.1	*Montastraea faveolata*
27	GW258233.1	*Montastraea faveolata*
28	GW256578.1	*Montastraea faveolata*

Scorpions

**α-NaTx**		
1	AF057554	*Mesobuthus martensii*
2	AF062563	*Mesobuthus martensii*
3	AF114025	*Mesobuthus martensii*
4	AF132974	*Mesobuthus martensii*
6	AF150009	*Mesobuthus martensii*
7	AF150011	*Mesobuthus martensii*
8	AF151796	*Mesobuthus martensii*
9	AF153693	*Mesobuthus martensii*
10	AF155363	*Mesobuthus martensii*
11	AF155364	*Mesobuthus martensii*
12	AF156168	*Mesobuthus martensii*
13	AF156169	*Mesobuthus martensii*

Table S3: Sequences Analyzed

14	AF156172	*Mesobuthus martensii*
15	AF156595	*Mesobuthus martensii*
16	AF156597	*Mesobuthus martensii*
17	AF163016	*Mesobuthus martensii*
18	AF163017	*Mesobuthus martensii*
19	AF164203	*Mesobuthus martensii*
20	AF288607	*Mesobuthus martensii*
21	AF288608	*Mesobuthus martensii*
22	AF370023	*Mesobuthus martensii*
23	AF464898	*Mesobuthus martensii*
24	AJ308439	*Androctonus australis*
25	AJ308440	*Androctonus australis*
26	AJ308441	*Androctonus australis*
27	AJ496808	*Androctonus mauretanicus*
28	AJ536595	*Androctonus amoreuxi*
29	AJ536596	*Androctonus amoreuxi*
30	AJ536597	*Androctonus amoreuxi*
31	AM159181	*Androctonus mauretanicus*
32	AM159182	*Androctonus mauretanicus*
33	AY585097	*Hottentotta judaicus*
34	DQ058408	*Mesobuthus martensii*
35	EU159278	*Lychas mucronatus*
36	EU159285	*Lychas mucronatus*
37	EU514685	*Parabuthus granulatus*
38	FN392284	*Tityus discrepans*
39	GQ454796	*Androctonus crassicauda*
40	HE585232	*Tityus cambridgei*
41	HE585233	*Tityus cambridgei*
42	HE585239	*Tityus pachyurus*
43	M27701	*Androctonus australis*
44	M27702	*Androctonus australis*
45	M27703	*Androctonus australis*
46	M27704	*Androctonus australis*
47	S69808	*Tityus serrulatus*
48	S82286	*Tityus serrulatus*
49	U28659	*Mesobuthus martensis*
50	U28660	*Buthus martensii*

Table S3: Sequences Analyzed

51	X76135	*Androctonus australis*
52	X92376	*Buthus occitanus tunetanus*
53	GT028649	*Lychas mucronatus*
54	GT028758	*Lychas mucronatus*
55	GT028835	*Lychas mucronatus*
56	GT028836	*Lychas mucronatus*
57	GT029030	*Lychas mucronatus*
58	GT029188	*Lychas mucronatus*

**β-NaTx**		
1	GALK01000007.1	*Isometroides vescus*
2	GALK01000008.1	*Isometroides vescus*
3	M27705.1	*Androctonus australis*
4	M27706.1	*Androctonus australis*
5	M27707.1	*Androctonus australis*
6	GQ454796.1	*Androctonus crassicauda*
7	AF338448.1	*Centruroides exilicauda*
8	AF338449.1	*Centruroides exilicauda*
9	AF338450.1	*Centruroides exilicauda*
10	AF338451.1	*Centruroides exilicauda*
11	AF338452.1	*Centruroides exilicauda*
12	AF338453.1	*Centruroides exilicauda*
13	AF338454.1	*Centruroides exilicauda*
14	AF338455.1	*Centruroides exilicauda*
15	AF338456.1	*Centruroides exilicauda*
16	AF338457.1	*Centruroides exilicauda*
17	AF338458.1	*Centruroides exilicauda*
18	AF338459.1	*Centruroides exilicauda*
19	AF338460.1	*Centruroides exilicauda*
20	AF338461.1	*Centruroides exilicauda*
21	AF338462.1	*Centruroides exilicauda*
22	AF338463.1	*Centruroides exilicauda*
23	AF338464.1	*Centruroides exilicauda*
24	AY649859.1	*Centruroides exilicauda*
25	AY649860.1	*Centruroides exilicauda*
26	AY649862.1	*Centruroides exilicauda*
27	AY649870.1	*Centruroides exilicauda*

Table S3: Sequences Analyzed

28	AF387171.1	*Centruroides limpidus*
29	AF488349.1	*Centruroides limpidus*
30	AF491127.1	*Centruroides limpidus*
31	AF491128.1	*Centruroides limpidus*
32	AF491129.1	*Centruroides limpidus*
33	AF491130.1	*Centruroides limpidus*
34	AF491131.1	*Centruroides limpidus*
35	AF491132.1	*Centruroides limpidus*
36	AF491133.1	*Centruroides limpidus*
37	L05060.1	*Centruroides noxius*
38	L05061.1	*Centruroides noxius*
39	L05062.1	*Centruroides noxius*
40	L05063.1	*Centruroides noxius*
41	S81093.1	*Centruroides noxius*
42	S81096.1	*Centruroides noxius*
43	Y08270.1	*Centruroides noxius*
44	AY351309.1	*Centruroides noxius*
45	AY351310.1	*Centruroides noxius*
46	AM981271.1	*Centruroides suffusus*
47	S55535.1	*Hottentotta judaicus*
48	AJ012312.1	*Buthotus judaicus*
49	AJ012313.1	*Buthotus judaicus*
50	S55538.1	*Leiurus quinquestriatus*
51	AF474983.1	*Leiurus quinquestriatus*
52	AF474984.1	*Leiurus quinquestriatus*
53	AF474985.1	*Leiurus quinquestriatus*
54	EU159276.1	*Lychas mucronatus*
55	EU159292.1	*Lychas mucronatus*
56	EU159303.1	*Lychas mucronatus*
57	Y17050.1	*Buthus martensii*
58	AF057555.1	*Buthus martensii*
59	AF062564.1	*Buthus martensii*
60	AF055672.1	*Buthus martensii*
61	AF079061.1	*Buthus martensii*
62	AF132977.1	*Buthus martensii*
63	AF156171.1	*Buthus martensii*
64	AF064821.2	*Buthus martensii*

Table S3: Sequences Analyzed

65	AF073899.2	*Buthus martensii*
66	AF241269.1	*Buthus martensii*
67	AF122003.1	*Buthus martensii*
68	AF242736.1	*Buthus martensii*
69	AF242737.1	*Buthus martensii*
70	AF272777.2	*Buthus martensii*
71	AF160868.2	*Buthus martensii*
72	AY090782.1	*Buthus martensii*
73	AF513508.1	*Buthus martensii*
74	HM233942.1	*Rhopalurus junceus*
75	HM233951.1	*Rhopalurus junceus*
76	HM233952.1	*Rhopalurus junceus*
77	HM233953.1	*Rhopalurus junceus*
78	HM233954.1	*Rhopalurus junceus*
79	HM233955.1	*Rhopalurus junceus*
80	HM233956.1	*Rhopalurus junceus*
81	JF309048.1	*Rhopalurus junceus*
82	HE585226.1	*Tityus cambridgei*
83	HE585227.1	*Tityus cambridgei*
84	HE585228.1	*Tityus cambridgei*
85	HE585231.1	*Tityus cambridgei*
86	HE585234.1	*Tityus cambridgei*
87	HE585235.1	*Tityus cambridgei*
88	HE585238.1	*Tityus cambridgei*
89	AY740693.1	*Tityus costatus*
90	DQ075226.1	*Tityus discrepans*
91	DQ075227.1	*Tityus discrepans*
92	DQ075228.1	*Tityus discrepans*
93	DQ075229.1	*Tityus discrepans*
94	DQ075230.1	*Tityus discrepans*
95	DQ075233.1	*Tityus discrepans*
96	DQ075234.1	*Tityus discrepans*
97	DQ075239.1	*Tityus discrepans*
98	DQ075242.1	*Tityus discrepans*
99	DQ075243.1	*Tityus discrepans*
100	FN392272.1	*Tityus discrepans*
101	FN392273.1	*Tityus discrepans*

Table S3: Sequences Analyzed

102	FN392274.1	*Tityus discrepans*
103	FN392275.1	*Tityus discrepans*
104	FN392276.1	*Tityus discrepans*
105	FN392277.1	*Tityus discrepans*
106	FN392279.1	*Tityus discrepans*
107	FN392280.1	*Tityus discrepans*
108	FN392281.1	*Tityus discrepans*
109	FN392282.1	*Tityus discrepans*
110	FN392283.1	*Tityus discrepans*
111	FN392285.1	*Tityus discrepans*
112	HE585240.1	*Tityus pachyurus*
113	HE585241.1	*Tityus pachyurus*
114	HE585242.1	*Tityus pachyurus*
115	HE585243.1	*Tityus pachyurus*
116	X66256.1	*Tityus serrulatus*
117	S66941.1	*Tityus serrulatus*
118	AF039599.1	*Tityus serrulatus*
119	AF039600.1	*Tityus serrulatus*
120	AY874060.1	*Tityus zulianus*
121	DQ075235.1	*Tityus zulianus*
122	DQ075236.1	*Tityus zulianus*
123	DQ075238.1	*Tityus zulianus*
124	DQ075240.1	*Tityus zulianus*
125	DQ075241.1	*Tityus zulianus*
126	GT028621	*Lychas mucronatus*
127	GT028626	*Lychas mucronatus*
128	GT028829	*Lychas mucronatus*
129	GT028830	*Lychas mucronatus*
130	GT028833	*Lychas mucronatus*
131	GT028885	*Lychas mucronatus*
132	GT028975	*Lychas mucronatus*

**Chloride**		
1	HQ288093.1	*Hottentotta judaicus*
2	AF481880.1	*Mesobuthus tamulus*
3	AF481881.1	*Mesobuthus tamulus*
4	EF445087.1	*Mesobuthus eupeus*

Table S3: Sequences Analyzed

5	EF445088.1	*Mesobuthus eupeus*
6	GU187951.1	*Mesobuthus eupeus*
7	GU187952.1	*Mesobuthus eupeus*
8	GU187953.1	*Mesobuthus eupeus*
9	HQ853233.1	*Mesobuthus eupeus*
10	AF079059.1	*Mesobuthus martensii*
11	AF135821.1	*Mesobuthus martensii*
12	AF327643.1	*Buthus martensii*
13	AF155365.1	*Buthus martensii*
14	AF159976.1	*Buthus martensii*
15	AY055475.1	*Buthus martensii*
16	AF419252.1	*Buthus martensii*
17	FJ360812.1	*Buthus occitanus israelis*
18	FJ360814.1	*Buthus occitanus israelis*
19	FJ360816.1	*Buthus occitanus israelis*
20	FJ360813.1	*Buthus occitanus israelis*
21	FJ360815.1	*Buthus occitanus israelis*

**Long KTx**		
1	GALG01000001.1	*Australobuthus xerolimniorum*
2	GALH01000007.1	*Cercophonius squama*
3	GALH01000006.1	*Cercophonius squama*
4	GALH01000005.1	*Cercophonius squama*
5	GALH01000004.1	*Cercophonius squama*
6	GALK01000011.1	*Isometroides vescus*
7	GALK01000010.1	*Isometroides vescus*
8	GALK01000009.1	*Isometroides vescus*
9	GALL01000012.1	*Lychas buchari*
10	GALI01000013.1	*Urodacus manicatus*
11	GALI01000012.1	*Urodacus manicatus*
12	GALI01000011.1	*Urodacus manicatus*
13	AF156173.1	*Buthus martensii*
14	AF155370.1	*Buthus martensii*
15	AF155371.1	*Buthus martensii*
16	AF380939.1	*Buthus martensii*
17	FJ360825.1	*Buthus occitanus*
18	FJ360829.1	*Buthus occitanus israelis*

Table S3: Sequences Analyzed

19	DQ465350.1	*Hadrurus gertschi*
20	DQ465351.1	*Hadrurus gertschi*
21	AB510468.1	*Liocheles australasiae*
22	EU163853.1	*Lychas mucronatus*
23	EU163858.1	*Lychas mucronatus*
24	EU877535.1	*Lychas mucronatus*
25	EU877536.1	*Lychas mucronatus*
26	EF442047.1	*Mesobuthus eupeus*
27	EF445076.1	*Mesobuthus eupeus*
28	EF445099.1	*Mesobuthus eupeus*
29	EF190324.1	*Mesobuthus eupeus*
30	EF190325.1	*Mesobuthus eupeus*
31	EF442062.1	*Mesobuthus eupeus*
32	EF445098.1	*Mesobuthus eupeus*
33	AF153691.1	*Mesobuthus martensii*
34	FM998749.1	*Opisthacanthus cayaporum*
35	AY423481.1	*Opistophthalmus carinatus*
36	AY423482.1	*Opistophthalmus carinatus*
37	AY423483.1	*Opistophthalmus carinatus*
38	AY423484.1	*Opistophthalmus carinatus*
39	AY423485.1	*Opistophthalmus carinatus*
40	AY423487.1	*Opistophthalmus carinatus*
41	JN315731.1	*Pandinus cavimanus*
42	JN315728.1	*Pandinus cavimanus*
43	AJ292361.1	*Pandinus imperator*
44	AY740694.1	*Tityus costatus*
45	AY740695.1	*Tityus costatus*
46	DQ465349.1	*Tityus costatus*
47	DQ465348.1	*Tityus discrepans*
48	DQ465347.1	*Tityus discrepans*
49	JX467683.1	*Tityus stigmurus*
50	DQ465345.1	*Tityus trivittatus*
51	DQ465346.1	*Tityus trivittatus*
52	EU089740.1	*Tityus trivittatus*
53	JX274244.1	*Urodacus yaschenkoi*
54	JX274245.1	*Urodacus yaschenkoi*

Table S3: Sequences Analyzed

Long KTx		
1	GALL01000013.1	*Lychas buchari*
2	GALK01000012.1	*Isometroides vescus*
3	GALL01000014.1	*Lychas buchari*
4	GALL01000015.1	*Lychas buchari*
5	GALL01000016.1	*Lychas buchari*
6	GALK01000013.1	*Isometroides vescus*
7	GALL01000017.1	*Lychas buchari*
8	GALL01000018.1	*Lychas buchari*
9	GALL01000019.1	*Lychas buchari*
10	GALK01000014.1	*Isometroides vescus*
11	S74733.1	*Androctonus australis*
12	AF151537.1	*Buthus martensii*
13	AF159974.1	*Buthus martensii*
14	AF208299.1	*Buthus martensii*
15	AF208300.1	*Buthus martensii*
16	AF247058.1	*Buthus martensii*
17	AJ277729.1	*Buthus martensii*
18	FJ360817.1	*Buthus occitanus israelis*
19	FJ360831.1	*Buthus occitanus israelis*
20	FJ360833.1	*Buthus occitanus israelis*
21	FJ360837.1	*Buthus occitanus israelis*
22	EU163848.1	*Lychas mucronatus*
23	EU163856.1	*Lychas mucronatus*
24	JX025015.1	*Mesobuthus gibbosus*
25	AF079062.1	*Mesobuthus martensii*
26	AY647145.1	*Mesobuthus martensii*
27	AY864195.1	*Mesobuthus martensii*
28	DQ132798.1	*Mesobuthus martensii*
29	JQ023128.1	*Mesobuthus martensii*
30	JQ023129.1	*Mesobuthus martensii*
31	JQ074234.1	*Mesobuthus martensii*
32	JQ074235.1	*Mesobuthus martensii*
33	AY740685.1	*Tityus costatus*
34	FJ360826.1	*Buthus occitanus israelis*
35	FJ360832.1	*Buthus occitanus israelis*
36	JN872636.1	*Heterometrus laoticus*

Table S3: Sequences Analyzed

37	HQ288192.1	*Hottentotta judaicus*
38	EU163855.1	*Lychas mucronatus*
39	AF276623.1	*Mesobuthus eupeus*
40	GU327370.1	*Mesobuthus eupeus*
41	JF719812.1	*Mesobuthus eupeus*
42	JX025013.1	*Mesobuthus gibbosus*
43	JX025014.1	*Mesobuthus gibbosus*
44	AF249746.1	*Mesobuthus martensii*
45	AF534113.1	*Mesobuthus martensii*
46	AY129234.1	*Mesobuthus martensii*
47	AF541980.1	*Mesobuthus martensii*
48	AF153692.1	*Mesobuthus martensii*
49	AF154634.1	*Mesobuthus martensii*
50	AF520910.1	*Mesobuthus martensii*
51	JF719810.1	*Mesobuthus martensii*
52	JQ074233.1	*Mesobuthus martensii*
53	U79579.1	*Pandinus imperator*
54	AJ277726.1	*Mesobuthus martensii*
55	AJ277727.1	*Mesobuthus martensii*
56	AF159975.1	*Mesobuthus martensii*
57	AF135817.1	*Mesobuthus martensii*
58	AF325113.1	*Mesobuthus martensii*
59	AJ277728.1	*Mesobuthus martensii*
60	AF135818.1	*Mesobuthus martensii*
61	AY730620.1	*Mesobuthus martensii*
62	HQ650828.1	*Mesobuthus martensii*
63	HQ650832.1	*Mesobuthus martensii*
64	HQ650829.1	*Mesobuthus martensii*
65	HQ650831.1	*Mesobuthus martensii*
66	HQ650830.1	*Mesobuthus martensii*
67	FJ360824.1	*Buthus occitanus israelis*
68	AJ277730.1	*Mesobuthus martensii*
69	HQ650827.1	*Mesobuthus eupeus*
70	JQ781066.1	*Mesobuthus martensii*
71	HQ650826.1	*Mesobuthus eupeus*
72	JQ798184.1	*Mesobuthus martensii*
73	AF295594.1	*Mesobuthus martensii*

Table S3: Sequences Analyzed

74	JQ781065.1	*Mesobuthus martensii*
75	AF247057.1	*Mesobuthus martensii*
76	AY125328.1	*Mesobuthus martensii*
77	HQ288111.1	*Hottentotta judaicus*
78	JQ781064.1	*Mesobuthus eupeus*
79	AY125327.1	*Mesobuthus martensii*
80	FJ360823.1	*Buthus occitanus israelis*

**Anionic**		
1	GALH01000001.1	*Cercophonius squama*
2	GALK01000002.1	*Isometroides vescus*
3	GALL01000002.1	*Lychas buchari*
4	GALL01000001.1	*Lychas buchari*
5	GALI01000002.1	*Urodacus manicatus*
6	FJ360842.1	*Buthus occitanus israelis*
7	EF442043.1	*Mesobuthus eupeus*
8	EF442044.1	*Mesobuthus eupeus*
9	EF445058.1	*Mesobuthus eupeus*
10	EF445068.1	*Mesobuthus eupeus*
11	EF445090.1	*Mesobuthus eupeus*
12	AF150012.1	*Mesobuthus martensii*
13	AY648240.1	*Mesobuthus martensii*
14	JN315714.1	*Pandinus cavimanus*
15	AY740689.1	*Tityus costatus*
16	AY740690.1	*Tityus costatus*
17	AY740691.1	*Tityus costatus*
18	AY740692.1	*Tityus costatus*
19	FN392269.1	*Tityus discrepans*
20	GT028723.1	*Lychas mucronatus*

**Linear Peptides**		
1	c21	*Chaerilus tryznai*
2	c2	*Chaerilus tricostatus*
3	c30	*Chaerilus tricostatus*
4	c10	*Chaerilus tricostatus*
5	c65	*Chaerilus tryznai*

Table S3: Sequences Analyzed

6	c45	*Chaerilus tricostatus*
7	c64	*Chaerilus tryznai*
8	c54	*Chaerilus tricostatus*
9	c13	*Chaerilus tryznai*
10	c32	*Chaerilus tryznai*
11	GALH01000010.1	*Cercophonius squama*
12	GALH01000009.1	*Cercophonius squama*
13	GALH01000008.1	*Cercophonius squama*
14	GALI01000005.1	*Urodacus manicatus*
15	GALI01000004.1	*Urodacus manicatus*
16	GALI01000007.1	*Urodacus manicatus*
17	GALI01000006.1	*Urodacus manicatus*
18	GALI01000003.1	*Urodacus manicatus*
19	FJ360839.1	*Buthus occitanus israelis*
20	EF442055.1	*Mesobuthus eupeus*
21	EF445077.1	*Mesobuthus eupeus*
22	AF151795.1	*Buthus martensii*
23	AF150010.1	*Buthus martensii*
24	AY323830.1	*Mesobuthus martensii*
25	FM998743.1	*Opisthacanthus cayaporum*
26	FM998744.1	*Opisthacanthus cayaporum*
27	AF397895.1	*Opisthacanthus madagascariensis*
28	AY050522.1	*Opisthacanthus madagascariensis*
29	JN315745.1	*Pandinus cavimanus*
30	JX274240.1	*Urodacus yaschenkoi*
31	JX274241.1	*Urodacus yaschenkoi*
32	JX274242.1	*Urodacus yaschenkoi*
33	JQ086325.1	*Vaejovis mexicanus*
34	JQ086326.1	*Vaejovis mexicanus*
35	JQ086327.1	*Vaejovis subcristatus*
36	JQ086328.1	*Vaejovis subcristatus*
37	EF445102.1	*Mesobuthus eupeus*
38	EL698901.1	*Hadrurus gertschi*
39	FD664386.1	*Scorpiops tibetanus*

**AMPs**		
1	GALK01000015.1	*Isometroides vescus*

Table S3: Sequences Analyzed

2	GALL01000021.1	*Lychas buchari*
3	GALL01000022.1	*Lychas buchari*
4	c3	*Chaerilus tricostatus*
5	c5	*Chaerilus tricostatus*
6	c7	*Chaerilus tricostatus*
7	FR821613.1	*Androctonus amoreuxi*
8	FR821614.1	*Androctonus amoreuxi*
9	JN172934.1	*Chaerilus tricostatus*
10	FJ750949.1	*Isometrus maculatus*
11	EU163896.1	*Lychas mucronatus*
12	EU669864.1	*Lychas mucronatus*
13	DQ988344.1	*Mesobuthus eupeus*
14	EF445092.1	*Mesobuthus eupeus*
16	EF445103.1	*Mesobuthus eupeus*
17	HM235675.1	*Mesobuthus eupeus*
18	KC108907.1	*Mesobuthus eupeus*
19	AF159979.1	*Mesobuthus martensii*
20	AF543048.1	*Mesobuthus martensii*
21	AY700566.1	*Mesobuthus martensii*
22	AY729077.1	*Mesobuthus martensii*
23	DQ988345.1	*Mesobuthus martensii*
24	DQ988346.1	*Mesobuthus martensii*
25	DQ988347.1	*Mesobuthus martensii*
26	DQ988348.1	*Mesobuthus martensii*
27	EU158251.1	*Mesobuthus martensii*
28	HM235676.1	*Mesobuthus martensii*
29	HM235677.1	*Mesobuthus martensii*
30	HM235678.1	*Mesobuthus martensii*
31	FM998745.1	*Opisthacanthus cayaporum*
32	FM998756.1	*Opisthacanthus cayaporum*
33	FM998757.1	*Opisthacanthus cayaporum*
34	JN315744.1	*Pandinus cavimanus*
35	AY740686.1	*Tityus costatus*
36	AY740687.1	*Tityus costatus*
37	AY740688.1	*Tityus costatus*
38	GT028819.1	*Tityus costatus*
39	GT028820.1	*Tityus costatus*

Table S3: Sequences Analyzed

40	GT028840.1	*Tityus costatus*
41	GT028857.1	*Tityus costatus*
42	GT029142.1	*Tityus costatus*

**Bradykinin Potentiating Peptide**		
1	GALK01000016.1	*Isometroides vescus*
2	GALK01000017.1	*Isometroides vescus*
3	c73	*Chaerilus tricostatus*
4	AF145952.1	*Buthus martensii*
5	AF145953.1	*Buthus martensii*
6	FJ360843.1	*Buthus occitanus israelis*
7	EU163889.1	*Lychas mucronatus*
8	EU163895.1	*Lychas mucronatus*
9	EF442059.1	*Mesobuthus eupeus*
10	AF146744.1	*Mesobuthus martensii*
11	FM998759.1	*Opisthacanthus cayaporum*
12	FN392268.1	*Tityus discrepans*
13	JX274243.1	*Urodacus yaschenkoi*
14	HM102352.1	*Vaejovis mexicanus*
15	EL698903.1	*Hadrurus gertschi*
16	GT028786.1	*Lychas mucronatus*

**Glycine-rich Peptides**		
1	GALL01000025.1	*Lychas buchari*
2	GALI01000014.1	*Urodacus manicatus*
3	GALL01000026.1	*Lychas buchari*
4	GALL01000024.1	*Lychas buchari*
5	GALL01000027.1	*Lychas buchari*
6	GT029007.1	*Lychas mucronatus*

**SV-SVCs**		
1	GALH01000011.1	*Cercophonius squama*
2	GALH01000012.1	*Cercophonius squama*
3	GALL01000029.1	*Lychas buchari*
4	GALL01000030.1	*Lychas buchari*
5	GALL01000028.1	*Lychas buchari*

Table S3: Sequences Analyzed

6	KC140563.1	*Heterometrus spinifer*
7	HQ288168.1	*Hottentotta judaicus*
8	HQ288209.1	*Hottentotta judaicus*
9	AF155366.1	*Buthus martensii*
10	DQ352540.1	*Mesobuthus martensii*
11	EF063687.1	*Mesobuthus martensii*
12	FM998753.1	*Opisthacanthus cayaporum*
13	JN315721.1	*Pandinus cavimanus*
14	JX274246.1	*Urodacus yaschenkoi*
15	JX274247.1	*Urodacus yaschenkoi*
16	JX274250.1	*Urodacus yaschenkoi*
17	FM998779.1	*Opisthacanthus cayaporum*
18	EF445086.1	*Mesobuthus eupeus*
19	GT029212.1	*Lychas mucronatus*

**ICKs**		
1	FJ360811.1	*Buthus occitanus israelis*
2	EU496812.1	*Hadrurus gertschi*
3	HQ288104.1	*Hottentotta judaicus*
4	EF445100.1	*Mesobuthus eupeus*
5	EF445101.1	*Mesobuthus eupeus*
6	DQ120790.1	*Mesobuthus martensii*
7	DQ206446.1	*Mesobuthus martensii*
8	DQ201205.1	*Mesobuthus martensii*
9	AF419253.2	*Mesobuthus martensii*
10	DQ272503.1	*Mesobuthus martensii*
11	AY225784.1	*Opistophthalmus carinatus*
12	GT029194.1	*Lychas mucronatus*
13	JX274248.1	*Urodacus yaschenkoi*
14	SRR398954.551300	*Centruroides noxius*
15	SRR398954.623832	*Centruroides noxius*
16	SRR402040.190746	*Centruroides noxius*
17	SRR402040.191590	*Centruroides noxius*
18	SRR402040.194225	*Centruroides noxius*
19	SRR402040.207425	*Centruroides noxius*
20	SRR402040.213790	*Centruroides noxius*
21	SRR402040.218195	*Centruroides noxius*

Table S3: Sequences Analyzed

22	SRR402040.220248	*Centruroides noxius*
23	SRR402040.223783	*Centruroides noxius*
24	SRR402040.243530	*Centruroides noxius*
25	SRR402040.251500	*Centruroides noxius*
26	SRR402040.254485	*Centruroides noxius*
27	SRR402040.254935	*Centruroides noxius*
28	SRR402040.254991	*Centruroides noxius*

DDH		
1	GALI01000015.1	*Urodacus manicatus*
2	GALI01000016.1	*Urodacus manicatus*
3	GALI01000017.1	*Urodacus manicatus*
4	U1LITXLw1a	*Liocheles waigiensis*
5	FM998752.1	*Opisthacanthus cayaporum*

Coleoids

Serine Protease		
1	GAGQ01000003.1	*Sepia latimanus*
2	GAGP01000006.1	*Sepia pharaonis*
3	GAGN01000013.1	*Sepioteuthis australis*
4	GBHH01001106.1	*Euprymna scolopes*
5	GAGI01000019.1	*Abdopus aculeatus*
6	GAGL01000008.1	*Octopus cyanea*
7	GAGL01000009.1	*Octopus cyanea*
8	GAGI01000008.1	*Abdopus aculeatus*
9	GAGO01000005.1	*Octopus kaurna*
10	GAGJ01000006.1	*Hapalochlaena maculosa*
11	GAGM01000003.1	*Pareledone turqueti*
12	GAGL01000010.1	*Octopus cyanea*
13	GAGI01000024.1	*Abdopus aculeatus*
14	GAGI01000015.1	*Abdopus aculeatus*
15	GAGI01000014.1	*Abdopus aculeatus*
16	GAGJ01000007.1	*Hapalochlaena maculosa*
17	GAGL01000007.1	*Octopus cyanea*
18	GAGQ01000004.1	*Sepia latimanus*

Table S3: Sequences Analyzed

19	GAGI01000023.1	*Abdopus aculeatus*
20	GAGL01000006.1	*Octopus cyanea*
21	GAGI01000020.1	*Abdopus aculeatus*
22	GAGI01000016.1	*Abdopus aculeatus*
23	GAGI01000013.1	*Abdopus aculeatus*
24	GAGI01000025.1	*Abdopus aculeatus*
25	GAGP01000007.1	*Sepia pharaonis*
26	GAGM01000002.1	*Pareledone turqueti*
27	GAGQ01000002.1	*Sepia latimanus*
28	GAGI01000022.1	*Abdopus aculeatus*
29	GAGP01000005.1	*Sepia pharaonis*
30	GAGN01000012.1	*Sepioteuthis australis*
31	GAGK01000006.1	*Loliolus noctiluca*
32	GAGN01000010.1	*Sepioteuthis australis*
33	GAGN01000011.1	*Euprymna scolopes*
34	GBHH01004618.1	*Euprymna scolopes*
35	GAGI01000012.1	*Abdopus aculeatus*
36	GAGI01000018.1	*Abdopus aculeatus*
37	GAGI01000007.1	*Abdopus aculeatus*
38	GAGI01000009.1	*Abdopus aculeatus*
39	GAGI01000021.1	*Abdopus aculeatus*
40	GBHH01001371.1	*Euprymna scolopes*
41	GAGI01000017.1	*Abdopus aculeatus*
42	GAGI01000010.1	*Abdopus aculeatus*
43	GAGO01000004.1	*Octopus kaurna*
44	GAGI01000011.1	*Abdopus aculeatus*
45	JR455425.1	*Octopus vulgaris*
46	JR440367.1	*Octopus vulgaris*
47	JR449946.1	*Octopus vulgaris*
48	JR448570.1	*Octopus vulgaris*
49	JR465628.1	*Octopus vulgaris*

**Pacifastin**		
1	GAGP01000002.1	*Sepia pharaonis*
2	GAGO01000003.1	*Octopus kaurna*
3	GAGN01000002.1	*Sepioteuthis australis*
4	GAGL01000002.1	*Octopus cyanea*

Table S3: Sequences Analyzed

5	GAGL01000003.1	*Octopus cyanea*
6	GAGL01000004.1	*Octopus cyanea*
7	GAGK01000002.1	*Loliolus noctiluca*
8	GAGJ01000004.1	*Hapalochlaena maculosa*
9	GAGI01000002.1	*Abdopus aculeatus*
10	GAGI01000003.1	*Abdopus aculeatus*
11	GAGI01000004.1	*Abdopus aculeatus*

**CAP**		
1	JR439728.1	*Octopus vulgaris*
2	GBHH01002488.1	*Euprymna scolopes*
3	JR436131.1	*Octopus vulgaris*
4	JR447835.1	*Octopus vulgaris*
5	EU790590.1	*Hapalochlaena maculosa*

**PLA2**		
1	GAGN01000003.1	*Sepioteuthis australis*
2	GAGK01000003.1	*Loliolus noctiluca*
3	EU790608.1	*Sepia latimanus*
4	GAGP01000003.1	*Sepia pharaonis*
5	GAGP00000000.1	*Sepia pharaonis*

**Kallikreins**		
1	GU441497.1	*Celestus warreni*
2	GU441498.1	*Celestus warreni*
3	GU441502.1	*Celestus warreni*
4	GU441495.1	*Gerrhonotus infernalis*
5	GU441496.1	*Gerrhonotus infernalis*
6	GU441503.1	*Gerrhonotus infernalis*
7	EU790962.1	*Heloderma suspectum cinctum*
8	EU790963.1	*Heloderma suspectum cinctum*
9	EU195456.1	*Varanus komodoensis*
10	EU195457.1	*Varanus komodoensis*
11	EU195458.1	*Varanus komodoensis*
12	DQ139926.1	*Varanus varius*
13	HM437246.1	*Heloderma horridum*

Table S3: Sequences Analyzed

14	DQ139911.1	*Varanus acanthurus*
15	DQ139912.1	*Varanus acanthurus*
16	DQ139913.1	*Varanus acanthurus*
17	DQ139914.1	*Varanus acanthurus*
18	GU441504.1	*Varanus eremius*
19	GU441494.1	*Varanus giganteus*
20	GU441480.1	*Varanus gilleni*
21	GU441481.1	*Varanus glauerti*
22	GU441487.1	*Varanus glauerti*
23	GU441478.1	*Varanus gouldii*
24	GU441483.1	*Varanus gouldii*
25	GU441485.1	*Varanus gouldii*
26	GU441489.1	*Varanus gouldii*
27	GU441492.1	*Varanus gouldii*
28	GU441479.1	*Varanus indicus*
29	GU441482.1	*Varanus indicus*
30	GU441484.1	*Varanus mertensi*
31	DQ139915.1	*Varanus mitchelli*
32	DQ139916.1	*Varanus mitchelli*
33	DQ139917.1	*Varanus mitchelli*
34	DQ139918.1	*Varanus mitchelli*
35	GU441490.1	*Varanus panoptes*
36	GU441493.1	*Varanus panoptes*
37	GU441500.1	*Varanus panoptes*
38	GU441501.1	*Varanus panoptes*
39	DQ139924.1	*Varanus panoptes*
40	DQ139925.1	*Varanus panoptes*
41	DQ184481.1	*Varanus panoptes*
42	GU441477.1	*Varanus scalaris*
43	GU441486.1	*Varanus scalaris*
44	GU441491.1	*Varanus scalaris*
45	GU441499.1	*Varanus scalaris*
46	GU441488.1	*Varanus tristis*

Toxicofera Lizards

**CRiSPs**

Table S3: Sequences Analyzed

1	GU441473.1	*Celestus warreni*
2	GU441472.1	*Gerrhonotus infernalis*
3	U13619.1	*Heloderma horridum*
4	EU790958.1	*Heloderma suspectum*
5	GU441462.1	*Ophisaurus apodus*
6	DQ139883.1	*Varanus acanthurus*
7	DQ139884.1	*Varanus acanthurus*
8	DQ139885.1	*Varanus acanthurus*
9	DQ139886.1	*Varanus acanthurus*
10	DQ139887.1	*Varanus acanthurus*
11	DQ139888.1	*Varanus acanthurus*
12	GU441466.1	*Varanus giganteus*
13	GU441467.1	*Varanus gilleni*
14	GU441465.1	*Varanus gouldii*
15	GU441469.1	*Varanus indicus*
16	GU441470.1	*Varanus indicus*
17	EU195455.1	*Varanus komodoensis*
18	GU441463.1	*Varanus mertensi*
19	GU441464.1	*Varanus panoptes*
20	GU441471.1	*Varanus scalaris*
21	GU441468.1	*Varanus tristis*
22	DQ139903.1	*Varanus varius*
23	DQ139904.1	*Varanus varius*

**Crotamine**		
1	JX467135.1	*Varanus tristis*
2	JX467134.1	*Varanus glauerti*
3	JX467133.1	*Varanus glauerti*
4	JX467132.1	*Varanus glauerti*
5	JX467131.1	*Varanus glauerti*

**PLA2**		
1	GU441525.1	*Celestus warreni*
2	EU790968.1	*Heloderma suspectum*
3	EU790967.1	*Heloderma suspectum*
4	EU195460.1	*Varanus komodoensis*

Table S3: Sequences Analyzed

5	DQ139930.1	*Varanus varius*

Natriuretic peptides		
1	GU441511.1	*Celestus warreni*
2	GU441510.1	*Gerrhonotus infernalis*
3	FN555307.1	*Heloderma suspectum*
4	FN555308.1	*Heloderma suspectum*
5	EU790965.1	*Heloderma suspectum*
6	EU195461.1	*Varanus komodoensis*
7	DQ139927.1	*Varanus varius*
8	FR666707.1	*Heloderma horridum*
9	GU441507.1	*Varanus glauerti*
10	GU441509.1	*Varanus glauerti*
11	GU441508.1	*Varanus scalaris*

NGF		
1	GU432718.1	*Anniella pulchra*
2	GU432725.1	*Lanthanotus borneensis*
3	GU432719.1	*Celestus enneagrammus*
4	GU432720.1	*Elgaria multicarinata*
5	GU432722.1	*Heloderma suspectum*
6	EU790966.1	*Heloderma suspectum*
7	GU432721.1	*Heloderma horridum*
8	JN880631.1	*Ophisaurus ventralis*
9	GU432717.1	*Ophisaurus apodus*
10	GU432723.1	*Shinisaurus crocodilurus*
11	GU432726.1	*Varanus acanthurus*
12	GU432727.1	*Varanus exanthematicus*
13	EU437981.1	*Varanus salvator*
14	GU432724.1	*Xenosaurus grandis*
15	EU437980.1	*Xenosaurus platyceps*
16	c474	*Abronia gramminea*
17	c785	*Abronia gramminea*
18	c662	*Heloderma suspectum*

Table S3: Sequences Analyzed

Cone Snails

Conantokin		
1	HIJWH8M02JZ00K_4_1	*Conus geographus*
2	HIJWH8M02JZY6C_5_1	*Conus geographus*
3	HIJWH8M02JWH4L_4_1	*Conus geographus*
4	HIJWH8M02JZTPJ_3_1	*Conus geographus*
5	HIJWH8M02JYV8Q_5_1	*Conus geographus*
6	HIJWH8M02JQSU9_2_1	*Conus geographus*
7	HIJWH8M02JZL2R_3_1	*Conus geographus*
8	HIJWH8M02JVRF5_5_1	*Conus geographus*
9	HIJWH8M02JWHZU_5_1	*Conus geographus*
10	HIJWH8M02JUJHO_6_1	*Conus geographus*
11	HIJWH8M02IWKVZ_2_1	*Conus geographus*
12	HIJWH8M02JN0T3_1_1	*Conus geographus*
13	HIJWH8M02J2J5H_3_1	*Conus geographus*
14	HIJWH8M02JW2O3_2_1	*Conus geographus*
15	HIJWH8M02JUPBO_2_1	*Conus geographus*
16	HIJWH8M02JQHQY_3_1	*Conus geographus*
17	HIJWH8M02JN798_1_1	*Conus geographus*
18	HIJWH8M02JYPH7_2_1	*Conus geographus*
19	HIJWH8M02JHGRC_3_1	*Conus geographus*
20	HIJWH8M02JQ65A_2_1	*Conus geographus*
21	HIJWH8M02JH9RL_3_1	*Conus geographus*
22	HIJWH8M02IRS71_1_1	*Conus geographus*
23	HIJWH8M02HY32M_2_1	*Conus geographus*
24	HIJWH8M02IWI7P_5_1	*Conus geographus*
25	HIJWH8M02JT0TA_2_1	*Conus geographus*
26	HIJWH8M02JOZ5U_2_1	*Conus geographus*
27	HIJWH8M02JPBU0_3_1	*Conus geographus*
28	HIJWH8M01EX5AU_3_1	*Conus geographus*
29	HIJWH8M02HOXYU_3_1	*Conus geographus*
30	HIJWH8M02JL4BI_5_1	*Conus geographus*
31	HIJWH8M02IRZ46_2_1	*Conus geographus*
32	HIJWH8M02H7N7W_2_1	*Conus geographus*
33	HIJWH8M02ISVZM_6_1	*Conus geographus*
34	HIJWH8M02IXJV0_2_1	*Conus geographus*
35	HIJWH8M02IMO1R_5_1	*Conus geographus*

Table S3: Sequences Analyzed

36	HIJWH8M02JTUOA_1_1	*Conus geographus*
37	HIJWH8M02JZZC9_2_1	*Conus geographus*
38	HIJWH8M02JWYBC_2_1	*Conus geographus*
39	HIJWH8M02IYO90_3_1	*Conus geographus*
40	HIJWH8M02FPBPC_5_1	*Conus geographus*
41	HIJWH8M02JLQ01_5_1	*Conus geographus*
42	HIJWH8M02G9XU3_5_1	*Conus geographus*
43	HIJWH8M02JJM5X_6_1	*Conus geographus*
44	HIJWH8M02JOWWR_5_1	*Conus geographus*
45	HIJWH8M02GX0E3_4_1	*Conus geographus*
46	HIJWH8M02JPKQ0_2_1	*Conus geographus*
47	HIJWH8M02ITOJZ_2_1	*Conus geographus*
48	HIJWH8M02JQ5TT_1_1	*Conus geographus*
49	HIJWH8M02JW6I0_2_1	*Conus geographus*
50	HIJWH8M02JJ3ZQ_6_1	*Conus geographus*
51	HIJWH8M02F4OO0_3_1	*Conus geographus*
52	HIJWH8M02IYKWA_6_1	*Conus geographus*
53	HIJWH8M02H5RUX_2_1	*Conus geographus*
54	HIJWH8M02HH3YM_3_1	*Conus geographus*
55	HIJWH8M01DANIJ_4_1	*Conus geographus*
56	HIJWH8M02J3VJZ_6_1	*Conus geographus*
57	HIJWH8M02JE1Y3_4_1	*Conus geographus*
58	HIJWH8M02GVNGD_4_1	*Conus geographus*
59	HIJWH8M02H2ZHK_3_1	*Conus geographus*
60	HIJWH8M02J6W77_6_1	*Conus geographus*
61	HIJWH8M02JZ6JI_1_1	*Conus geographus*
62	HIJWH8M02HZDVQ_6_1	*Conus geographus*
63	HIJWH8M02HOKSN_3_1	*Conus geographus*
64	HIJWH8M02IHAMG_6_1	*Conus geographus*
65	HIJWH8M02GVQ61_1_1	*Conus geographus*
66	HIJWH8M02HAQ0W_3_1	*Conus geographus*
67	HIJWH8M02IFQWF_2_1	*Conus geographus*
68	HIJWH8M02IRQFU_3_1	*Conus geographus*
69	HIJWH8M02HLE0L_1_1	*Conus geographus*
70	HIJWH8M02GSJGP_3_1	*Conus geographus*
71	HIJWH8M02FTO4C_5_1	*Conus geographus*
72	HIJWH8M02JSZZ4_4_1	*Conus geographus*

Table S3: Sequences Analyzed

73	HIJWH8M02IHW37_6_1	*Conus geographus*
74	HIJWH8M02IRM9D_2_2	*Conus geographus*
75	HIJWH8M02J04CO_3_1	*Conus geographus*
76	HIJWH8M02HJ2MK_2_1	*Conus geographus*
77	HIJWH8M02FSV8H_6_1	*Conus geographus*
78	HIJWH8M01BM18Z_1_1	*Conus geographus*
79	HIJWH8M02HBRBA_5_1	*Conus geographus*
80	HIJWH8M01BKO42_6_1	*Conus geographus*
81	HIJWH8M02JTQ9G_5_1	*Conus geographus*
82	HIJWH8M02JKK6W_6_1	*Conus geographus*
83	HIJWH8M02I78GB_6_1	*Conus geographus*
84	HIJWH8M02HR5GC_3_1	*Conus geographus*
85	HIJWH8M02JX5IS_5_1	*Conus geographus*
86	HIJWH8M01EI9RD_3_1	*Conus geographus*
87	HIJWH8M02G729P_6_1	*Conus geographus*
88	HIJWH8M02IGXAV_2_1	*Conus geographus*
89	HIJWH8M02GP176_2_1	*Conus geographus*
90	HIJWH8M02ILRS1_6_1	*Conus geographus*
91	HIJWH8M01D66I0_2_1	*Conus geographus*
92	HIJWH8M02GFTWE_5_1	*Conus geographus*
93	HIJWH8M01EIB0P_6_1	*Conus geographus*
94	HIJWH8M02I2LYI_4_1	*Conus geographus*
95	HIJWH8M02HWIXJ_5_1	*Conus geographus*
96	HIJWH8M02JB0AJ_3_1	*Conus geographus*
97	HIJWH8M02JMAQX_1_1	*Conus geographus*
98	HIJWH8M01CMI5D_4_1	*Conus geographus*
99	HIJWH8M02JTZ3Y_6_1	*Conus geographus*
100	HIJWH8M02H2DZT_5_1	*Conus geographus*
101	HIJWH8M02GICRS_2_1	*Conus geographus*
102	HIJWH8M02J2G48_2_1	*Conus geographus*
103	HIJWH8M02GE1AU_2_1	*Conus geographus*
104	HIJWH8M02GZRUO_2_1	*Conus geographus*
105	HIJWH8M02HLFBP_1_1	*Conus geographus*
106	HIJWH8M02ILHN9_2_1	*Conus geographus*
107	HIJWH8M01DHTJJ_3_1	*Conus geographus*
108	HIJWH8M02F2DCK_2_1	*Conus geographus*
109	HIJWH8M01ESM17_3_1	*Conus geographus*

Table S3: Sequences Analyzed

110	HIJWH8M02F1XM9_3_1	*Conus geographus*
111	HIJWH8M02HOT76_4_1	*Conus geographus*
112	HIJWH8M02IDTIP_2_1	*Conus geographus*
113	HIJWH8M01C1FRS_3_1	*Conus geographus*
114	HIJWH8M02JPJDK_6_1	*Conus geographus*
115	HIJWH8M02GTGOJ_2_1	*Conus geographus*
116	HIJWH8M01D8QNA_5_1	*Conus geographus*
117	HIJWH8M02IEIA8_1_1	*Conus geographus*
118	HIJWH8M01D7CL7_2_1	*Conus geographus*
119	HIJWH8M01CYB9E_3_1	*Conus geographus*
120	HIJWH8M02IQI30_3_1	*Conus geographus*
121	HIJWH8M01DL110_2_1	*Conus geographus*
122	HIJWH8M02H5V8K_3_1	*Conus geographus*
123	HIJWH8M02HM8RI_5_1	*Conus geographus*
124	HIJWH8M02IM2RC_5_1	*Conus geographus*
125	HIJWH8M02ISGNS_4_1	*Conus geographus*
126	HIJWH8M02F239K_5_1	*Conus geographus*
127	HIJWH8M02JLE5E_3_1	*Conus geographus*
128	HIJWH8M02FMRD3_4_1	*Conus geographus*
129	HIJWH8M02H5UH1_4_1	*Conus geographus*
130	HIJWH8M01DOZ67_4_1	*Conus geographus*
131	HIJWH8M02HBM6Z_1_1	*Conus geographus*
132	HIJWH8M02I4655_2_1	*Conus geographus*
133	HIJWH8M02JAFJP_3_1	*Conus geographus*
134	HIJWH8M02HZZ9W_6_1	*Conus geographus*
135	HIJWH8M02JX1HW_2_1	*Conus geographus*
136	HIJWH8M02GP32N_1_1	*Conus geographus*
137	HIJWH8M01BC1XD_4_1	*Conus geographus*
138	HIJWH8M02JGYKN_2_1	*Conus geographus*
139	HIJWH8M02GIEV3_2_1	*Conus geographus*
140	HIJWH8M02IJ1ZR_5_1	*Conus geographus*
141	HIJWH8M02JB9ID_3_1	*Conus geographus*
142	HIJWH8M02I8SKC_1_1	*Conus geographus*
143	HIJWH8M02HVDXE_2_1	*Conus geographus*
144	HIJWH8M02IRN0Y_2_1	*Conus geographus*
145	HIJWH8M02I1VJW_2_1	*Conus geographus*
146	HIJWH8M02ILAUJ_5_1	*Conus geographus*

Table S3: Sequences Analyzed

147	HIJWH8M01DUR0U_1_1	*Conus geographus*

**Conantokin**		
1	HIJWH8M02JZ53H_4_1	*Conus geographus*
2	HIJWH8M02JUHE8_6_1	*Conus geographus*
3	HIJWH8M02JVFOI_2_1	*Conus geographus*
4	HIJWH8M02JMP09_6_1	*Conus geographus*
5	HIJWH8M02JYX5W_2_1	*Conus geographus*
6	HIJWH8M02JDOQ5_2_1	*Conus geographus*
7	HIJWH8M02IMK9O_1_1	*Conus geographus*
8	HIJWH8M02G92XD_2_1	*Conus geographus*
9	HIJWH8M02I1UQD_3_1	*Conus geographus*
10	HIJWH8M02JYCIV_4_1	*Conus geographus*
11	HIJWH8M01D23V2_3_1	*Conus geographus*
12	HIJWH8M02JF6DY_2_1	*Conus geographus*

**Conkunitzin**		
1	HIJWH8M02JY6LH_4_1	*Conus geographus*
2	HIJWH8M02HWGOY_4_1	*Conus geographus*
3	HIJWH8M02II3NV_5_1	*Conus geographus*
4	HIJWH8M02HWWIH_1_1	*Conus geographus*
5	HIJWH8M02FOIG3_5_1	*Conus geographus*

**Superfamily A**		
1	HIJWH8M02JYCPK_6_1	*Conus geographus*
2	HIJWH8M02JZNK0_5_1	*Conus geographus*
3	HIJWH8M02JZTN7_1_1	*Conus geographus*
4	HIJWH8M02JY1EC_2_1	*Conus geographus*
5	HIJWH8M02JYVSL_5_1	*Conus geographus*
6	HIJWH8M02JXOLN_1_1	*Conus geographus*
7	HIJWH8M02JSQEX_1_1	*Conus geographus*
8	HIJWH8M02JM6FO_2_1	*Conus geographus*
9	HIJWH8M02IWRMV_1_1	*Conus geographus*
10	HIJWH8M02JDP32_2_1	*Conus geographus*
11	HIJWH8M01EXBCF_1_1	*Conus geographus*
12	HIJWH8M02JCVT0_2_1	*Conus geographus*

Table S3: Sequences Analyzed

13	HIJWH8M02I43VE_1_1	*Conus geographus*
14	HIJWH8M02HS8QE_2_1	*Conus geographus*
15	HIJWH8M02JY9KT_5_1	*Conus geographus*
16	HIJWH8M02JMHLB_5_1	*Conus geographus*
17	HIJWH8M01EXA2I_1_1	*Conus geographus*
18	HIJWH8M02I5SVC_4_1	*Conus geographus*
19	HIJWH8M02G4ZHB_3_1	*Conus geographus*
20	HIJWH8M02HA66L_3_1	*Conus geographus*
21	HIJWH8M01CGKAI_6_1	*Conus geographus*
22	HIJWH8M01D7WOQ_5_1	*Conus geographus*
23	HIJWH8M01DYN2M_2_1	*Conus geographus*
24	HIJWH8M02JCRL3_3_1	*Conus geographus*
25	HIJWH8M02F5AIA_6_1	*Conus geographus*

**Superfamily M**		
1	HIJWH8M02JZZ1H_3_1	*Conus geographus*
2	HIJWH8M02JZUEX_1_1	*Conus geographus*
3	HIJWH8M02I9APV_2_1	*Conus geographus*
4	HIJWH8M02JNO2W_1_1	*Conus geographus*
5	HIJWH8M02IFO8Q_2_1	*Conus geographus*
6	HIJWH8M02GHNNA_3_1	*Conus geographus*
7	HIJWH8M02IW7O0_2_1	*Conus geographus*
8	HIJWH8M02IL4VH_1_1	*Conus geographus*
9	HIJWH8M01EVIQZ_1_1	*Conus geographus*
10	HIJWH8M02JPCE2_3_1	*Conus geographus*
11	HIJWH8M02G3L2Y_1_1	*Conus geographus*
12	HIJWH8M02IP0VO_3_1	*Conus geographus*
13	HIJWH8M02JLLA6_4_1	*Conus geographus*
14	HIJWH8M02J5SK4_1_1	*Conus geographus*
15	HIJWH8M02JRLB1_3_1	*Conus geographus*
16	HIJWH8M02JZ8H6_1_1	*Conus geographus*
17	HIJWH8M02JW3PP_5_1	*Conus geographus*
18	HIJWH8M02I6SKI_3_1	*Conus geographus*
19	HIJWH8M02HPJVS_2_1	*Conus geographus*
20	HIJWH8M02FKRG6_3_1	*Conus geographus*
21	HIJWH8M02F4C30_2_1	*Conus geographus*
22	HIJWH8M02JJORL_3_1	*Conus geographus*

Table S3: Sequences Analyzed

23	HIJWH8M01EES7E_1_1	*Conus geographus*
24	HIJWH8M02H5K2R_5_1	*Conus geographus*
25	HIJWH8M02I14C7_3_1	*Conus geographus*
26	HIJWH8M02G2UVI_1_1	*Conus geographus*
27	HIJWH8M02I6CC7_3_1	*Conus geographus*
28	HIJWH8M02GL5W7_3_1	*Conus geographus*
29	HIJWH8M02I7NXV_1_1	*Conus geographus*
30	HIJWH8M02JBFJ3_1_1	*Conus geographus*
31	HIJWH8M01D1AHE_3_1	*Conus geographus*
32	HIJWH8M02I52K5_2_1	*Conus geographus*
33	HIJWH8M02IXQ27_2_1	*Conus geographus*
34	HIJWH8M01D8DZ4_2_1	*Conus geographus*
35	HIJWH8M02HAB0X_3_1	*Conus geographus*
36	HIJWH8M02GUPT4_3_1	*Conus geographus*
37	HIJWH8M02G19JV_1_1	*Conus geographus*
38	HIJWH8M02GGN2L_2_1	*Conus geographus*
39	HIJWH8M01EWPQ7_3_1	*Conus geographus*
40	HIJWH8M02FX3F7_1_1	*Conus geographus*

**Superfamily O1**		
1	HIJWH8M02JZTHI_6_1	*Conus geographus*
2	HIJWH8M02JZK5W_3_1	*Conus geographus*
3	HIJWH8M02JVNED_6_1	*Conus geographus*
4	HIJWH8M02JWJR3_6_1	*Conus geographus*
5	HIJWH8M02JAINF_5_1	*Conus geographus*
6	HIJWH8M02JG1TL_1_1	*Conus geographus*
7	HIJWH8M01EEV6S_1_1	*Conus geographus*
8	HIJWH8M02HBFST_6_1	*Conus geographus*
9	HIJWH8M02G875N_4_1	*Conus geographus*
10	HIJWH8M01EMW3Y_6_1	*Conus geographus*
11	HIJWH8M02IN5V7_4_1	*Conus geographus*
12	HIJWH8M02JYLQV_4_1	*Conus geographus*
13	HIJWH8M02JZDZO_3_1	*Conus geographus*
14	HIJWH8M02JCA7J_2_1	*Conus geographus*

**Superfamily O2**

Table S3: Sequences Analyzed

1	HIJWH8M02INOZN_5_1	Conus geographus
2	HIJWH8M02IA26Z_3_1	Conus geographus
3	HIJWH8M02H2DBM_4_1	Conus geographus
4	HIJWH8M02JXPBK_5_1	Conus geographus
5	HIJWH8M01EPYIR_1_1	Conus geographus

**Superfamily H**		
1	GQN8GVN01A4P6D_5	*Conus marmoreus*
2	GQN8GVN01BZIRB_6	*Conus marmoreus*
3	GQN8GVN01BEXD3_2	*Conus marmoreus*
4	GQN8GVN01BR1EH_5	*Conus marmoreus*

**Superfamily I2**		
1	GQN8GVN01B1USL_2	*Conus marmoreus*
2	GQN8GVN01EHT3W_6	*Conus marmoreus*
3	GQN8GVN01DUTWJ_1	*Conus marmoreus*
4	GP9D1AM02FSD58_5	*Conus marmoreus*
5	GQN8GVN01BRTG9_1	*Conus marmoreus*
6	GQN8GVN01DURZX_2	*Conus marmoreus*
7	GP9D1AM02G0HRD_3	*Conus marmoreus*
8	GQN8GVN01EHXYR_4	*Conus marmoreus*
9	GP9D1AM02JBC72_2	*Conus marmoreus*
10	GP9D1AM02JK2G3_2	*Conus marmoreus*

**Superfamily M**		
1	GP9D1AM02J2AF2_2	*Conus marmoreus*
2	GQN8GVN01ASJRW_3	*Conus marmoreus*
3	GQN8GVN01BDVUB_1	*Conus marmoreus*
4	GQN8GVN01BMJ2S_5	*Conus marmoreus*
5	GQN8GVN01D2BF9_5	*Conus marmoreus*
6	GQN8GVN01CU573_2	*Conus marmoreus*
7	GQN8GVN01C3E2A_6	*Conus marmoreus*
8	GQN8GVN01A4DBM_1	*Conus marmoreus*
9	GP9D1AM02FRKX7_1	*Conus marmoreus*
10	GQN8GVN01BN1YV_4	*Conus marmoreus*
11	GQN8GVN01EQTDC_5	*Conus marmoreus*

Table S3: Sequences Analyzed

12	GP9D1AM02JRQ2O_5	*Conus marmoreus*
13	GQN8GVN01BVSBZ_5	*Conus marmoreus*
14	GQN8GVN01AU3E6_6	*Conus marmoreus*
15	GQN8GVN01AOQWD_5	*Conus marmoreus*
16	GP9D1AM02F5D0W_4	*Conus marmoreus*
17	GQN8GVN01DY44H_1	*Conus marmoreus*
18	GQN8GVN01DE1RJ_1	*Conus marmoreus*
19	GP9D1AM02JUWP2_3	*Conus marmoreus*
20	GP9D1AM02IPEH7_4	*Conus marmoreus*
21	GP9D1AM02G808R_4	*Conus marmoreus*

**Superfamily O1**		
1	GQN8GVN01A2VRC_3	*Conus marmoreus*
2	GQN8GVN01DZMN1_4	*Conus marmoreus*
3	GQN8GVN01AN4X8_2	*Conus marmoreus*
4	GQN8GVN01CH6HM_4	*Conus marmoreus*
5	GQN8GVN01A0MAY_6	*Conus marmoreus*
6	GP9D1AM02F8EAM_3	*Conus marmoreus*
7	GQN8GVN01A775H_5	*Conus marmoreus*
8	GQN8GVN01AGGOV_4	*Conus marmoreus*
9	GQN8GVN01AMCDW_2	*Conus marmoreus*
10	GQN8GVN01A3WFQ_1	*Conus marmoreus*
11	GQN8GVN01DGPH6_2	*Conus marmoreus*
12	GQN8GVN01AMRDU_4	*Conus marmoreus*
13	GQN8GVN01DIO91_5	*Conus marmoreus*
14	GP9D1AM02GTD0A_3	*Conus marmoreus*

**Superfamily O2**		
1	GQN8GVN01CDX65_6	*Conus marmoreus*
2	GQN8GVN01D2F94_3	*Conus marmoreus*
3	GQN8GVN01ES8IP_1	*Conus marmoreus*
4	GQN8GVN01EEUS6_3	*Conus marmoreus*
5	GQN8GVN01ECY4L_6	*Conus marmoreus*
6	GQN8GVN01BV7HS_3	*Conus marmoreus*
7	GQN8GVN01B8474_1	*Conus marmoreus*
8	GQN8GVN01A9HDN_3	*Conus marmoreus*

Table S3: Sequences Analyzed

9	GQN8GVN01EO7KE_3	*Conus marmoreus*
10	GQN8GVN01B89JR_2	*Conus marmoreus*

**Superfamily T**		
1	GQN8GVN01DVA4R_1	*Conus marmoreus*
2	GQN8GVN01ENSOE_4	*Conus marmoreus*
3	GQN8GVN01DJ2AK_3	*Conus marmoreus*
4	GQN8GVN01CJJ3R_3	*Conus marmoreus*
5	GQN8GVN01DLQE1_6	*Conus marmoreus*
6	GQN8GVN01DFNB1_3	*Conus marmoreus*
7	GQN8GVN01BSLGW_3	*Conus marmoreus*
8	GQN8GVN01BVS05_3	*Conus marmoreus*
9	GP9D1AM02HWNIN_2	*Conus marmoreus*
10	GQN8GVN01AITBR_1	*Conus marmoreus*
11	GQN8GVN01ARITI_3	*Conus marmoreus*
12	GQN8GVN01AGW3M_6	*Conus marmoreus*

Advanced Snakes

**CRiSPs**		
1	EF599322.1	*Austrelaps superbus*
2	DQ917520.1	*Demansia vestigiata*
3	DQ084039.1	*Hoplocephalus stephensii*
4	AF384220.1	*Laticauda semifasciata*
5	AY324325.1	*Naja atra*
6	AY261468.1	*Naja atra*
7	EU938340.1	*Naja kaouthia*
8	EU938339.1	*Naja kaouthia*
9	DQ084038.1	*Notechis scutatus*
10	AY299475.1	*Ophiophagus hannah*
11	AY181984.1	*Ophiophagus hannah*
12	FJ767755.1	*Ophiophagus hannah*
13	DQ084036.1	*Oxyuranus microlepidotus*
14	DQ084035.1	*Oxyuranus scutellatus*
15	DQ139896.1	*Oxyuranus microlepidotus*

Table S3: Sequences Analyzed

16	AY072695.1	*Pseudechis australis*
17	AY072696.1	*Pseudechis porphyriacus*
18	DQ084037.1	*Pseudonaja textilis*
19	DQ917546.1	*Tropidechis carinatus*
20	DQ917547.1	*Rhinoplocephalus nigrescens*
21	AF384218.1	*Agkistrodon blomhoffi*
22	AY181982.1	*Agkistrodon piscivorus*
23	AY181983.1	*Crotalus atrox*
24	DQ464263.1	*Sistrurus catenatus*
25	AF384219.1	*Trimeresurus flavoviridis*
26	AY261467.1	*Trimeresurus jerdonii*
27	AY423708.1	*Trimeresurus stejnegeri*
28	AM937250.1	*Vipera berus*
29	GU065320.1	*Cerberus rynchops*
30	GU065319.1	*Cerberus rynchops*
31	GU065318.1	*Cerberus rynchops*
32	GU065317.1	*Cerberus rynchops*
33	DQ139891.1	*Dispholidus typus*
34	DQ139890.1	*Dispholidus typus*
35	DQ139891.1	*Dispholidus typus*
36	DQ139893.1	*Enhydris polylepis*
37	DQ139892.1	*Enhydris polylepis*
38	DQ139894.1	*Leioheterodon madagascariensis*
39	DQ139895.1	*Liophis poecilogyrus*
40	DQ912659.1	*Philodryas olfersii*
41	DQ139898.1	*Philodryas olfersii*
42	DQ139897.1	*Philodryas olfersii*
43	AY093955.1	*Rhabdophis tigrinus*
44	DQ139899.1	*Rhabdophis tigrinus*
45	DQ139900.1	*Telescopus dhara*
46	DQ139901.1	*Trimorphodon biscutatus*

**SVMPs**		
1	AJ223283.1	*Agkistrodon acutus*
2	AJ223284.1	*Agkistrodon acutus*
3	U18233.1	*Agkistrodon contortrix*
4	U18234.1	*Agkistrodon contortrix*

Table S3: Sequences Analyzed

5	AF212305.1	*Agkistrodon contortrix*
6	AB078904.1	*Agkistrodon contortrix*
7	U86634.1	*Agkistrodon contortrix*
8	FJ429179.1	*Agkistrodon piscivorus*
9	FJ429180.1	*Agkistrodon piscivorus*
10	AB078906.1	*Agkistrodon piscivorus*
11	AY885661.1	*Bitis arietans*
12	AY442287.1	*Bitis gabonica*
13	DQ247725.1	*Bothrops asper*
14	DQ247726.1	*Bothrops asper*
15	AF450503.1	*Bothrops erythromelas*
16	AF490533.1	*Bothrops insularis*
17	AY736107.1	*Bothrops insularis*
18	X68251.1	*Bothrops jararaca*
19	AF056025.2	*Bothrops jararaca*
20	AF345931.1	*Bothrops jararaca*
21	DQ375440.1	*Bothrops jararaca*
22	DQ375442.1	*Bothrops jararaca*
23	AY255005.1	*Bothrops jararacussu*
24	AF226274.1	*Bothrops neuwiedi*
25	L08780.1	*Calloselasma rhodostoma*
26	M89784.1	*Crotalus atrox*
27	U01234.1	*Crotalus atrox*
28	U01235.1	*Crotalus atrox*
29	U01236.1	*Crotalus atrox*
30	S77086.1	*Crotalus atrox*
31	AB042840.1	*Crotalus atrox*
32	AB257084.1	*Crotalus atrox*
33	FJ654661.1	*Crotalus durissus collilineatus*
34	DQ164402.1	*Crotalus durissus durissus*
35	DQ164403.1	*Crotalus durissus durissus*
36	DQ418751.1	*Crotalus durissus durissus*
37	DQ224420.1	*Crotalus durissus terrificus*
38	AF378288.1	*Crotalus molossus molossus*
39	AF378289.1	*Crotalus molossus molossus*
40	AF378290.1	*Crotalus molossus molossus*
41	AF378291.1	*Crotalus molossus molossus*

Table S3: Sequences Analyzed

42	DQ137798.1	*Daboia russelii*
43	DQ137799.1	*Daboia russelii*
44	AF141379.1	*Deinagkistrodon acutus*
45	AF162086.1	*Deinagkistrodon acutus*
46	AF174391.1	*Deinagkistrodon acutus*
47	AF174392.1	*Deinagkistrodon acutus*
48	AF098309.1	*Deinagkistrodon acutus*
49	AF098310.1	*Deinagkistrodon acutus*
50	AF099085.1	*Deinagkistrodon acutus*
51	AF099086.1	*Deinagkistrodon acutus*
52	AF117636.1	*Deinagkistrodon acutus*
53	AF117637.1	*Deinagkistrodon acutus*
54	AY566610.1	*Deinagkistrodon acutus*
55	DQ263750.1	*Deinagkistrodon acutus*
56	D32212.1	*Echis carinatus*
57	AM039691.1	*Echis ocellatus*
58	AM039692.1	*Echis ocellatus*
59	AM039693.1	*Echis ocellatus*
60	AM039694.1	*Echis ocellatus*
61	AM039695.1	*Echis ocellatus*
62	AM039696.1	*Echis ocellatus*
63	AM039697.1	*Echis ocellatus*
64	AM039698.1	*Echis ocellatus*
65	AM039699.1	*Echis ocellatus*
66	AM039700.1	*Echis ocellatus*
67	AM039701.1	*Echis ocellatus*
68	AF367868.1	*Gloydius halys*
69	D28870.1	*Gloydius halys*
70	AF051787.1	*Gloydius halys*
71	AF051788.1	*Gloydius halys*
72	AF051789.1	*Gloydius halys*
73	AF051790.1	*Gloydius halys*
74	AY071905.1	*Gloydius halys*
75	DQ457057.1	*Gloydius halys*
76	AF284093.1	*Gloydius halys*
77	AY204244.1	*Gloydius saxatilis*
78	DQ444299.1	*Gloydius shedaoensis*

Table S3: Sequences Analyzed

79	AY204245.1	*Gloydius ussuriensis*
80	X97894.1	*Macrovipera lebetina*
81	AY339162.1	*Macrovipera lebetina*
82	AY835996.1	*Macrovipera lebetina*
83	AY835997.1	*Macrovipera lebetina*
84	AY987816.1	*Macrovipera lebetina*
85	AB848272.1	*Ovophis okinavensis*
86	AB984586.1	*Protobothrops elegans*
87	DQ464251.1	*Sistrurus catenatus edwardsi*
88	AB059571.1	*Trimeresurus elegans*
89	AB059572.1	*Trimeresurus elegans*
90	AB051849.1	*Trimeresurus flavoviridis*
91	AB665726.1	*Trimeresurus flavoviridis*
92	AB848125.1	*Trimeresurus flavoviridis*
93	AB848126.1	*Trimeresurus flavoviridis*
94	AB985242.1	*Trimeresurus flavoviridis*
95	AY037808.1	*Trimeresurus jerdonii*
96	AB074143.1	*Trimeresurus jerdonii*
97	AB074144.1	*Trimeresurus jerdonii*
98	AB052155.1	*Trimeresurus jerdonii*
99	AY267902.1	*Trimeresurus jerdonii*
100	AY364231.1	*Trimeresurus jerdonii*
101	X77089.1	*Trimeresurus jerdonii*
102	X91190.1	*Trimeresurus jerdonii*
103	AF011909.1	*Trimeresurus jerdonii*
104	DQ464249.1	*Trimeresurus jerdonii*
105	DQ464254.1	*Trimeresurus jerdonii*
106	DQ464255.1	*Trimeresurus jerdonii*
107	X51530.1	*Trimeresurus jerdonii*
108	DQ195153.1	*Trimeresurus jerdonii*
109	DQ195154.1	*Trimeresurus jerdonii*
110	DQ335449.1	*Trimeresurus jerdonii*
111	KM886926.1	*Vipera ammodytes ammodytes*

**Cytotoxic 3FTx**		
1	U42585.1	*Naja atra*
2	U58487.1	*Naja atra*

Table S3: Sequences Analyzed

3	U58486.1	*Naja atra*
4	U58488.2	*Naja atra*
5	AF031474.1	*Naja atra*
6	U42586.1	*Naja atra*
7	U58490.1	*Naja atra*
8	Z54230.1	*Naja atra*
9	U58481.1	*Naja atra*
10	Z54229.1	*Naja atra*
11	U58489.1	*Naja atra*
12	U58485.1	*Naja atra*
13	Y12491.1	*Naja atra*
14	AF031473.1	*Naja atra*
15	U58482.1	*Naja atra*
16	U42583.1	*Naja atra*
17	U58484.1	*Naja atra*
18	U77487.1	*Naja atra*
19	U58483.1	*Naja atra*
20	AF295330.1	*Naja kaouthia*
21	AF295119.1	*Naja kaouthia*
22	AJ007796.1	*Naja naja*
23	AJ007797.1	*Naja naja*
24	Y08727.1	*Naja naja*
25	X94316.1	*Naja naja*
26	X94317.1	*Naja naja*
27	Z54226.1	*Naja naja*
28	L04640.1	*Naja naja sputatrix*
29	U86588.1	*Naja sputatrix*
30	U86593.1	*Naja sputatrix*
31	U86594.1	*Naja sputatrix*
32	U86589.1	*Naja sputatrix*
33	U86595.1	*Naja sputatrix*
34	U86591.1	*Naja sputatrix*
35	AF276222.1	*Naja sputatrix*
36	U86596.1	*Naja sputatrix*
37	U86597.1	*Naja sputatrix*

**Kappa 3FTx**		
1	AY057872.1	*Bungarus candidus*

Table S3: Sequences Analyzed

2	AY057873.1	*Bungarus candidus*
3	Y08721.1	*Bungarus multicinctus*
4	Y12266.1	*Bungarus multicinctus*
5	X51413.1	*Bungarus multicinctus*
6	AJ223187.1	*Bungarus multicinctus*
7	X51412.1	*Bungarus multicinctus*
8	Y12265.1	*Bungarus multicinctus*
9	GU190803.1	*Bungarus flaviceps*
10	Y11769.2	*Bungarus multicinctus*

**Type I α-neurotoxic 3FTx**		
1	AB017928.1	*Laticauda semifasciata*
2	AB017929.1	*Laticauda semifasciata*
3	AB017930.1	*Laticauda semifasciata*
4	AB017931.1	*Laticauda semifasciata*
5	AB017932.1	*Laticauda semifasciata*
6	AB017933.1	*Laticauda colubrina*
7	AB017934.1	*Laticauda colubrina*
8	AB017935.1	*Laticauda colubrina*
9	AB017936.1	*Laticauda colubrina*
10	AB017937.1	*Laticauda colubrina*
11	AB017938.1	*Laticauda colubrina*
12	AB017939.1	*Laticauda colubrina*
13	AB017940.1	*Laticauda colubrina*
14	AB017941.1	*Laticauda colubrina*
15	AB017942.1	*Laticauda colubrina*
16	AB017943.1	*Laticauda colubrina*
17	AB017944.1	*Laticauda colubrina*
18	AB017945.1	*Laticauda colubrina*
19	AB017946.1	*Laticauda colubrina*
20	AB017947.1	*Laticauda colubrina*
21	AB017949.1	*Laticauda colubrina*
22	AB017950.1	*Laticauda colubrina*
23	AB017951.1	*Laticauda colubrina*
24	AB017952.1	*Laticauda colubrina*
25	AB017953.1	*Laticauda colubrina*
26	AB017954.1	*Laticauda colubrina*
27	AB017955.1	*Laticauda laticaudata*

Table S3: Sequences Analyzed

28	AB017956.1	*Laticauda laticaudata*
29	AB017957.1	*Laticauda laticaudata*
30	AB017958.1	*Laticauda laticaudata*
31	AB017959.1	*Laticauda laticaudata*
32	AB017960.1	*Laticauda laticaudata*
33	AB017961.1	*Laticauda laticaudata*
34	AB017962.1	*Laticauda laticaudata*
35	AB017963.1	*Laticauda laticaudata*
36	AB017964.1	*Laticauda laticaudata*
37	AF023271.1	*Naja sputatrix*
38	AF023272.1	*Naja sputatrix*
39	AF031472.1	*Naja atra*
40	AF068052.1	*Naja naja*
41	AF088997.1	*Naja naja*
42	AF088998.1	*Naja naja*
43	AF159539.1	*Lapemis hardwickii*
44	AF159540.1	*Lapemis hardwickii*
45	AF161797.1	*Naja atra*
46	AJ239050.1	*Naja atra*
47	AY471578.1	*Naja atra*
48	AY471579.1	*Naja atra*
49	AY691664.1	*Oxyuranus scutellatus*
50	AY742211.1	*Acalyptophis peroni*
51	DQ066882.1	*Naja kaouthia*
52	DQ085855.1	*Oxyuranus scutellatus*
53	DQ917497.1	*Oxyuranus microlepidotus*
54	DQ917498.1	*Pseudechis australis*
55	DQ917499.1	*Pseudechis porphyriacus*
56	DQ917500.1	*Tropidechis carinatus*
57	DQ917501.1	*Tropidechis carinatus*
58	DQ917502.1	*Rhinoplocephalus nigrescens*
59	DQ917503.1	*Rhinoplocephalus nigrescens*
60	DQ917504.1	*Hoplocephalus stephensii*
61	DQ917505.1	*Notechis scutatus*
62	DQ917517.1	*Demansia vestigiata*
63	EF121774.1	*Hydrophis cyanocinctus*
64	EF405869.1	*Lapemis hardwickii*
65	EF405870.1	*Lapemis hardwickii*
66	EF599317.1	*Austrelaps superbus*

Table S3: Sequences Analyzed

67	EU003102.1	*Austrelaps labialis*
68	EU003103.1	*Austrelaps labialis*
69	EU029751.1	*Oxyuranus microlepidotus*
70	EU029752.1	*Oxyuranus microlepidotus*
71	EU196555.1	*Walterinnesia aegyptia*
72	FJ752480.1	*Drysdalia coronoides*
73	FJ752481.1	*Drysdalia coronoides*
74	FJ752482.1	*Drysdalia coronoides*
75	FJ752483.1	*Drysdalia coronoides*
76	FJ752484.1	*Drysdalia coronoides*
77	FJ752486.1	*Drysdalia coronoides*
78	FJ752487.1	*Drysdalia coronoides*
79	FJ752488.1	*Drysdalia coronoides*
80	FJ752489.1	*Drysdalia coronoides*
81	FJ752490.1	*Drysdalia coronoides*
82	FJ790447.1	*Suta nigriceps*
83	FJ790448.1	*Suta nigriceps*
84	FJ790449.1	*Suta nigriceps*
85	FJ790450.1	*Suta nigriceps*
86	FJ790451.1	*Suta nigriceps*
87	FJ790452.1	*Suta nigriceps*
88	FJ790454.1	*Suta nigriceps*
89	FJ790455.1	*Suta nigriceps*
90	GAHB01000003.1	*Cacophis squamulosus*
91	GAHB01000005.1	*Cacophis squamulosus*
92	GAHB01000006.1	*Cacophis squamulosus*
93	GAHB01000007.1	*Cacophis squamulosus*
94	GAHB01000008.1	*Cacophis squamulosus*
95	GAHF01000009.1	*Hemiaspis signata*
96	GAHF01000010.1	*Hemiaspis signata*
97	GAHF01000011.1	*Hemiaspis signata*
98	GAHF01000012.1	*Hemiaspis signata*
99	GAHF01000013.1	*Hemiaspis signata*
100	GAHF01000014.1	*Hemiaspis signata*
101	GAHJ01000011.1	*Vermicella annulata*
102	GAHJ01000012.1	*Vermicella annulata*
103	GAHJ01000013.1	*Vermicella annulata*
104	GAHJ01000014.1	*Vermicella annulata*
105	GAHJ01000015.1	*Vermicella annulata*

Table S3: Sequences Analyzed

106	U42582.1	*Naja atra*
107	U58519.1	*Naja atra*
108	U58520.1	*Naja atra*
109	U58521.1	*Naja atra*
110	U77490.1	*Naja atra*
111	U77491.1	*Naja atra*
112	U77492.1	*Naja atra*
113	X02533.1	*Laticauda semifasciata*
114	X13372.1	*Aipysurus laevis*
115	X13373.1	*Aipysurus laevis*
116	X16950.1	*Laticauda semifasciata*
117	GAHA01000012.1	*Simoselaps roperi*
118	GAHA01000014.1	*Simoselaps roperi*
119	GAHA01000015.1	*Simoselaps roperi*
120	GAHA01000016.1	*Simoselaps roperi*
121	GAHA01000013.1	*Simoselaps roperi*
122	DQ902576.1	*Ophiophagus hannah*
123	AY354200.1	*Ophiophagus hannah*

**Type II α-neurotoxic 3FTx**		
1	AB015512.1	*Laticauda semifasciata*
2	AB015513.1	*Laticauda semifasciata*
3	AF026893.1	*Naja sputatrix*
4	AF056400.1	*Bungarus multicinctus*
5	AF056401.1	*Bungarus multicinctus*
6	AF056402.1	*Bungarus multicinctus*
7	AF056403.1	*Bungarus multicinctus*
8	AF056404.1	*Bungarus multicinctus*
9	AF056405.1	*Bungarus multicinctus*
10	AF056406.1	*Bungarus multicinctus*
11	AF056407.1	*Bungarus multicinctus*
12	AF056408.1	*Bungarus multicinctus*
13	AF056409.1	*Bungarus multicinctus*
14	AF056410.1	*Bungarus multicinctus*
15	AF056411.1	*Bungarus multicinctus*
16	AF056412.1	*Bungarus multicinctus*
17	AF056413.1	*Bungarus multicinctus*
18	AF056414.1	*Bungarus multicinctus*

Table S3: Sequences Analyzed

19	AF056415.1	*Bungarus multicinctus*
20	AF056416.1	*Bungarus multicinctus*
21	AF056417.1	*Bungarus multicinctus*
22	AF082982.1	*Pseudonaja textilis*
23	AF142323.1	*Bungarus multicinctus*
24	AF159537.1	*Lapemis hardwickii*
25	AF159538.1	*Lapemis hardwickii*
26	AJ007766.1	*Bungarus multicinctus*
27	AY057874.1	*Bungarus multicinctus*
28	AY596926.1	*Ophiophagus hannah*
29	AY596927.1	*Ophiophagus hannah*
30	AY596928.1	*Ophiophagus hannah*
31	AY596929.1	*Ophiophagus hannah*
32	AY596930.1	*Ophiophagus hannah*
33	AY596932.1	*Ophiophagus hannah*
34	AY596933.1	*Ophiophagus hannah*
35	DQ273567.1	*Ophiophagus hannah*
36	DQ273568.1	*Ophiophagus hannah*
37	DQ273569.1	*Ophiophagus hannah*
38	DQ273570.1	*Ophiophagus hannah*
39	DQ273571.1	*Ophiophagus hannah*
40	DQ273572.1	*Ophiophagus hannah*
41	DQ902574.1	*Ophiophagus hannah*
42	DQ917508.1	*Notechis scutatus*
43	DQ917509.1	*Tropidechis carinatus*
44	DQ917510.1	*Pseudechis australis*
45	DQ917511.1	*Pseudonaja textilis*
46	DQ917512.1	*Oxyuranus microlepidotus*
47	DQ917513.1	*Oxyuranus microlepidotus*
48	DQ917514.1	*Oxyuranus scutellatus*
49	DQ917515.1	*Demansia vestigiata*
50	DQ917516.1	*Demansia vestigiata*
51	EF405871.1	*Lapemis hardwickii*
52	EF599318.1	*Austrelaps superbus*
53	EF599319.1	*Austrelaps superbus*
54	EU003085.1	*Austrelaps labialis*
55	EU003086.1	*Austrelaps labialis*
56	EU003087.1	*Austrelaps labialis*
57	EU003088.1	*Austrelaps labialis*

Table S3: Sequences Analyzed

58	EU003089.1	*Austrelaps labialis*
59	EU003090.1	*Austrelaps labialis*
60	EU003091.1	*Austrelaps labialis*
61	EU003092.1	*Austrelaps labialis*
62	EU003093.1	*Austrelaps labialis*
63	EU003094.1	*Austrelaps labialis*
64	EU003095.1	*Austrelaps labialis*
65	EU003096.1	*Austrelaps labialis*
66	EU003097.1	*Austrelaps labialis*
67	EU003098.1	*Austrelaps labialis*
68	EU003101.1	*Austrelaps labialis*
69	EU029749.1	*Oxyuranus microlepidotus*
70	EU029750.1	*Oxyuranus microlepidotus*
71	FJ481928.1	*Drysdalia coronoides*
72	FJ752455.1	*Drysdalia coronoides*
73	FJ752456.1	*Drysdalia coronoides*
74	FJ752457.1	*Drysdalia coronoides*
75	FJ752458.1	*Drysdalia coronoides*
76	FJ752459.1	*Drysdalia coronoides*
77	FJ752460.1	*Drysdalia coronoides*
78	FJ752461.1	*Drysdalia coronoides*
79	FJ752462.1	*Drysdalia coronoides*
80	FJ752463.1	*Drysdalia coronoides*
81	FJ752464.1	*Drysdalia coronoides*
82	FJ752465.1	*Drysdalia coronoides*
83	FJ752466.1	*Drysdalia coronoides*
84	FJ752467.1	*Drysdalia coronoides*
85	FJ752468.1	*Drysdalia coronoides*
86	FJ752469.1	*Drysdalia coronoides*
87	FJ752470.1	*Drysdalia coronoides*
88	FJ790456.1	*Suta nigriceps*
89	FJ790457.1	*Suta nigriceps*
90	FJ952515.1	*Ophiophagus hannah*
91	GAGZ01000001.1	*Acanthophis wellsei*
92	GAGZ01000002.1	*Acanthophis wellsei*
93	GAGZ01000003.1	*Acanthophis wellsei*
94	GAGZ01000004.1	*Acanthophis wellsei*
95	GAGZ01000005.1	*Acanthophis wellsei*
96	GAGZ01000006.1	*Acanthophis wellsei*

Table S3: Sequences Analyzed

97	GAGZ01000007.1	*Acanthophis wellsei*
98	GAGZ01000008.1	*Acanthophis wellsei*
99	GAGZ01000009.1	*Acanthophis wellsei*
100	GAGZ01000010.1	*Acanthophis wellsei*
101	GAHA01000001.1	*Simoselaps roperi*
102	GAHA01000002.1	*Simoselaps roperi*
103	GAHA01000006.1	*Simoselaps roperi*
104	GAHA01000007.1	*Simoselaps roperi*
105	GAHA01000008.1	*Simoselaps roperi*
106	GAHC01000001.1	*Denisonia devisi*
107	GAHC01000002.1	*Denisonia devisi*
108	GAHC01000003.1	*Denisonia devisi*
109	GAHC01000004.1	*Denisonia devisi*
110	GAHD01000001.1	*Echiopsis curta*
111	GAHD01000002.1	*Echiopsis curta*
112	GAHD01000003.1	*Echiopsis curta*
113	GAHD01000004.1	*Echiopsis curta*
114	GAHD01000005.1	*Echiopsis curta*
115	GAHD01000006.1	*Echiopsis curta*
116	GAHE01000001.1	*Furina ornata*
117	GAHE01000002.1	*Furina ornata*
118	GAHE01000003.1	*Furina ornata*
119	GAHE01000004.1	*Furina ornata*
120	GAHE01000005.1	*Furina ornata*
121	GAHE01000006.1	*Furina ornata*
122	GAHE01000007.1	*Furina ornata*
123	GAHE01000008.1	*Furina ornata*
124	GAHE01000009.1	*Furina ornata*
125	GAHE01000010.1	*Furina ornata*
126	GAHE01000011.1	*Furina ornata*
127	GAHE01000012.1	*Furina ornata*
128	GAHE01000013.1	*Furina ornata*
129	GAHE01000014.1	*Furina ornata*
130	GAHE01000015.1	*Furina ornata*
131	GAHE01000016.1	*Furina ornata*
132	GAHF01000001.1	*Hemiaspis signata*
133	GAHF01000002.1	*Hemiaspis signata*
134	GAHF01000003.1	*Hemiaspis signata*
135	GAHF01000004.1	*Hemiaspis signata*

Table S3: Sequences Analyzed

136	GAHF01000005.1	*Hemiaspis signata*
137	GAHF01000006.1	*Hemiaspis signata*
138	GAHF01000007.1	*Hemiaspis signata*
139	GAHF01000008.1	*Hemiaspis signata*
140	GAHG01000001.1	*Hoplocephalus bungaroides*
141	GAHG01000002.1	*Hoplocephalus bungaroides*
142	GAHH01000034.1	*Pseudonaja modesta*
143	GAHH01000039.1	*Pseudonaja modesta*
144	GAHH01000040.1	*Pseudonaja modesta*
145	GAHH01000041.1	*Pseudonaja modesta*
146	GAHH01000042.1	*Pseudonaja modesta*
147	GAHH01000043.1	*Pseudonaja modesta*
148	GAHH01000044.1	*Pseudonaja modesta*
149	GAHH01000045.1	*Pseudonaja modesta*
150	GAHH01000046.1	*Pseudonaja modesta*
151	GAHI01000001.1	*Suta fasciata*
152	GAHI01000002.1	*Suta fasciata*
153	GAHI01000003.1	*Suta fasciata*
154	GAHI01000004.1	*Suta fasciata*
155	GAHJ01000008.1	*Vermicella annulata*
156	GAHJ01000009.1	*Vermicella annulata*
157	GAHJ01000010.1	*Vermicella annulata*
158	Y17057.1	*Bungarus multicinctus*

**Type III α-neurotoxic 3FTx**		
1	AF082975.1	*Pseudonaja textilis*
2	AF082976.1	*Pseudonaja textilis*
3	AF082977.1	*Pseudonaja textilis*
4	AF082978.1	*Pseudonaja textilis*
5	AF082979.1	*Pseudonaja textilis*
6	AF082981.1	*Pseudonaja textilis*
7	DQ917506.1	*Pseudonaja textilis*
8	GAHA01000009.1	*Simoselaps roperi*
9	GAHA01000010.1	*Simoselaps roperi*
10	GAHA01000011.1	*Simoselaps roperi*
11	GAHB01000009.1	*Cacophis squamulosus*
12	GAHB01000010.1	*Cacophis squamulosus*
13	GAHB01000011.1	*Cacophis squamulosus*

Table S3: Sequences Analyzed

14	GAHE01000017.1	*Furina ornata*
15	GAHE01000018.1	*Furina ornata*
16	GAHE01000019.1	*Furina ornata*
17	GAHE01000020.1	*Furina ornata*
18	GAHE01000021.1	*Furina ornata*
19	GAHE01000022.1	*Furina ornata*
20	GAHE01000023.1	*Furina ornata*
21	GAHE01000024.1	*Furina ornata*
22	GAHE01000025.1	*Furina ornata*
23	GAHH01000001.1	*Pseudonaja modesta*
24	GAHH01000002.1	*Pseudonaja modesta*
25	GAHH01000003.1	*Pseudonaja modesta*
26	GAHH01000004.1	*Pseudonaja modesta*
27	GAHH01000005.1	*Pseudonaja modesta*
28	GAHH01000006.1	*Pseudonaja modesta*
29	GAHH01000007.1	*Pseudonaja modesta*
30	GAHH01000008.1	*Pseudonaja modesta*
31	GAHH01000009.1	*Pseudonaja modesta*
32	GAHH01000010.1	*Pseudonaja modesta*
33	GAHH01000011.1	*Pseudonaja modesta*
34	GAHH01000012.1	*Pseudonaja modesta*
35	GAHH01000013.1	*Pseudonaja modesta*
36	GAHH01000014.1	*Pseudonaja modesta*
37	GAHH01000015.1	*Pseudonaja modesta*
38	GAHH01000016.1	*Pseudonaja modesta*
39	GAHH01000017.1	*Pseudonaja modesta*
40	GAHH01000018.1	*Pseudonaja modesta*
41	GAHH01000019.1	*Pseudonaja modesta*
42	GAHH01000020.1	*Pseudonaja modesta*
43	GAHH01000021.1	*Pseudonaja modesta*
44	GAHH01000022.1	*Pseudonaja modesta*
45	GAHH01000023.1	*Pseudonaja modesta*
46	GAHH01000024.1	*Pseudonaja modesta*
47	GAHH01000025.1	*Pseudonaja modesta*
48	GAHH01000026.1	*Pseudonaja modesta*
49	GAHH01000027.1	*Pseudonaja modesta*
50	GAHH01000028.1	*Pseudonaja modesta*
51	GAHH01000029.1	*Pseudonaja modesta*
52	GAHH01000030.1	*Pseudonaja modesta*

Table S3: Sequences Analyzed

53	GAHH01000031.1	*Pseudonaja modesta*
54	GAHH01000032.1	*Pseudonaja modesta*
55	GAHH01000033.1	*Pseudonaja modesta*
56	GAHJ01000001.1	*Vermicella annulata*
57	GAHJ01000002.1	*Vermicella annulata*
58	GAHJ01000003.1	*Vermicella annulata*
59	GAHJ01000004.1	*Vermicella annulata*
60	GAHJ01000005.1	*Vermicella annulata*
61	GAHJ01000006.1	*Vermicella annulata*
62	GAHJ01000007.1	*Vermicella annulata*

***Crotalus o. helleri* Kallikreins**		
1	GAKS01000006.1	*Crotalus oreganus helleri*
2	GAKQ01000008.1	*Crotalus oreganus helleri*
3	GALC01000005.1	*Crotalus oreganus helleri*
4	GAKS01000007.1	*Crotalus oreganus helleri*
5	GAKR01000007.1	*Crotalus oreganus helleri*
6	GALC01000006.1	*Crotalus oreganus helleri*
7	GALC01000007.1	*Crotalus oreganus helleri*
8	GAKQ01000009.1	*Crotalus oreganus helleri*
9	GAKR01000008.1	*Crotalus oreganus helleri*
10	GAKS01000008.1	*Crotalus oreganus helleri*
11	GALC01000008.1	*Crotalus oreganus helleri*
12	GAKQ01000010.1	*Crotalus oreganus helleri*
13	GAKR01000009.1	*Crotalus oreganus helleri*
14	GAKS01000009.1	*Crotalus oreganus helleri*
15	GAKQ01000011.1	*Crotalus oreganus helleri*
16	GAKR01000011.1	*Crotalus oreganus helleri*
17	GALC01000009.1	*Crotalus oreganus helleri*
18	GAKS01000012.1	*Crotalus oreganus helleri*
19	GAKR01000010.1	*Crotalus oreganus helleri*
20	GAKS01000010.1	*Crotalus oreganus helleri*
21	GAKQ01000012.1	*Crotalus oreganus helleri*
22	GALC01000011.1	*Crotalus oreganus helleri*
23	GALC01000010.1	*Crotalus oreganus helleri*
24	GAKS01000011.1	*Crotalus oreganus helleri*

Table S3: Sequences Analyzed

***Crotalus o. helleri* β-defensins**		
1	GALC01000001.1	*Crotalus oreganus helleri*
2	GAKS01000001.1	*Crotalus oreganus helleri*
3	GAKS01000002.1	*Crotalus oreganus helleri*
4	GAKR01000001.1	*Crotalus oreganus helleri*
5	GAKQ01000001.1	*Crotalus oreganus helleri*
6	GAKQ01000002.1	*Crotalus oreganus helleri*
7	GAKR01000002.1	*Crotalus oreganus helleri*
8	GAKQ01000003.1	*Crotalus oreganus helleri*
9	GAKS01000003.1	*Crotalus oreganus helleri*

**Serine Proteases**		
1	HQ270467.1	*Agkistrodon piscivorus leucostoma*
2	KF410948.1	*Bothrops pictus pictobin*
3	AJ001209.1	*Agkistrodon acutus*
4	AF159057.1	*Deinagkistrodon acutus*
5	AF159059.1	*Deinagkistrodon acutus*
6	AF159060.1	*Deinagkistrodon acutus*
7	AJ006471.1	*Agkistrodon acutus*
8	AF333768.1	*Deinagkistrodon acutus*
9	AY861382.1	*Deinagkistrodon acutus*
10	AY861383.1	*Deinagkistrodon acutus*
11	AY861138.1	*Deinagkistrodon acutus*
12	EU681180.1	*Deinagkistrodon acutus*
13	HQ270465.1	*Daboia russellii siamensis*
14	DQ464238.1	*Sistrurus catenatus edwardsi*
15	DQ464239.1	*Sistrurus catenatus edwardsi*
16	DQ464242.1	*Sistrurus catenatus edwardsi*
17	DQ464243.1	*Sistrurus catenatus edwardsi*
18	DQ464244.1	*Sistrurus catenatus edwardsi*
19	DQ464245.1	*Sistrurus catenatus edwardsi*
20	DQ464247.1	*Sistrurus catenatus edwardsi*
21	DQ464246.1	*Sistrurus catenatus edwardsi*
22	HQ414117.1	*Crotalus adamanteus*
23	HQ414118.1	*Crotalus adamanteus*
24	HQ414121.1	*Crotalus adamanteus*
25	HQ414122.1	*Crotalus adamanteus*

Table S3: Sequences Analyzed

26	GU012098.1	*Echis coloratus*
27	GU012106.1	*Echis coloratus*
28	GU012107.1	*Echis coloratus*
29	GU012108.1	*Echis coloratus*
30	GU012112.1	*Echis coloratus*
31	GU592440.1	*Echis coloratus*
32	GU012100.1	*Echis coloratus*
33	AF490536.1	*Bothrops insularis*
34	AF056033.1	*Agkistrodon halys pallas*
35	AF015727.1	*Agkistrodon halys pallas*
36	AJ006472.1	*Agkistrodon halys pallas*
37	AJ001210.1	*Agkistrodon halys pallas*
38	AY225505.1	*Agkistrodon halys pallas*
39	JQ965747.1	*Agkistrodon halys pallas*
40	KC537786.1	*Agkistrodon halys pallas*
41	KC537787.1	*Agkistrodon halys pallas*
42	D67082.1	*Trimeresurus gramineus*
43	D67083.1	*Trimeresurus gramineus*
44	D67085.1	*Trimeresurus gramineus*
45	AB031394.1	*Bothrops jararaca*
46	AY251282.1	*Bothrops jararacussu*
47	AJ251153.1	*Vipera lebetina*
48	GU570565.1	*Macrovipera lebetina*
49	GU570566.1	*Macrovipera lebetina*
50	GU570567.1	*Macrovipera lebetina*
51	L07308.1	*Calloselasma rhodostoma*
52	AJ243757.1	*Agkistrodon blomhoffii*
53	AB749761.1	*Agkistrodon blomhoffii*
54	AB749762.1	*Agkistrodon blomhoffii*
55	AB984572.1	*Protobothrops elegans*
56	D67079.1	*Trimeresurus flavoviridis*
57	D67080.1	*Trimeresurus flavoviridis*
58	AB848162.1	*Trimeresurus flavoviridis*
59	AF292110.1	*Trimeresurus jerdonii*
60	EU400543.1	*Trimeresurus jerdonii*
61	X83221.1	*Protobothrops mucrosquamatus*
62	X83222.1	*Protobothrops mucrosquamatus*
63	X83223.1	*Protobothrops mucrosquamatus*
64	X83224.1	*Protobothrops mucrosquamatus*

Table S3: Sequences Analyzed

65	X83225.1	*Protobothrops mucrosquamatus*
66	U31417.1	*Protobothrops mucrosquamatus*
67	AF098262.1	*Protobothrops mucrosquamatus*
68	AF098263.1	*Protobothrops mucrosquamatus*
69	AY434726.1	*Gloydius shedaoensis*
70	AF017736.1	*Gloydius brevicaudus*
71	AF176679.1	*Gloydius brevicaudus*
72	AF545575.1	*Trimeresurus stejnegeri*
73	AF545576.1	*Trimeresurus stejnegeri*
74	AF545577.1	*Trimeresurus stejnegeri*
75	AF545579.1	*Trimeresurus stejnegeri*
76	AF395763.1	*Trimeresurus stejnegeri*
77	AF395765.1	*Trimeresurus stejnegeri*
78	AF395768.1	*Trimeresurus stejnegeri*
79	AF395771.1	*Trimeresurus stejnegeri*
80	AF395773.1	*Trimeresurus stejnegeri*
81	AF395774.1	*Trimeresurus stejnegeri*
82	AF395775.1	*Trimeresurus stejnegeri*
83	AF395776.1	*Trimeresurus stejnegeri*
84	AF395777.1	*Trimeresurus stejnegeri*
85	AF395778.1	*Trimeresurus stejnegeri*
86	AF395779.1	*Trimeresurus stejnegeri*
87	AF395781.1	*Trimeresurus stejnegeri*
88	AY954040.1	*Crotalus durissus terrificus*
89	EU360951.1	*Crotalus durissus terrificus*
90	EU360952.1	*Crotalus durissus terrificus*
91	EU360954.1	*Crotalus durissus terrificus*
92	U32937.1	*Agkistrodon ussuriensis*
93	AF018568.1	*Gloydius ussuriensis*
94	AF444251.1	*Gloydius ussuriensis*
95	AF336126.1	*Gloydius ussuriensis*
96	AF370124.1	*Gloydius ussuriensis*
97	AF227153.1	*Crotalus atrox*
98	EF690365.1	*Cryptelytrops albolabris*
99	EF690366.1	*Cryptelytrops albolabris*

**PLA2 Type I**		
1	AM492700.1	*Naja atra*

Table S3: Sequences Analyzed

2	AB011388.1	*Naja kaouthia*
3	AB011389.1	*Naja kaouthia*
4	X63947.1	*Naja Naja*
5	X73225.1	*Naja Naja*
6	X77755.1	*Naja Naja*
7	L42004.1	*Naja Naja*
8	L42005.1	*Naja Naja*
9	L42006.1	*Naja Naja*
10	AY422776.1	*Naja sagittifera*
11	AY433936.1	*Naja sagittifera*
12	AY443496.1	*Naja sagittifera*
13	AY422775.2	*Naja sagittifera*
14	AY862399.1	*Naja sagittifera*
15	AY862400.1	*Naja sagittifera*
16	AF101235.1	*Naja sputatrix*
17	AF101236.1	*Naja sputatrix*
18	AY081146.1	*Bungarus caeruleus*
19	AY081147.1	*Bungarus caeruleus*
20	AF297663.1	*Bungarus caeruleus*
21	AY455756.1	*Bungarus caeruleus*
22	AY455754.1	*Bungarus caeruleus*
23	AY057880.1	*Bungarus candidus*
24	AY057882.1	*Bungarus candidus*
25	AY142324.1	*Bungarus candidus*
26	AY425948.1	*Bungarus candidus*
27	AY425950.1	*Bungarus candidus*
28	AB158299.1	*Bungarus candidus*
29	DQ768745.1	*Bungarus fasciatus*
30	AB112356.1	*Bungarus flaviceps*
31	AB112357.1	*Bungarus flaviceps*
32	GU190818.1	*Bungarus flaviceps*
33	GU190819.1	*Bungarus flaviceps*
34	GU190820.1	*Bungarus flaviceps*
35	AB112359.1	*Bungarus flaviceps*
36	AB112360.1	*Bungarus flaviceps*
37	GU190815.1	*Bungarus flaviceps*
38	GU190816.1	*Bungarus flaviceps*
39	GU190817.1	*Bungarus flaviceps*
40	X53407.1	*Bungarus multicinctus*

Table S3: Sequences Analyzed

41	X98205.1	*Bungarus multicinctus*
42	AJ251360.1	*Bungarus multicinctus*
43	AJ242011.1	*Bungarus multicinctus*
44	AJ242012.1	*Bungarus multicinctus*
45	AJ242013.1	*Bungarus multicinctus*
46	AJ251220.1	*Bungarus multicinctus*
47	AJ251221.1	*Bungarus multicinctus*
48	AJ251222.1	*Bungarus multicinctus*
49	AJ251227.1	*Bungarus multicinctus*
50	AJ223249.1	*Bungarus multicinctus*
51	AJ431711.1	*Bungarus multicinctus*
52	AJ431707.1	*Bungarus multicinctus*
53	AJ431708.1	*Bungarus multicinctus*
54	AJ431709.1	*Bungarus multicinctus*
55	AJ431710.1	*Bungarus multicinctus*
56	AJ431712.1	*Bungarus multicinctus*
57	DQ810293.1	*Bungarus multicinctus*
58	X53406.1	*Bungarus multicinctus*
59	GAGZ01000025.1	*Acanthophis wellsi*
60	GAGZ01000030.1	*Acanthophis wellsi*
61	GAGZ01000026.1	*Acanthophis wellsi*
62	GAGZ01000029.1	*Acanthophis wellsi*
63	GAHA01000028.1	*Brachyurophis roperi*
64	GAHC01000018.1	*Denisonia devisi*
65	GAHC01000017.1	*Denisonia devisi*
66	GAHG01000028.1	*Hoplocephalus bungaroides*
67	GAHG01000023.1	*Hoplocephalus bungaroides*
68	GAHG01000024.1	*Hoplocephalus bungaroides*
69	GAHG01000025.1	*Hoplocephalus bungaroides*
70	GAHG01000026.1	*Hoplocephalus bungaroides*
71	GAHI01000022.1	*Suta fasciata*
72	GAHI01000028.1	*Suta fasciata*
73	GAHI01000024.1	*Suta fasciata*
74	GAHI01000029.1	*Suta fasciata*
75	GAHI01000026.1	*Suta fasciata*
76	GAHI01000021.1	*Suta fasciata*
77	GAHI01000023.1	*Suta fasciata*
78	GAGZ01000028.1	*Acanthophis wellsi*
79	GAGZ01000024.1	*Acanthophis wellsi*

Table S3: Sequences Analyzed

80	GAHI01000025.1	*Suta fasciata*
81	GAHI01000027.1	*Suta fasciata*
82	EU003106.1	*Austrelaps labialis*
83	EU003107.1	*Austrelaps labialis*
84	EU003108.1	*Austrelaps labialis*
85	EU003109.1	*Austrelaps labialis*
86	EU003105.1	*Austrelaps labialis*
87	AF184137.1	*Austrelaps labialis*
88	AF184138.1	*Austrelaps labialis*
89	AF184139.1	*Austrelaps labialis*
90	AF184140.1	*Austrelaps labialis*
91	AF184141.1	*Austrelaps labialis*
92	EF599325.1	*Austrelaps labialis*
93	AF184127.1	*Austrelaps superbus*
94	AF184128.1	*Austrelaps superbus*
95	AF184129.1	*Austrelaps superbus*
96	AF184130.1	*Austrelaps superbus*
97	AF184131.1	*Austrelaps superbus*
98	AF184132.1	*Austrelaps superbus*
99	AF184133.1	*Austrelaps superbus*
100	AF184134.1	*Austrelaps superbus*
101	AF184135.1	*Austrelaps superbus*
102	AF184136.1	*Austrelaps superbus*
103	AY178051.1	*Austrelaps superbus*
104	EF599324.1	*Austrelaps superbus*
105	EF599326.1	*Austrelaps superbus*
106	DQ917537.1	*Demansia vestigiata*
107	DQ917538.1	*Demansia vestigiata*
108	DQ917539.1	*Demansia vestigiata*
109	DQ917540.1	*Demansia vestigiata*
110	FJ752448.1	*Drysdalia coronoides*
111	FJ752450.1	*Drysdalia coronoides*
112	EF164997.1	*Hoplocephalus stephensii*
113	EF164998.1	*Hoplocephalus stephensii*
114	EF164996.1	*Hoplocephalus stephensii*
115	X12605.1	*Notechis scutatus*
116	X14043.1	*Notechis scutatus*
117	DQ085831.1	*Notechis scutatus*
118	DQ085832.1	*Notechis scutatus*

Table S3: Sequences Analyzed

119	DQ085833.1	*Notechis scutatus*
120	DQ917544.1	*Notechis scutatus*
121	DQ085822.1	*Oxyuranus microlepidotus*
122	DQ085823.1	*Oxyuranus microlepidotus*
123	DQ085824.1	*Oxyuranus microlepidotus*
124	DQ085825.1	*Oxyuranus microlepidotus*
125	DQ085820.1	*Oxyuranus microlepidotus*
126	AY691661.1	*Oxyuranus scutellatus*
127	AY691662.1	*Oxyuranus scutellatus*
128	DQ085819.1	*Oxyuranus scutellatus*
129	DQ085817.1	*Oxyuranus scutellatus*
130	DQ085818.1	*Oxyuranus scutellatus*
131	AY691657.1	*Oxyuranus scutellatus*
132	AY691658.1	*Oxyuranus scutellatus*
133	DQ085843.1	*Pseudechis australis*
134	DQ085842.1	*Pseudechis australis*
135	DQ085844.1	*Pseudechis australis*
136	DQ085846.1	*Pseudechis australis*
137	DQ085847.1	*Pseudechis australis*
138	DQ085848.1	*Pseudechis australis*
139	AB531118.1	*Pseudechis australis*
140	AB531119.1	*Pseudechis australis*
141	AB531121.1	*Pseudechis australis*
142	AB531123.1	*Pseudechis australis*
143	AB531124.1	*Pseudechis australis*
144	AB531125.1	*Pseudechis australis*
145	AB531126.1	*Pseudechis australis*
146	DQ085849.1	*Pseudechis porphyriacus*
147	DQ085850.1	*Pseudechis porphyriacus*
148	DQ085852.1	*Pseudechis porphyriacus*
149	DQ085853.1	*Pseudechis porphyriacus*
150	DQ085854.1	*Pseudechis porphyriacus*
151	DQ085851.1	*Pseudechis porphyriacus*
152	EU523132.1	*Pseudonaja textilis*
153	DQ085829.1	*Pseudonaja textilis*
154	DQ085828.1	*Pseudonaja textilis*
155	DQ085830.1	*Pseudonaja textilis*
156	DQ917542.1	*Rhinoplocephalus nigrescens*
157	DQ917541.1	*Rhinoplocephalus nigrescens*

Table S3: Sequences Analyzed

158	DQ917543.1	*Rhinoplocephalus nigrescens*
159	DQ917549.1	*Rhinoplocephalus nigrescens*
160	FJ790477.1	*Suta nigriceps*
161	FJ790471.1	*Suta nigriceps*
162	FJ790472.1	*Suta nigriceps*
163	FJ790473.1	*Suta nigriceps*
164	FJ790474.1	*Suta nigriceps*
165	FJ790475.1	*Suta nigriceps*
166	FJ790476.1	*Suta nigriceps*
167	DQ917545.1	*Tropidechis carinatus*
168	AB062445.1	*Laticauda colubrina*
169	AB062446.1	*Laticauda colubrina*
170	AB062447.1	*Laticauda colubrina*
171	AB062448.1	*Laticauda colubrina*
172	Y00377.1	*Laticauda laticaudata*
173	AB062441.1	*Laticauda laticaudata*
174	AB062442.1	*Laticauda laticaudata*
175	AB062443.1	*Laticauda laticaudata*
176	AB062444.1	*Laticauda laticaudata*
177	AB037409.1	*Laticauda semifasciata*
178	AB037410.1	*Laticauda semifasciata*
179	AB037411.1	*Laticauda semifasciata*
180	AB037412.1	*Laticauda semifasciata*
181	AB037413.1	*Laticauda semifasciata*
182	AB037414.1	*Laticauda semifasciata*
183	AB037415.1	*Laticauda semifasciata*
184	AB062439.1	*Laticauda semifasciata*
185	AB062440.1	*Laticauda semifasciata*
186	AB120375.1	*Laticauda semifasciata*
187	AB120376.1	*Laticauda semifasciata*
188	AB037219.2	*Laticauda semifasciata*
189	AF144319.1	*Lapemis hardwickii*
190	AF205378.1	*Lapemis hardwickii*
191	EF405872.1	*Lapemis hardwickii*
192	EF405876.1	*Lapemis hardwickii*
193	AF144320.1	*Lapemis hardwickii*
194	AY561165.1	*Aipysurus eydouxii*
195	AY561169.1	*Aipysurus eydouxii*
196	AY561168.1	*Aipysurus eydouxii*

Table S3: Sequences Analyzed

197	AY561157.1	*Aipysurus eydouxii*
198	AY561154.1	*Aipysurus eydouxii*
199	AY561159.1	*Aipysurus eydouxii*
200	AY561167.1	*Aipysurus eydouxii*
201	AY561163.1	*Aipysurus eydouxii*
202	AY561161.1	*Aipysurus eydouxii*
203	AY561156.1	*Aipysurus eydouxii*
204	AY561155.1	*Aipysurus eydouxii*
205	AY561164.1	*Aipysurus eydouxii*
206	AY561166.1	*Aipysurus eydouxii*
207	AY561162.1	*Aipysurus eydouxii*
208	AY561160.1	*Aipysurus eydouxii*
209	AY561158.1	*Aipysurus eydouxii*
210	X12604.1	*Aipysurus laevis*
211	GAGZ01000027.1	*Acanthophis wellsi*
212	GAHA01000030.1	*Brachyurophis roperi*
213	GAHA01000029.1	*Brachyurophis roperi*
214	GAHC01000016.1	*Denisonia devisi*
215	GAHG01000027.1	*Hoplocephalus bungaroides*
216	GAHH01000068.1	*Pseudonaja modesta*
217	GAHH01000065.1	*Pseudonaja modesta*
218	GAHH01000066.1	*Pseudonaja modesta*
219	GAHH01000067.1	*Pseudonaja modesta*
220	GAHH01000069.1	*Pseudonaja modesta*
221	AF184142.1	*Austrelaps superbus*
222	AF184143.1	*Austrelaps superbus*
223	AB158302.1	*Bungarus candidus*
224	AY057881.1	*Bungarus candidus*
225	DQ917536.1	*Demansia vestigiata*
226	DQ917535.1	*Demansia vestigiata*
227	DQ917534.1	*Demansia vestigiata*
228	EF164994.1	*Hoplocephalus stephensii*
229	EF164995.1	*Hoplocephalus stephensii*
230	EF164993.1	*Hoplocephalus stephensii*
231	DQ085834.1	*Notechis scutatus*
232	DQ085835.1	*Notechis scutatus*
233	AF297034.1	*Ophiophagus hannah*
234	AF302908.1	*Ophiophagus hannah*
235	DQ085821.1	*Oxyuranus microlepidotus*

Table S3: Sequences Analyzed

236	DQ085814.1	*Oxyuranus scutellatus*
237	DQ085815.1	*Oxyuranus scutellatus*
238	DQ085816.1	*Oxyuranus scutellatus*
239	AY691659.1	*Oxyuranus scutellatus*
240	AY691660.1	*Oxyuranus scutellatus*
241	DQ085813.1	*Oxyuranus scutellatus*
242	AB531117.1	*Pseudechis cf. australis*
243	AF082983.1	*Pseudonaja textilis*
244	AF082984.1	*Pseudonaja textilis*
245	DQ085826.1	*Pseudonaja textilis*
246	DQ085827.1	*Pseudonaja textilis*
247	DQ085836.1	*Tropidechis carinatus*
248	DQ085837.1	*Tropidechis carinatus*
249	DQ085838.1	*Tropidechis carinatus*
250	DQ085839.1	*Tropidechis carinatus*
251	DQ085840.1	*Tropidechis carinatus*
252	DQ085841.1	*Tropidechis carinatus*
253	AB078348.1	*Laticauda semifasciata*
254	AB078347.1	*Laticauda semifasciata*

**PLA2 Type II**		
1	EU029748.1	*Azemiops feae*
2	AY885662.1	*Bitis arietans*
3	AY764137.1	*Bothriechis schlegelii*
4	AY355168.1	*Bothriechis schlegelii*
5	JQ661499.1	*Bothropoides diporus*
6	JQ661500.1	*Bothropoides diporus*
7	X76289.1	*Bothrops jararacussu*
8	AY145836.1	*Bothrops jararacussu*
9	GQ850458.1	*Bothrops neuwiedi*
10	EU029756.1	*Causus rhombeatus*
11	FJ754186.1	*Cerastes cerastes*
12	FJ754187.1	*Cerastes cerastes*
13	AY764138.1	*Cerrophidion godmani*
14	HQ414104.1	*Crotalus adamanteus*
15	AF269131.1	*Crotalus atrox*
16	GQ466583.1	*Crotalus durissus*

Table S3: Sequences Analyzed

17	X12606.1	*Crotalus durissus terrificus*
18	GQ168369.1	*Crotalus horridus atricaudatus*
19	GQ168368.1	*Crotalus horridus atricaudatus*
20	JF895776.1	*Crotalus oreganus*
21	JF895774.1	*Crotalus oreganus helleri*
22	JF895777.1	*Crotalus oreganus helleri*
23	U01026.1	*Crotalus scutulatus*
24	AF403137.1	*Crotalus viridis*
25	AY120875.1	*Crotalus viridis viridis*
26	X68386.1	*Daboia russelli*
27	X77649.1	*Deinagkistrodon acutus*
28	AY268946.2	*Echis carinatus*
29	AF539919.1	*Echis carinatus sochureki*
30	AF253049.1	*Echis coloratus*
31	AF253050.1	*Echis coloratus*
32	FM177947.1	*Echis ocellatus*
33	AF015242.1	*Gloydius halys*
34	AY434727.1	*Gloydius shedaoensis*
35	GQ265920.1	*Lachesis muta*
36	FM202092.1	*Macrovipera lebetina*
37	FJ905321.1	*Macrovipera lebetina*
38	EU421953.1	*Macrovipera lebetina*
39	D49390.1	*Ovophis okinavensis*
40	AB219805.1	*Protobothrops elegans*
41	AF408409.1	*Protobothrops mucrosquamatus*
42	AY355169.1	*Sistrurus catenatus*
43	AY508692.1	*Sistrurus catenatus tergeminus*
44	AY508693.1	*Sistrurus catenatus tergeminus*
45	EU369752.1	*Sistrurus miliarius*
46	EU369753.1	*Sistrurus miliarius*
47	EU369754.1	*Sistrurus miliarius*
48	EU369755.1	*Sistrurus miliarius*
49	AY355166.1	*Sistrurus miliarius*
50	AY355178.1	*Trimeresurus borneensis*
51	AY355179.1	*Trimeresurus borneensis*
52	D10721.1	*Trimeresurus flavoviridis*
53	AY764141.1	*Trimeresurus gracilis*

Table S3: Sequences Analyzed

54	D31774.1	*Trimeresurus gramineus*
55	D31778.1	*Trimeresurus gramineus*
56	D31780.1	*Trimeresurus gramineus*
57	AF504039.1	*Trimeresurus jerdonii*
58	X77646.2	*Trimeresurus mucrosquamatus*
59	D49388.1	*Trimeresurus okinavensis*
60	AY355174.1	*Trimeresurus puniceus*
61	AY355173.1	*Trimeresurus puniceus*
62	AY355175.1	*Trimeresurus puniceus*
63	AY211939.1	*Trimeresurus stejnegeri*
64	AY211940.1	*Trimeresurus stejnegeri*
65	AY211942.1	*Trimeresurus stejnegeri*
66	AY211943.1	*Trimeresurus stejnegeri*
67	X52241.1	*Vipera ammodytes*
68	AJ580275.1	*Vipera aspis*
69	AJ580191.1	*Vipera berus*
70	AM707023.1	*Vipera nikolskii*
71	U60017.1	*Vipera palaestinae*
72	AF091854.1	*Vipera palaestinae*
73	AF091855.1	*Vipera palaestinae*
74	GQ304905.1	*Vipera ursinii*
75	AJ223188.1	*Agkistrodon acutus*
76	U21335.1	*Agkistrodon contortrix*
77	AF374236.1	*Bothriechis schlegelii*
78	JQ661501.1	*Bothropoides diporus*
79	AF109911.1	*Bothrops asper*
80	EU336943.1	*Bothrops asper*
81	AY431026.1	*Bothrops atrox*
82	AY185200.1	*Bothrops jararacussu*
83	AY299391.1	*Bothrops jararacussu*
84	X78599.2	*Bothrops jararacussu*
85	AF145759.1	*Bothrops moojeni*
86	AF145781.1	*Bothrops neuwiedi pauloensis*
87	AF104066.1	*Calloselasma rhodostoma*
88	AF374237.1	*Cerrophidion godmani*
89	AY764140.1	*Cerrophidion godmani*
90	AF269130.1	*Crotalus atrox*

Table S3: Sequences Analyzed

91	AF269132.1	*Deinagkistrodon acutus*
92	AM411360.1	*Deinagkistrodon acutus*
93	AF015243.1	*Gloydius halys*
94	AB219806.1	*Protobothrops elegans*
95	AB219807.1	*Protobothrops elegans*
96	DQ299948.1	*Protobothrops mucrosquamatus*
97	AY355177.1	*Trimeresurus borneensis*
98	D10718.1	*Trimeresurus flavoviridis*
99	D10719.1	*Trimeresurus flavoviridis*
100	D13383.1	*Trimeresurus flavoviridis*
101	D13384.1	*Trimeresurus flavoviridis*
102	AB470470.1	*Trimeresurus flavoviridis*
103	AY764142.1	*Trimeresurus gracilis*
104	D31775.1	*Trimeresurus gramineus*
105	D31779.1	*Trimeresurus gramineus*
106	D31781.1	*Trimeresurus gramineus*
107	D49389.1	*Trimeresurus okinavensis*
108	AY355171.1	*Trimeresurus puniceus*
109	AY355172.1	*Trimeresurus puniceus*
110	AY211934.1	*Trimeresurus stejnegeri*
111	AY211935.1	*Trimeresurus stejnegeri*
112	AY211936.1	*Trimeresurus stejnegeri*
113	AY211937.1	*Trimeresurus stejnegeri*
114	AY211938.1	*Trimeresurus stejnegeri*
115	X53036.1	*Vipera ammodytes*
116	X84017.1	*Vipera ammodytes*
117	AJ580254.1	*Vipera ammodytes*
118	AJ580257.1	*Vipera ammodytes*
119	AJ580258.1	*Vipera ammodytes*
120	AJ580260.1	*Vipera ammodytes*
121	AJ580261.1	*Vipera ammodytes*
122	AJ580263.1	*Vipera ammodytes*
123	AJ580264.1	*Vipera ammodytes*
124	AJ580272.1	*Vipera ammodytes*
125	AJ580251.1	*Vipera ammodytes*
126	AJ580252.1	*Vipera ammodytes*
127	AJ580267.1	*Vipera ammodytes*

Table S3: Sequences Analyzed

128	AJ580266.1	*Vipera ammodytes*
129	AJ580270.1	*Vipera ammodytes*
130	AJ580269.1	*Vipera ammodytes*
131	AJ580271.1	*Vipera ammodytes*
132	AJ580255.1	*Vipera ammodytes*
133	AJ580259.1	*Vipera ammodytes*
134	AJ580256.1	*Vipera ammodytes*
135	AJ580268.1	*Vipera ammodytes*
136	AJ580262.1	*Vipera ammodytes*
137	AJ580265.1	*Vipera ammodytes*
138	AJ580243.1	*Vipera berus*
139	AJ580244.1	*Vipera berus*
140	AJ580245.1	*Vipera berus*
141	AJ580247.1	*Vipera berus*
142	AJ580248.1	*Vipera berus*
143	AJ580249.1	*Vipera berus*
144	AJ580250.1	*Vipera berus*
145	AJ580253.1	*Vipera berus*
146	AJ580246.1	*Vipera berus*
147	GQ304904.1	*Vipera ursinii*
148	FM177946.1	*Echis ocellatus*

**Psammophis monodomain SVMP**		
1	EU029730.1	*Psammophis mossambicus*
2	EU029731.1	*Psammophis mossambicus*
3	EU029732.1	*Psammophis mossambicus*
4	EU029726.1	*Psammophis mossambicus*
5	EU029733.1	*Psammophis mossambicus*
6	EU029728.1	*Psammophis mossambicus*
7	EU029727.1	*Psammophis mossambicus*
8	EU029729.1	*Psammophis mossambicus*
9	EU029708.1	*Psammophis mossambicus*
10	EU029710.1	*Psammophis mossambicus*
11	EU029709.1	*Psammophis mossambicus*
12	EU029722.1	*Psammophis mossambicus*
13	EU029723.1	*Psammophis mossambicus*
14	EU029724.1	*Psammophis mossambicus*

Table S3: Sequences Analyzed

15	EU029721.1	*Psammophis mossambicus*
16	EU029711.1	*Psammophis mossambicus*
17	EU029712.1	*Psammophis mossambicus*
18	EU029725.1	*Psammophis mossambicus*
19	EU029719.1	*Psammophis mossambicus*
20	EU029717.1	*Psammophis mossambicus*
21	EU029718.1	*Psammophis mossambicus*
22	EU029720.1	*Psammophis mossambicus*
23	EU029715.1	*Psammophis mossambicus*
24	EU029716.1	*Psammophis mossambicus*
25	EU029713.1	*Psammophis mossambicus*
26	EU029714.1	*Psammophis mossambicus*

**Lectins**		
1	HE800429.1	*Bitis gabonica*
2	HE800431.1	*Bitis gabonica*
3	KJ614391.1	*Gloydius intermedius*
4	AB036880.1	*Deinagkistrodon acutus*
5	AF102901.1	*Deinagkistrodon acutus*
6	AF463522.1	*Deinagkistrodon acutus*
7	AF176420.2	*Deinagkistrodon acutus*
8	AY091758.1	*Deinagkistrodon acutus*
9	AY091759.1	*Deinagkistrodon acutus*
10	AY091762.1	*Deinagkistrodon acutus*
11	AF540645.1	*Deinagkistrodon acutus*
12	AF540646.1	*Deinagkistrodon acutus*
13	GQ426542.1	*Daboia russellii*
14	GQ426544.1	*Daboia russellii*
15	GQ420352.1	*Daboia russellii*
16	GQ426536.1	*Daboia russellii*
17	GQ426548.1	*Daboia russellii*
18	DQ060414.1	*Daboia russellii*
19	DQ218272.1	*Daboia russellii*
20	GQ426534.1	*Daboia russellii*
21	GQ426538.1	*Daboia russellii*
22	GQ426540.1	*Daboia russellii*
23	GQ426546.1	*Daboia russellii*
24	DQ464258.1	*Sistrurus catenatus edwardsi*

Table S3: Sequences Analyzed

25	HQ414089.1	*Crotalus adamanteus*
26	HQ414092.1	*Crotalus adamanteus*
27	HQ414093.1	*Crotalus adamanteus*
28	HQ414096.1	*Crotalus adamanteus*
29	AB098253.1	*Echis multisquamatus*
30	AY429478.1	*Bitis gabonica*
31	AY429479.1	*Bitis gabonica*
32	AF190827.1	*Gloydius halys*
33	AY356354.1	*Gloydius halys*
34	AY346127.1	*Gloydius halys*
35	HE804182.1	*Gloydius halys*
36	AB848278.1	*Ovophis okinavensis*
37	AY962524.1	*Bothrops jararaca*
38	AB794990.1	*Bothrops jararaca*
39	AY578116.1	*Macrovipera lebetina*
40	EU085446.1	*Macrovipera lebetina*
41	EU085447.1	*Macrovipera lebetina*
42	EU085448.1	*Macrovipera lebetina*
43	EU085449.1	*Macrovipera lebetina*
44	EU085450.1	*Macrovipera lebetina*
45	EU085451.1	*Macrovipera lebetina*
46	EU085452.1	*Macrovipera lebetina*
47	EU085453.1	*Macrovipera lebetina*
48	EU085454.1	*Macrovipera lebetina*
49	EU085455.1	*Macrovipera lebetina*
50	EU085456.1	*Macrovipera lebetina*
51	EU085457.1	*Macrovipera lebetina*
52	EU085458.1	*Macrovipera lebetina*
53	EU085459.1	*Macrovipera lebetina*
54	EU085460.1	*Macrovipera lebetina*
55	EU085461.1	*Macrovipera lebetina*
56	KF836491.1	*Macrovipera lebetina*
57	KF836492.1	*Macrovipera lebetina*
58	KP202316.1	*Macrovipera lebetina*
59	KP202319.1	*Macrovipera lebetina*
60	KP202321.1	*Macrovipera lebetina*
61	AF244900.1	*Calloselasma rhodostoma*
62	AB019615.1	*Agkistrodon blomhoffi*
63	KM527183.1	*Philodryas chamissonis*

Table S3: Sequences Analyzed

64	DQ912660.1	*Philodryas chamissonis*
65	AB984598.1	*Protobothrops elegans*
66	D83331.1	*Trimeresurus flavoviridis*
67	AB046491.1	*Trimeresurus flavoviridis*
68	AY149341.1	*Trimeresurus flavoviridis*
69	AB848117.1	*Trimeresurus flavoviridis*
70	AB848118.1	*Trimeresurus flavoviridis*
71	AB848119.1	*Trimeresurus flavoviridis*
72	AB848120.1	*Trimeresurus flavoviridis*
73	AB848121.1	*Trimeresurus flavoviridis*
74	AB848123.1	*Trimeresurus flavoviridis*
75	AB985229.1	*Trimeresurus flavoviridis*
76	AB985243.1	*Trimeresurus flavoviridis*
77	GU146049.1	*Trimeresurus jerdonii*
78	GU136389.1	*Trimeresurus jerdonii*
79	AY099321.1	*Protobothrops mucrosquamatus*
80	AY390533.1	*Protobothrops mucrosquamatus*
81	AY871785.1	*Protobothrops mucrosquamatus*
82	AY254327.1	*Bitis arietans*
83	AY734997.1	*Daboia russellii*
84	JN582177.1	*Daboia russellii*
85	AF125309.1	*Gloydius halys*
86	JF895761.1	*Crotalus oreganus helleri*
87	JF895763.1	*Crotalus oreganus helleri*
88	JF895764.1	*Crotalus oreganus helleri*
89	AF354911.1	*Trimeresurus stejnegeri*
90	AF354918.1	*Trimeresurus stejnegeri*
91	AF354919.1	*Trimeresurus stejnegeri*
92	AF354920.1	*Trimeresurus stejnegeri*
93	AF354921.1	*Trimeresurus stejnegeri*
94	AF354922.1	*Trimeresurus stejnegeri*
95	AF354923.1	*Trimeresurus stejnegeri*
96	AF354924.1	*Trimeresurus stejnegeri*
97	Y16348.1	*Crotalus durissus*
98	AF541882.1	*Crotalus durissus*
99	AF541883.1	*Crotalus durissus*
100	EF690367.1	*Cryptelytrops albolabris*

Table S3: Sequences Analyzed

Legend

This table provides accession numbers of sequences analyzed in this study and the scientific names of animals from which they were sequenced. Alignments generated by these sequences can be found as the archived supplemental file.
